# Discovery of a new fibronectin-binding surface protein of *Streptococcus canis* with serum opacification activity through transposon directed insertion-site sequencing

**DOI:** 10.3389/fcimb.2026.1867913

**Published:** 2026-06-29

**Authors:** Miriam Katsburg, Anna Kopenhagen, Etienne Aubry, Mathias Müsken, Gloria Riebesell, Deborah Simmert, Sanja Haake, Inga Eichhorn, Silver A. Wolf, Simone Bergmann, Marcus Fulde

**Affiliations:** 1 Institute of Microbiology and Epizootics, Centre for Infection Medicine, Freie Universität Berlin, Berlin, Germany; 2 Veterinary Centre for Resistance Research (TZR), Freie Universität Berlin, Berlin, Germany; 3 Institute of Microbiology, Technische Universität Braunschweig, Braunschweig, Germany; 4 Central Facility for Microscopy, Helmholtz Centre for Infection Research, Braunschweig, Germany; 5 Genome Competence Centre (MF1), Robert Koch Institute (RKI), Berlin, Germany

**Keywords:** endothelial infection, endothelial wound healing, fibronectin, high-throughput screening, infective endocarditis, serum opacification, streptococcal adhesin, TRADIS

## Abstract

Infective endocarditis is a rare but severe disease in humans and dogs which can be caused by *Streptococcus canis*. To understand how *S. canis* can adhere to and invade the endocardium, we combined a high-throughput approach called transposon directed insertion-site sequencing (TraDIS) with a physiologically relevant endothelial cell infection model that incorporates venous-range shear stress to identify genes encoding factors relevant for bacterial adherence and invasion to vascular human cells under simulation of physiological blood flow conditions. A saturated transposon library of clinical strain IMT49926 was screened in a microfluidic infection assay, enabling genome-wide selection of mutants impaired in endothelial adhesion. Comparative analysis of input and non-adherent output pools revealed several candidate genes, including a fibronectin-binding LPXTG-anchored surface protein with high homology to streptococcal serum opacity factors (SOFs). Our findings identified ScSOF as a multifunctional surface protein that plays an important role in the infection potential of *S. canis*. It facilitates adhesion to endothelial cells, prevents endothelial wound closure, contributes to the streptococcal surface architecture, binds fibronectin, opacifies serum, and inhibits β-hemolytic activity. These properties make ScSOF a compelling candidate for further, more targeted studies to confirm it as a virulence factor in *S. canis* that can be used for vaccine development or therapeutic targeting.

## 1 Introduction

*Streptococcus canis* (*S. canis*) is an opportunistic pathogen that inhabits mucosal surfaces in up to 6.5% of healthy dogs but has been implicated in as many as 22.4% of streptococcal infections in dogs and even up to a quarter of canine infective endocarditis cases (Sykes et al., 2006; Lysková et al., 2007; Lamm et al., 2010). *S. canis* has been shown to be the cause of several cases of infective endocarditis (IE) in human patients as well, often after infection of a wound due to close contact with pet cats or dogs (MacDonald, 2010; Amsallem et al., 2014; Lacave et al., 2016; Mališová et al., 2019; Wang et al., 2024). IE is a life-threatening condition in both human and veterinary medicine, marked by a high mortality rate especially in canines due to difficulty in diagnosis and treatment (MacDonald, 2010; Reagan et al., 2022; Katsburg et al., 2023). Typical IE pathogenesis starts with entry of the bacteria into the bloodstream via wounds or surgical intervention sites (Moser et al., 2017). Some of the bacteria may escape phagocytosis in the bloodstream and can adhere to the endocardium to establish infection. Once the bacteria colonize the endocardium, they can form a structured, multilayered biofilm composed of bacterial aggregates thereby impairing the efficacy of antibiotic treatment (Lerche et al., 2021). The endocardium displays an increased vulnerability to infection due to prior damage in the endothelial layer, caused by common risk factors for IE such as obesity and age. In addition, mechanical stress mediated by high blood pressure, inflammation and infection are often responsible for endothelial damage (Witten et al., 2024). The bacterial factors mediating endothelial colonization of *S. canis* are largely unknown.

One contributing factor to adhesion and invasion of the host cells is the *S. canis* M protein SCM, which was previously implicated in IgG binding and immune evasion and thought to be involved in early colonization stages through interactions with host factors such as fibrinogen and plasminogen, depending on the M protein type (Fulde et al., 2011; Bergmann et al., 2017; Lapschies et al., 2023). However, the contribution of SCM to virulence in an *in vivo* murine model and an *in vitro* vaginal epithelial cell adherence assay was modest (Cornax et al., 2021). These studies suggest that additional adhesins beyond SCM play a role in *S. canis* pathogenicity.

Moreover, in several Gram-positive pathogens, such as *Staphylococcus aureus*, *Streptococcus mutans* and *Streptococcus pyogenes*, fibronectin-binding and fibrinogen-binding surface proteins have been described as important virulence determinants that contribute to endothelial colonization (Nygren et al., 1988; Talay et al., 1996; Douglas and Ford, 1997; Que et al., 2005; Fischetti, 2019; Widmer et al., 2006; Jung et al., 2009; Bedran et al., 2013; Oppegaard et al., 2017). Whether comparable mechanisms exist in *S. canis* is currently unknown. The aim of this study was to identify factors involved in the adhesion and invasion of *S. canis* in endothelial cells under more physiological conditions.

We established an *in vitro* infection model that closely reflects the dynamic blood flow environment encountered by endothelial cells within the vasculature. Endothelial cells are constantly influenced by hemodynamic forces, particularly shear stress generated by blood flow, which governs their structural and functional characteristics. To replicate these physiological conditions, we employed a microfluidic system (Ibidi) capable of applying defined shear stresses (6–10 dyn/cm²), thereby simulating the venous flow environment relevant for bloodstream infections such as endocarditis and septicaemia. This setup allows us to directly compare bacterial adherence and host cell responses under both flow and static conditions, enabling the identification of adherence factors that are specifically required for colonization under physiologically relevant shear stress. The use of microfluidics ensures consistent and reproducible flow conditions, supporting the formation of endothelial monolayers that closely mimic *in vivo* behavior (Jagau et al., 2019).

To systematically identify such factors, we applied a genome-wide functional screening approach, by using transposon directed insertion-site sequencing (TraDIS). TraDIS has already been successfully applied to identify essential bacterial genes in various growth conditions, such as oxidative stress or antibiotic pressure (Langridge et al., 2009; Barquist et al., 2016; Charbonneau et al., 2017, 2020). Previously, this technique was also used to identify virulence genes in various *in vivo* infection models (Moule et al., 2015; Weerdenburg et al., 2015). In accordance with the 3R principle, animal experiments should be replaced as much as possible by *in vitro* assays. This study is the first report of using TraDIS in combination with an *in vitro* endothelial cell infection assay under simulation of physiological blood flow to study relevant gene products for adherence and invasion.

The aim of this study was to identify genes involved in the adhesion and invasion of *S. canis* in endothelial cells under physiological flow conditions and to functionally characterize candidate virulence factors. To reach this aim, we successfully combined a human cell culture infection model system under defined physiological shear force with a transposon library- sequencing based- whole genome approach. This combination of highly advanced techniques provides a novel and highly sophisticated research system for identification of bacterial factors of highest relevance for vascular colonization. Using TraDIS screening, we identified a previously uncharacterized serum opacity factor-like protein, ScSOF, as a contributor to endothelial interaction, fibronectin binding and impairment of endothelial wound healing. These findings improve our understanding of *S. canis* pathogenesis and may support the development of future therapeutic or vaccine strategies targeting infective endocarditis.

## 2 Materials and methods

### 2.1 Bacterial strains and growth conditions

In this study, *S. canis* strain IMT49926 and its isogenic *sof* knockout mutant were used. Strain IMT49926 was isolated from a case of canine infective endocarditis during routine diagnostics at Freie Universität Berlin (Katsburg et al., 2023). Targeted mutagenesis of the *sof* gene was performed as described previously but using a newly constructed pGh9:Δs*of* vector (Lapschies et al., 2023). The regions 500 bp upstream and downstream of the *sof* gene were amplified from gDNA of IMT49926 using the primer pairs: 5’-GGCCATGGTAGGGGAACTAGAC-3’; 5’-CCCTATAGCCGACAGAT AATTTCC-3’ and 5´-GGGATATCGGAGCAGCAGGAC-3´; 5’-GGCCATGGGACGTTCATTTT GAG-3’. The two PCR products were digested using EcoRV and ligated with T4 Ligase. The resulting 1 kb region was amplified again with the previous primers 5’-GGCCATGGTAGGGGA ACTAGAC-3’ and 5’-GGCCATGGGACGTTCATTTTGAG-3’. The product was digested with NcoI and inserted into the previously described pGh9:ΔIS*S1* vector (Lapschies et al., 2023). 200 ng of the resulting plasmid was introduced into IMT49926 by electroporation. Genetic composition of the isogenic *sof* knockout mutant was confirmed via PCR using the primer pair mentioned above. Additional whole genome sequencing, genome assembly and annotation was performed as described in Katsburg et al., 2023 and confirmed that the double cross over event had taken place, creating an unmarked deletion mutant. The annotated genome of *S. canis* IMT49926 was visualized with Proksee using CGViewBuilder v1.1.2. This webtool was also applied to plot GC-content and GC skew. Blast comparisons with the genome of the isogenic deletion mutant *S. canis 49926 Δsof* confirmed no “off-site”-mutantion (E-value cut off of 0.1).

For serum opacification activity analysis, *S. suis* strain M10 was used as a positive control (Baums et al., 2006). Streptococci were grown on Columbia agar plates with 5% sheep blood (BD) or statically in Todd Hewitt broth (THB) (Roth) at 37 °C, unless indicated otherwise. *Escherichia coli* strains were routinely cultured in Luria-Bertani (LB) medium at 37 °C and 200 rpm unless indicated otherwise. In appropriate cases, antibiotics were added at the following concentrations: erythromycin (Erm), 150 µg/mL for *E. coli*, 2 µg/mL for *S. canis*. All bacterial cultures were executed in ambient air incubators.

### 2.2 Generation of transposon library

The transposon mutant library was generated using the temperature-sensitive plasmid pGh9:IS*S1* (Maguin et al., 1996; Charbonneau et al., 2017; Aubry et al., 2025), which carries the ermB resistance marker and a Gram-positive thermosensitive origin of replication. *S. canis* 49926 was transformed by electroporation and cultured at 30 °C to allow plasmid replication. Transposition was induced by heat shock at 40 °C, promoting chromosomal integration of the IS*S1* element. Mutants were pooled, and stored for further analysis.

### 2.3 *In vitro* HUVEC assays

Primary human umbilical vein endothelial cells (HUVEC, PromoCell, Germany) were cultured in endothelial cell growth medium (C-22010, PromoCell, Germany) and grown to confluency under flow conditions using three Ibidi µ-Slide I Luer channels connected in parallel to a software-controlled pneumatic pump (Ibidi, Germany). This setup generated a physiological shear stress that was gradually increased from three to 10 dyn/cm², after which cells were maintained at 10 dyn/cm² for 24 h. Subsequently, cells were infected with mid-log phase *S. canis* TraDIS library, *S. canis* wild type or ScSOF knockout strains at multiplicity of infection (MOI) 10 under a shear stress of 6 dyn/cm² for 2.5 h. Reproducibility of bacterial cell culture infection was achieved by photometrical assessment of the multiplicity of infection (MOI), which represents the ratio of infectious agents (here amount of *S. canis* bacteria and transposon library) to infection targets (here amount of A549 cells and HUVEC in an infection cavity). Initial determination of bacterial colony forming units (cfu) representing a specific OD_600nm_ was used to calculate the amount of bacteria covering the desired MOI for the amount eukaryotic cells seeded in one cavity. In order to reduce the amount of dead bacteria within the infection pool, the bacterial cultures were always harvested at an OD_600nm_ of 0.4, since growth curves confirmed that this OD value represents the exponential growth phase of *S. canis*.

Infection assays were performed in three biological replicates with technical duplicates. In addition, one extra slide per experiment was included for immunofluorescence staining to verify monolayer integrity and confirm bacterial adherence and invasion.

The infection assay using the *S. canis* TraDIS library was conducted in two consecutive rounds under increasing selective pressure. In general, HUVECs were infected with the *S. canis* TraDIS library in three biological replicates with two technical replicates using two slides, while a third slide was used for immunofluorescence analysis. After this so-called first selection round, adherent and non-adherent bacteria were harvested separately as bacterial output pools for TraDIS analysis. Non-adherent bacteria were recovered from the flow-through medium, regrown overnight in THB, and stored for genomic DNA extraction. The regrowth of the non-adherent mutant library prior to the second infection round was necessary to maintain the same infection MOI. In order to reduce the likelihood of misleading effects by stochastic underrepresentation of metabolic enzymes, we harvested bacteria strictly in the exponential phase rather than in the stationary phase. Nevertheless, bacterial regrowth might lead to loss of some specific metabolism mutants and therefore marks a limit of this experimental set up. After the infection, adherent bacteria, which were associated with the HUVEC monolayer, were detached together with host cells using Accutase, regrown overnight in THB, and likewise stored for genomic DNA extraction (as described in the section “DNA preparation and sequencing”).

After sequencing of the output pools representing the first round of infection, non-significant logFC values of sequence data demonstrated that after the first infection round the selection pressure was not sufficient. To increase selective pressure and enable more stringent discrimination between adherent and non-adherent mutants, a second round of infection was performed using only a pooled sample of non-adherent bacteria from the first round as inoculum. The infection procedure was repeated in three biological replicates. As before, adherent and non-adherent bacteria were collected separately, regrown overnight, and subjected to genomic DNA extraction. This approach enabled clear discrimination between bacteria that remained tightly associated with host cells and those that had lost adherence capacity due to transposon insertions. These cell infection analyses resulted in bacterial output pools named the first and the second selection.

Slides used for differential immunofluorescence staining (DIF) followed by microscopy were fixed with using 3% paraformaldehyde in PBS, and stained to assess both, bacterial association and cytoskeletal integrity of the host cells. The cytoskeleton was visualized using Alexa Fluor 488-conjugated phalloidin, while cell nuclei were stained with DAPI. *S. canis* bacteria were detected using a polyclonal *S. canis-*specific primary antibody raised in rabbit (Bergmann et al., 2017). Overlays of different fluorophore signals were used to distinguish between attached and internalized bacteria by first using an Alexa Fluor 488 secondary antibody to stain only extracellular bacteria, followed by an Alexa Fluor 568 secondary antibody after endothelial cell permeabilization with 0.01% Triton X-100.

In static infection assays, HUVECs were grown to confluency on 12 mm glass coverslips in a 24-well plate. Cells were then infected at various MOIs ranging from 0.5 to 5 for 1 hour, followed by replacement of the medium and incubation for an additional 2 hours. Immunofluorescent staining was performed as previously described. Images were acquired as z-stacks using a confocal laser scanning microscope (CLSM; Leica SP8) equipped with LAS X software (Leica, Germany), employing a 63x/0.75 oil immersion objective lens to achieve a total magnification of 630x.

### 2.4 Quantification of bacterial adhesion and invasion of HUVEC cells

Cell adherence and invasion were quantified in three independent biological experiments (biological replicates). For each biological replicate, infections were performed in technical replicates by serial connection of three µ-slides. On each slide, bacteria associated with approximately 12–15 host cells per field of view were counted across 12 randomly selected non-overlapping fields in the central region of the Ibidi flow chamber (or Slide) with a shear stress of 10 dyn/cm². Edge regions were avoided due to minor local variations in shear stress arising from slide geometry and connection area to the pump tubing (as indicated by Ibidi guidelines). All images were taken with 40× objective and were analyzed and counted using LAS X software to quantify bacteria associated with endothelial cells.

### 2.5 *In vitro* A549 infection assays

A549 lung epithelial cells were cultured in DMEM supplemented with 10% FBS. Cells were cultured at 37 °C and 5% CO_2_. Cells were seeded (1*10^4^ cells/well) on glass coverslips in a 24-well plate, grown to confluency and subsequently infected with *S. canis* wild type or the *sof* knockout strain at MOI 5 for 2.5 hours. Post-infection, cells were washed, fixed, and stained for bacterial association and host cytoskeletal integrity using polyclonal antibody against *S. canis* raised in rabbits, goat anti-rabbit Alexa Fluor 488 and Alexa Fluor 647 after permeabilization as well as Alexa Fluor 580-conjugated phalloidin as described in the HUVEC infection assay. DAPI was used to stain cell nuclei. For quantification, a similar infection was executed without the glass coverslips. Bacteria-infected wells were washed twice with PBS and lysed by 1% (w/v) saponin in H_2_O. Serial dilutions in PBS were plated out on THB-Agar. Colonies were counted after incubation for 48 hours at 37 °C. Cell culture infection was carried out in three biological replicates with three technical replicates for each biological replicate.

### 2.6 Chamber separation cell migration assay

Endothelial gap closure was assessed using the CSMA as described previously (Kopenhagen et al., 2022). Briefly, human umbilical vein endothelial cells (HUVECs; 3 × 10^5^ cells/mL) were seeded into Ibidi three-chamber silicone inlets placed on gelatin-coated culture dishes and cultured until reaching confluence. Subsequently, the confluent monolayers were exposed to laminar shear stress gradually increased from 3 to 10 dyn/cm² and then maintained at 10 dyn/cm² for up to 48 h. Cell adaptation to flow conditions was initiated at 3 dyn/cm² for 30 min, followed by stepwise increases of 1 dyn/cm² every 30 min until a final shear stress of 10 dyn/cm² was reached, as described by Jagau et al. (2019) and Kopenhagen et al. (2022). This gradual increase allowed HUVECs to adapt to shear stress, including the induction of mechanosensitive responses such as the expression of mechanoreceptors.

A shear stress of 10 dyn/cm² lies within the physiological range of arterial wall shear stress in human vasculature, which typically spans approximately 10–70 dyn/cm², whereas venous shear stress is generally lower, around 1–6 dyn/cm² (Samijo et al., 1998). Previous studies have demonstrated that such shear stress conditions are required for the activation of endothelial mechanosome complexes comprising mechanosensitive receptors, including PECAM-1, VE-cadherin, and VEGFR2. In addition, physiological flow has been shown to upregulate the expression of other shear-responsive receptors in vascular endothelial cells, such as integrins, ICAM-1, and VCAM-1 (Davies et al., 1997; Conway and Schwartz, 2012; Chistiakov et al., 2017). Following removal of the inlets, a defined 500 µm cell-free gap was generated between two adjacent monolayers, separated by the inlet’s central ridge. For wound healing analysis, both cell borders were visualized microscopically with actin and nuclei visualized by DAPI and Alexa Fluor 488-conjugated phalloidin, and images were acquired at three representative regions per sample. Gap closure and morphological changes were monitored at multiple time points. The extent of gap closure was quantified by determining the change in size of the cell-free area over time. The initial gap area at t=0 h corresponded to 0 % gap closure. All values were normalized to this reference to calculate the relative percentage of closure at each time point. Images were obtained by CLSM (SP8, Leica) and cell-free areas were quantified and normalized to baseline using LAS X software. To study the effects of *S. canis* infection, HUVECs were preconditioned under flow, followed by infection with *S. canis* IMT49926 or the 49926Δ*sof* mutant (MOI 5) under continuous shear stress. Media were exchanged periodically to limit bacterial overgrowth. Experiments were performed in triplicate and analyzed for effects on endothelial cell migration and morphology.

### 2.7 Differentiation between proliferating and migrating cells during gap closure

Endothelial cell proliferation and migration during gap closure were assessed by integrating the EdU click chemistry in the CSMA setup. An amount of 10 μM EdU solution was applied for labelling of proliferating cells (Kopenhagen et al., 2022). Endothelial cell nuclei were visualized by fluorescence microscopy and quantified by CLSM (Leica SP8, DMI8) with a 20×/0.75 IMM objective and 0.75 zoom factor). In combination with 6-FAM-azide, nuclei of proliferating cells appear in light red in fluorescence microscopic visualization, whereas migrating cells are counter-stained in blue by DAPI incubation. The proportion of proliferating versus migrating cells was quantified by counting red- and blue-fluorescing cell nuclei within the defined gap area at the indicated time points (6 h, 24 h, and 48 h). All experiments were performed in triplicate, and results are reported as mean ± SD. For each coverslip, a minimum of four randomly selected fields of view of the wound were imaged and used for nuclear quantification. Data are expressed as mean values with standard deviation. *) indicates p < 0.05, **) indicates p < 0.01, and ***) indicates p < 0.001, according to one-way ANOVA and Tukey HSD *post hoc* tests. For the 24 h samples homoscedasticity was not met (Levene p < 0.05); therefore, Welch’s ANOVA and Games-Howell *post hoc* testing were applied.

### 2.8 DNA preparation and sequencing

DNA was extracted from the mutant libraries after overnight growth at 37 °C in Todd Hewitt Broth. Bacterial cells were harvested by centrifugation and resuspended in 180 μL enzyme solution (20 mg/mL lysozyme, 30 mM Tris-Cl pH 8, 2 mM EDTA, 10% Triton X-100) for 1 h at 37 °C. Subsequently, 20 μL proteinase K and 1 μL RNase A (5 μg/μL) were added, followed by 200 μL AL buffer (QIAmp DNA Mini Kit, Qiagen) and incubation at 56 °C for 1 h. After adding 200 μL ethanol, the mixture was applied to a spin column for DNA purification, and the protocol of the kit was followed from here. DNA was eluted with 30 μL Milli-Q water. DNA was quantified using the Qubit dsDNA HS Assay and 1 µg of input DNA was used to start the library preparation. Fragmentation, A-tailing, and end repair, were performed using the NEBNext Ultra II FS DNA Library Prep Kit for Illumina (NEB #E7805, E6177), yielding fragments of approximately 400 bp.

A Y-adaptor was generated in-house using Illumina multiplexing adaptor sequences (Illumina) according to previous methods (Charbonneau et al., 2017). Adaptor ligation was executed by adding 15 µM of the adaptor to the fragmentation mixture, followed by NEBNext Ultra II Ligation Master Mix and NEBNext Ligation Enhancer. Samples were incubated at 20 °C for 15 minutes in a thermocycler. Purification by AMPure Xpbeads, PCR amplification, PCR clean up, and size selection were done following the method described in 2017 by Charbonnaeu and coworker. Final library concentration was measured using the Qubit dsDNA HS Assay and adjusted to yield 10 pM final load concentration for the pooled replicates.

To prepare for sequencing, the NextSeq 2000 P3 Reagents (200 Cycles) were used from Illumina. The libraries were combined with 40% PhiX (Illumina). Sequencing primers were used as described by Charbonnaeu and coworker (Charbonneau et al., 2017). The amplified libraries were single end sequenced using the Illumina NextSeq at the Genome Competence Centre of the Robert Koch Institute.

### 2.9 TraDIS analysis

For TraDIS analyses, genome sequences from the *S. canis* library used for infection analyses are named “input pool” and sequences from bacteria recovered after cell culture infections are named “output pool”. From input pool and from all output pools, raw fastq files were analyzed using the Bio-TraDIS (v1.4.5) scripts of the Sanger Welcome Trust Institute (https://github.com/sanger-pathogens/Bio-Tradis) in a similar way as described by Charbonneau and coworker (Barquist et al., 2016; Charbonneau et al., 2017). First, contigs were trimmed to 68 bp using the Trimmomatic read trimming tool (v0.40) (Bolger et al., 2014). Next, the bacteria_tradis pipeline script filtered reads according to the specified transposon tag (GAGAAAACTTTGCAACAGAACC). After tag removal, the remaining 46 bp of *S. canis* DNA were mapped to the IMT49926 reference genome (NCBI Bioproject: PRJNA945807) using SMALT short read mapper (v0.7.6) (http://www.sanger.ac.uk/resources/software/smalt/), producing a plot file of insertion sites for downstream analysis. Insertion sites were plotted on the reference genome using DNAplotter in the Artemis genome browser (v18.2.0) (Rutherford et al., 2000; Carver et al., 2009). A transposon tag mismatch of 1 was allowed, while the mapping threshold was set to 100%. Next, the command tradis_gene_insert_sites was used to create a document of unique insertion sites, total read counts and insertion indices. The output file from tradis_gene_insert_sites was used in tradis_essentiality to determine the essential genome of *S. canis* strain IMT49926. Lastly, the tradis_comparison.R script was used to compare the input (control) and output pools (conditions) using tradis_gene_insert_sites output files from each experimental replicate (n=3) of the pools. This generates a volcano plot illustrating log 2-fold change and -log2 (q-values) for every mutagenized gene as well as a csv file summarizing this information. The csv files were used for further downstream analysis in R.

The IMT49926 genome was functionally annotated using the eggNOG-mapper 2.1.12 (Cantalapiedra et al., 2021). COG annotations were subsequently added to the csv output files from the TraDIS analysis. To visualize the data, the csv file was analyzed with R (v4.4.0). A subset of hits made of the genes with logFC>2 and q value<0.01 was selected. This subset was used to create a heatmap with pheatmap (v1.0.12) (Kolde, 2015). A second subset was designed to compile only genes with an LPXTG-anchoring domain, which are characterizing surface proteins of Gram-positive bacteria. Analogously, a heatmap was created from this subset.

### 2.10 Detection of *sof* presence and alignment of *sof* genes

For sequence alignment, genomes of isolates were taken directly from a previous study where IMT49926 was classified as part of the BAPS-1 cluster (Bioproject PRJNA1332248) (Aubry et al., 2025). A comparison to the BAPS-2, BAPS-5, and BAPS-6 clusters was conducted to ascertain whether the *sof* gene was lineage specific or universally present. Assembly of the isolates was conducted with the shovill v1.1.0 (https://github.com/tseemann/shovill) pipeline under default settings. Annotations for the isolates were generated with bakta v1.11.4 (Schwengers et al., 2021). Detection of the *sof* gene presence was determined as present or absent via abricate v1.2.0 (https://github.com/tseemann/abricate) with 70% minimum sequence identity and query coverage of a user generated database containing the IMT49926 *sof* sequence as a reference. Alignment of the detected *sof* genes was conducted with mafft v7.471 under default settings (Katoh et al., 2019). Panaroo v1.6.0 was used under default settings to determine the core genome of the 64 isolates (Tonkin-Hill et al., 2020). The alignment of the core genome was conducted with mafft v7.471 under default settings (Katoh et al., 2019). Recombination was detected and removed from the core genome alignments via gubbins v3.4.1 under default settings (Croucher et al., 2015). RAxML v.8 was used to generate the Maximum Likelihood tree from the alignments with 1000 bootstrap replicates (Stamatakis, 2014). Tree and metadata are publicly available on https://microreact.org/project/ucbYDSmf6n5zwUyJxtQ24a-miriamsof.

### 2.11 Electron microscopy

For imaging of liquid bacterial samples, *S. canis* IMT49926 and 49926Δ*sof* were grown to an optical density (OD_600nm_) of 0.4 before being fixed. The fixative solution contained (per sample): 2880 μL cell culture medium, 800 μL paraformaldehyde (25%), and 320 μL glutaraldehyde (25%). Sample preparation and imaging for scanning electron microscopy (SEM) and transmission electron microscopy TEM were performed at the Central Facility for Microscopy (ZEIM) at the Helmholtz Centre for Infection Research and performed as described before (Kopenhagen et al., 2022; Deschner et al., 2025).

### 2.12 Fibronectin binding by FACS

*S. canis* IMT49926 and 49926Δ*sof* were grown to mid-exponential phase in THB medium. Cells were washed once in PBS and adjusted to 1*10^8^ CFU/mL. A total of 2*10^7^ CFU was suspended in 100 µL PBS with 0.5% BSA. Fibronectin-FITC or Fibrinogen-FITC (positive control) was added to obtain a final concentration of 50 µg/mL. Bacteria were incubated in the dark at 37 °C for 30 minutes. Bacteria were then washed in PBS and fixed in 4% paraformaldehyde in PBS. A total of 10^6^ bacteria were analyzed by flow cytometry as described before (Lapschies et al., 2023).

### 2.13 Fibronectin binding on glass coverslips

In a 24-well plate, 12 mm glass coverslips were inserted and coated with 50 µg/mL of human recombinant fibronectin. After washing with PBS, a bacterial culture of either *S. canis* IMT49926 or 49926Δ*sof* with OD 0.05 in THB was added to the wells. After the indicated incubation time, unbound bacteria were aspirated and wells were washed at least three times with PBS, after which they were fixed with 4% paraformaldehyde in PBS for 30 minutes. Bacteria were stained with polyclonal anti-*S. canis* and secondary Alexa fluor 488 antibodies. After embedding, images were obtained using a DMI6000B fluorescence microscope equipped with a 63x/1.40 oil immersion objective and operated with LAS X software (Leica, Germany). The proportion of the surface area covered by bacteria was quantified using ImageJ by calculating the percentage of the image exhibiting green fluorescence in three independent experiments with three technical replicates each.

### 2.14 Serum opacification activity

To assess the opacity reaction of streptococcal SOF, the ability to turn serum turbid by an interaction between SOF and high-density lipoproteins, Courtney and Pownall described assays for detecting and measuring the opacity reaction using horse serum (Courtney and Pownall, 2010). For excreted SOF and SOF that is covalently linked to the cell wall, *S. canis* IMT49926 and 49926Δ*sof* were grown to mid-exponential phase in 10 mL THB and centrifuged (5000 *xg*, 5 min) to separate supernatant from the cell pellet. The cell pellet was resuspended in 1 mL of sterile PBS. Both samples, supernatant and cell suspension, were heat-treated at 65 °C for 20 min to stop bacterial growth. Afterwards, 10 µL sample were added to 100 µL of horse serum in a 96-well plate format. After overnight incubation at 37 °C, 100 µL of 0.85% sterile NaCl-solution was added and absorbance measured at 450 nm. For non-covalently associated SOF, 1% SDS extracts of overnight bacterial cultures were prepared as described by Courtney and Pownall (Courtney and Pownall, 2010). 25 µL of SDS extract was added to 100 µL of horse serum in a 96-well plate and incubated at 37 °C overnight before absorbance was measured as mentioned above. Strain *S. suis* M10 was used as a positive control, while horse serum alone acted as a negative control.

### 2.15 Statistical analysis

All experiments were performed in three independent biological assays, each with at least three technical replicates. Results are presented as mean values ± standard deviation (SD). In general, graphs and statistical calculations were generated using the Prism software (v8.0) (GraphPad). Unpaired, two-tailed student t tests were performed unless specified otherwise in the figure captions. * Indicates p < 0.05, ** indicates *p* < 0.01, *** indicates *p* < 0.001 and **** indicates p < 0.0001. The proportion of proliferating versus migrating cells was quantified by counting red- and blue-fluorescing cell nuclei within the defined gap area at the indicated time points (6 h, 24 h, and 48 h). For each coverslip, a minimum of four randomly selected fields of view of the wound were imaged and used for nuclear quantification. Data are expressed as mean values with standard deviation. * Indicates p < 0.05, **) indicates p < 0.01, and ***) indicates p < 0.001, according to one-way ANOVA and Tukey HSD *post hoc* tests. For the 24 h samples homoscedasticity was not met (Levene p < 0.05); therefore, Welch’s ANOVA and Games-Howell *post hoc* testing were applied.

## 3 Results

### 3.1 Identification of novel *S. canis* IMT49926 genes involved in adherence and invasion of endothelial cells

**Figure 1:**
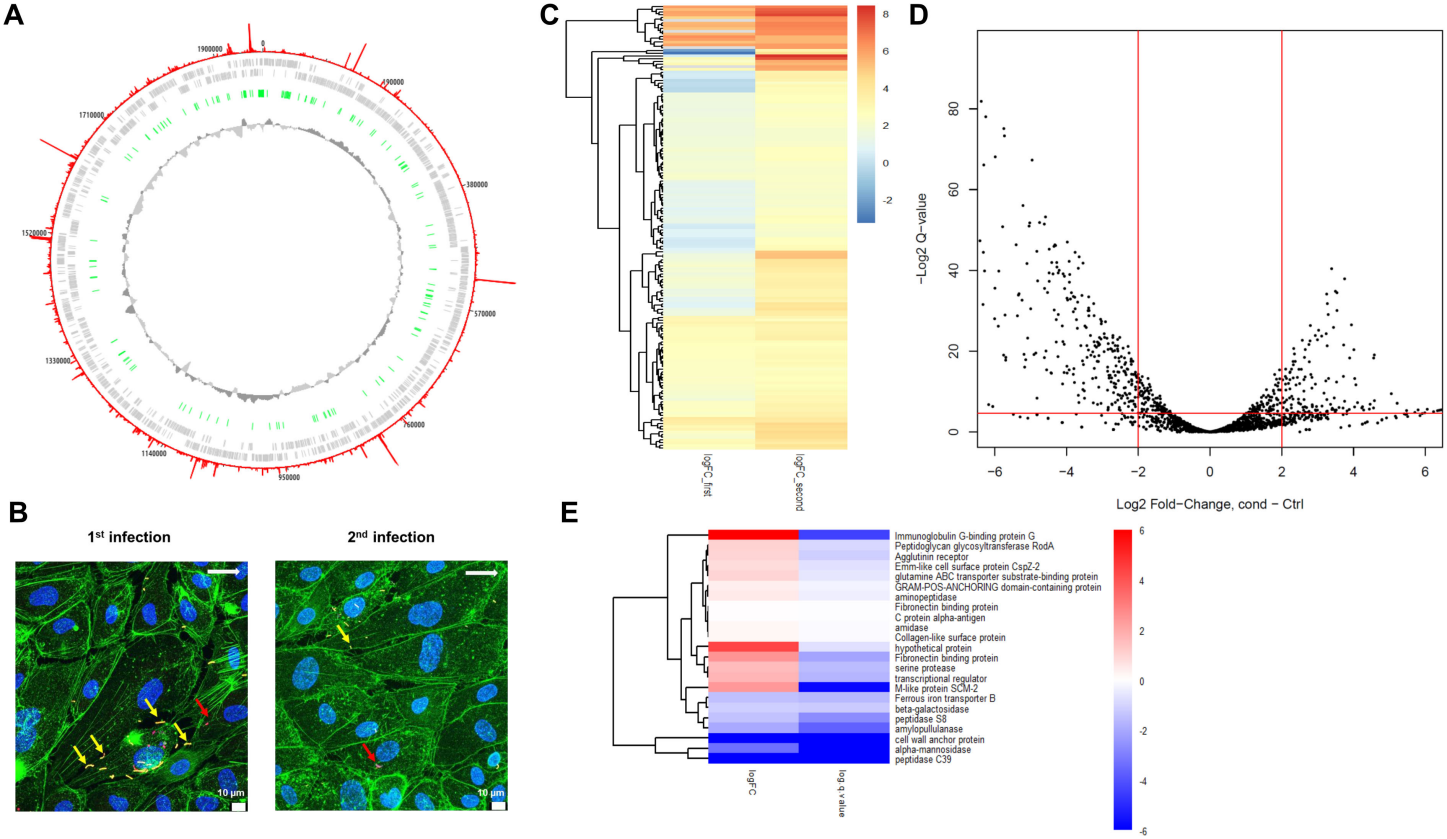
Identification of S. canis genes involved in adherence and invasion of endothelial cells. **(A)** Circular genome representation of IMT49926. Transposon insertion frequency is represented in red, gene annotations in grey. In green, the essential genes are depicted, while the inner track illustrates the GC content. Image was created with DNAplotter. **(B)** Representative DIF-stained CLSM image of HUVECs grown under flow conditions and infected with the input pool (1^st^ infection) and with the output pool of the first infection round, showing a decrease in adherence (2^nd^ infection). Adherent bacteria are highlighted in yellow (yellow arrows). Internalized bacteria are indicated in red (red arrows). HUVEC actin cytoskeleton is marked in green and cell nuclei in blue; the direction of flow is marked with a white arrow, scale bar indicates 10 µm. **(C)** Heatmap of all overrepresented, mutagenized genes (q <0.01) in the output pool after the second selection (right column) with corresponding logFC of these genes after the first round of infection (left column). Genes are clustered according to their profiles in all samples (dendrogram, left side). **(D)** Volcano plot of a comparative analysis of the generated output pool (non-adherent bacteria after second selection round) and the input library showing the logFC of all genes after two rounds of infection compared to the input pool (control). **(E)** Heatmap of all LPXTG-anchoring domain containing genes with the logFC in the left column and log 2 q-value in the right column.

The clinical *S. canis* strain IMT49926 was isolated from a case of infective endocarditis (Katsburg et al., 2023) and was used for the generation of a genomic transposon library. Library saturation and insertion density is plotted in **Figure 1A**, showing genome-wide coverage of the transposon insertions. The mutant library comprised 21879 unique insertion sites across the genome. Of 1914 annotated genes, 1727 (90.2%) contained at least one transposon insertion with an average of 9.7 insertions per gene and a median of 6 insertions per gene. A total of 187 genes showed no insertions. Using Artemis for visualization, transposon insertion into every gene was manually confirmed (**Supplementary Figure 1**). The library was used to infect a cell culture under defined flow conditions and thus, identify transposon mutants with reduced cell adherence ability. For this purpose, two rounds of infection were subsequently performed providing a first and a second set of separately harvested adherent and non-adherent bacteria as output pools. Confirmation of the successful separation of adherent and non-adherent transposon mutants is provided by immunofluorescence microscopy after infection with the input library (first set) and with the non-adherent output pool (second set) as shown in **Figure 1B**. The immunofluorescence overlay visualizes a reduced number of adherent bacteria after the 2^nd^ round of infection compared to the first (**Figure 1B**, yellow arrows). After the first infection round, only limited enrichment of adherence-defective mutants was observed (**Supplementary Figure 2**). Therefore, a second round of infection was performed using the non-adherent output pool to increase enrichment stringency. The second selection round resulted in clear enrichment of mutants defective in endothelial association, enabling identification of candidate genes involved in adhesion and invasion (**Figures 1C, D**; **Supplementary Tables 1**, **S2**). A positive logFC indicates enrichment of transposon mutants in the non-adherent output pool relative to the input library, suggesting that disruption of these genes reduces bacterial adherence or invasion capacity. In contrast, negative logFC values indicate depletion from the non-adherent fraction and therefore are less likely to contribute to endothelial association defects under the tested conditions. To prioritize robust candidate genes while minimizing false positives, only genes with logFC > 2 and q < 0.01 were considered for downstream analyses. The full list of these 94 genes that are shown in the heat map in **Figure 1C** is found in **Supplementary Table S2**.

Because LPXTG-anchored proteins are surface-exposed factors frequently involved in host interaction and adhesion in Gram-positive bacteria, this protein class was prioritized for functional validation. In sum, 23 genes were identified carrying the classical coding sequence for an LPxTG-mediated cell wall anchorage of which 3 genes remained with the criteria (logFC > 2 and q < 0.01) likely encoding proteins relevant for the endothelial infection under these experimental settings as shown in **Figure 1E** and **Supplementary Table S3**. Based on functional domain homologies and on gene annotations, these 3 genes are identified as an IgG-binding protein G homologue, an annotated fibronectin binding protein, and the M-like protein SCM type II. Since fibronectin-binding proteins are identified as virulence factors in IE pathogenicity for other streptococcal species, we selected this gene for further functional investigation (Que et al., 2005; Jung et al., 2009). By comparing the gene using the NCBI BLAST core_nt database, this gene showed high similarities (>90%) with serum opacity factors of other streptococcal species. We will refer to the gene in the rest of this manuscript as *sof* and the corresponding protein as ScSOF, the serum opacity factor of *S. canis*. To test the functional characteristics of ScSOF, an isogenic deletion mutant of the *sof* gene was constructed by targeted mutagenesis. The mutant showed no additional mutations or difference in growth kinetics (**Supplementary Figure 3**).

### 3.2 ScSOF is a serum opacity factor that is covalently and non-covalently associated with the cell surface and plays a role in the inhibition of ß-hemolysis

**Figure 2:**
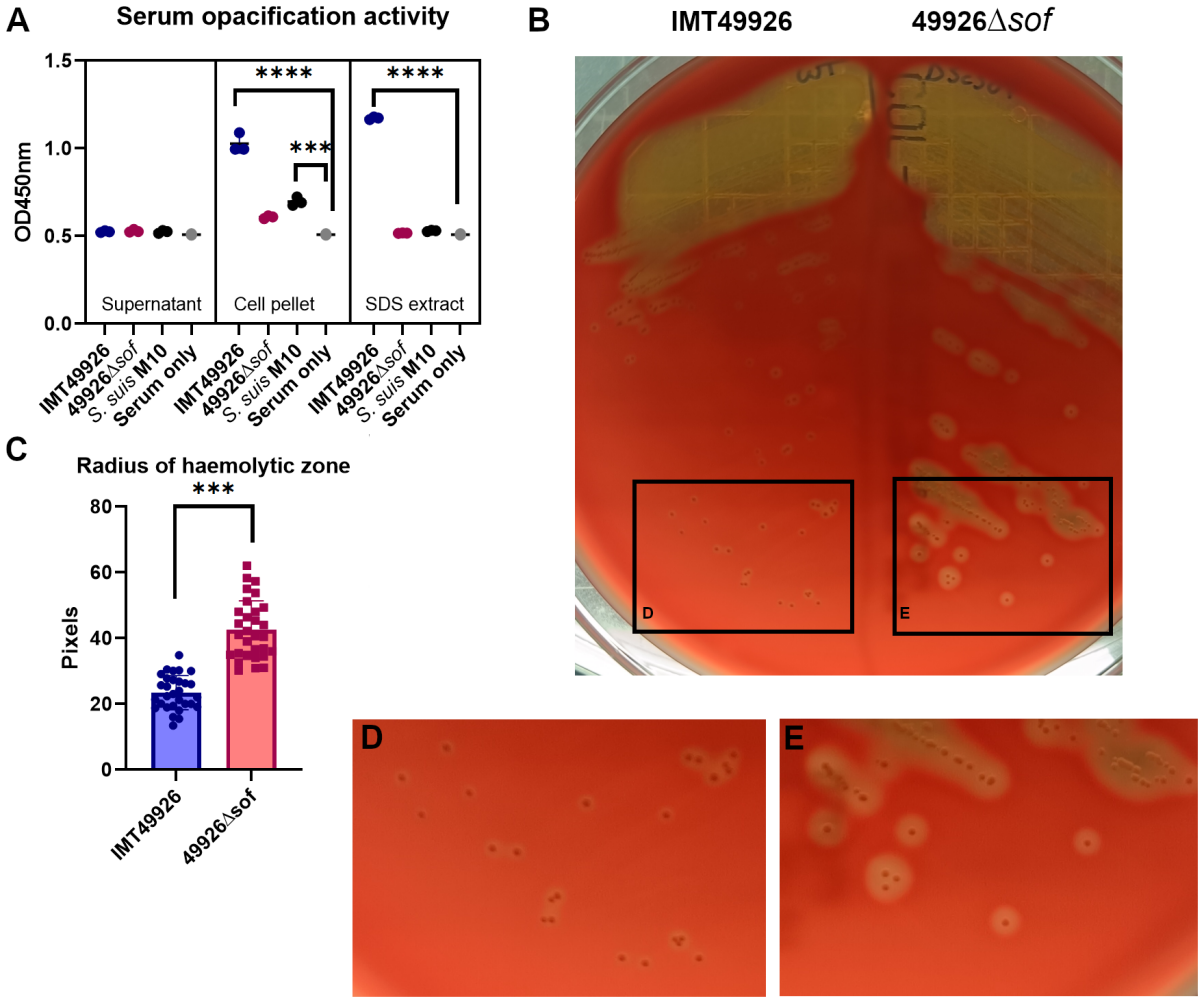
ScSOF opacifies serum and inhibits ß-hemolysis. **(A)** Serum opacification activity of strain S. canis IMT49926 and 49926Δsof, S. suis M10 and a negative serum control. Showing (left to right): heat-inactivated bacterial supernatant, bacterial sediment suspension from logarithmic growing bacteria and 1% SDS extracts of overnight cultures. **(B)** Representative picture of a blood agar plate with colonies of S. canis IMT49926 (left) and 49926Δsof (right). **(C)** Radius of the hemolytic zone measured with ImageJ based on pictures of blood agar plates of 3 biological replicates comparing IMT49926 and 49926Δsof every time on the same plate. Statistical significance was calculated by Two-way ANOVA. *** indicates p < 0.001 and **** indicates p < 0.0001. **(D)** Zoom-in of single colonies on blood agar plate of S. canis IMT49926 as used for quantification in ImageJ. **(E)** Zoom-in of single colonies on blood agar plate of S. canis 49926Δsof as used for quantification in ImageJ.

BLAST analysis showed high gene sequence similarities (around 80%) of ScSOF with the LPxTG-motif carrying serum opacity factor of *Streptococcus dysgalactiae* and around 50% protein sequence similarity with the serum opacity factors of *S. dysgalactiae* and *S. pyogenes.* These proteins show serum opacification activity and play a role in fibronectin binding (Courtney et al., 1999; Katerov et al., 2000; Oehmcke et al., 2004; Courtney et al., 2009; Courtney and Pownall, 2010; Zhu et al., 2017). An alignment of the conserved fibronectin-binding parts of the protein is shown in **Supplementary Figure 4**, as well as the results from the BLAST searches. Serum opacification activity in streptococci is caused by binding of serum opacity factors to high-density lipoproteins (HDL), displacing apolipoprotein A-I and disrupting the structure of HDL. This results in formation of large particles generating serum turbidity (Courtney et al., 2006). We tested the ability of the ScSOF to opacify serum using these methods. No serum opacification activity was detected upon incubation of horse serum with bacterial supernatant, indicating that ScSOF protein is not excreted into the extracellular milieu of *S. canis* during logarithmic growth (**Figure 2A**). However, we observe opacification activity during logarithmic growth caused by the bacterial pellet of the wild type strain IMT49926 but not the *sof* deletion mutant, indicating that active ScSOF is predominantly associated with the bacterial surface. Additionally, to test for the presence of non-covalently bound SOF, we used SDS extracts of an overnight culture, where we detect opacification activity in the wild type strain but not in the deletion mutant, indicating that ScSOF is non-covalently associated with the surface in the stationary growth phase. The absence of opacification activity in all mutant-derived fractions confirms that ScSOF is the major determinant responsible for serum opacity activity in strain IMT49926. **Figures 2B–E** show the zone of ß-hemolysis of IMT49926 and 49926Δ*sof* on a blood agar plate which is increased in the *sof* deletion mutant indicating that ScSOF could play a role in inhibition of ß-hemolysis.

**Figure 3:**
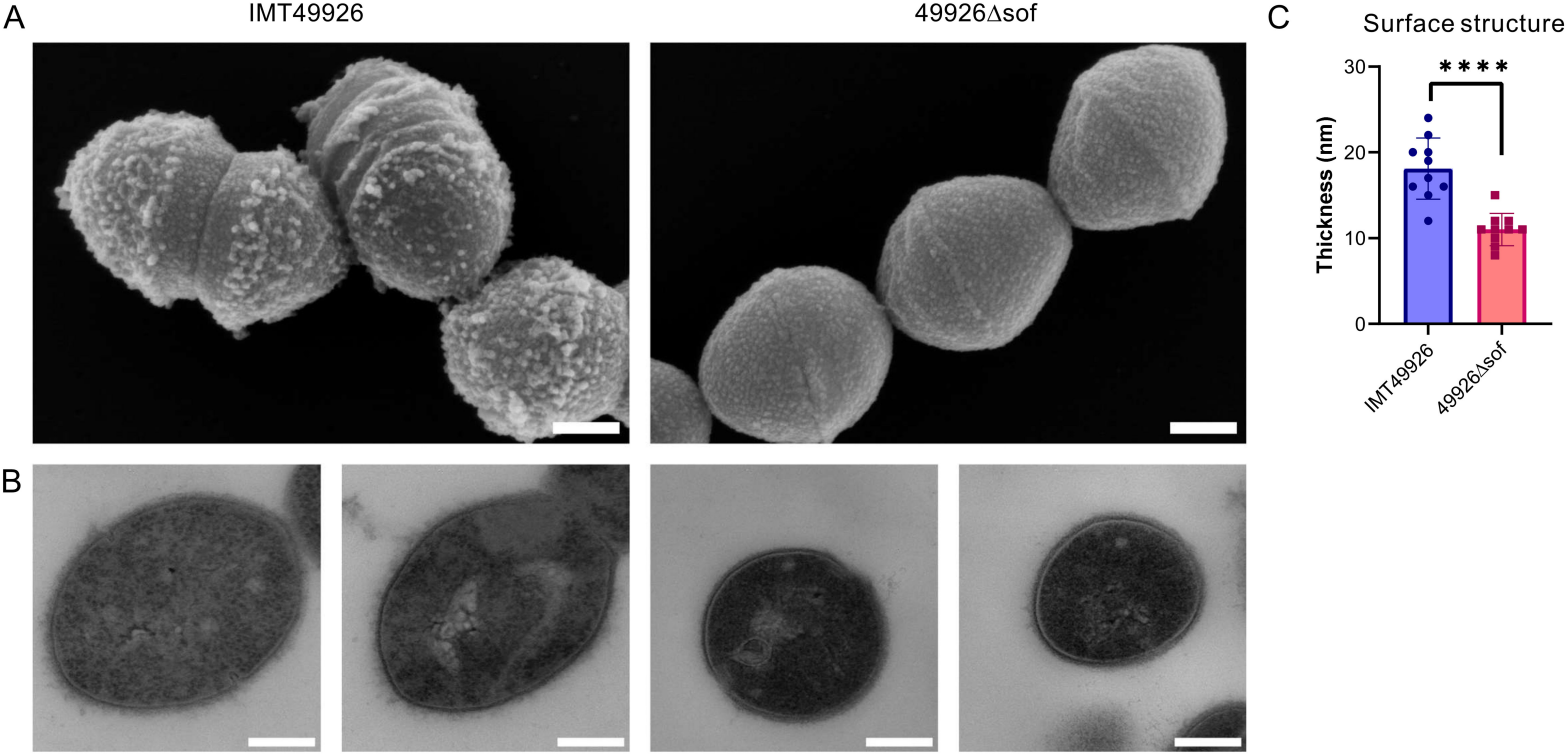
ScSOF is a surface protein of S. canis. **(A)** SEM visualization of IMT49926 in the left panel and of the 49926Δsof mutant in the right panel. **(B)** TEM images of IMT49926 and the 49926Δsof mutant. Scale bars indicate 200 nm. **(C)** Quantification of surface structure thickness of IMT49926 and 49926Δsof. Statistical significance was calculated by Welch’s Students t-test. **** indicates p < 0.0001.

To visualize potential ScSOF-mediated differences in bacterial surface structures of *S. canis*, we used scanning electron microscopy to compare the wild type strain IMT49926 with the mutant strain 49926Δ*sof S. canis* IMT49926 exhibits a notable surface layer that appears rough and densely decorated (**Figure 3A**). The deletion mutant 49926Δ*sof* has a smoother, less textured surface, indicating loss of surface structures. To visualize the surface architecture of the wild type and deletion mutant at a higher resolution, cross-sectional images were acquired using a transmission electron microscopy as displayed in **Figure 3B**. A series of scanning electron and transmission electron microscopic images is added as **Supplementary Figure 6**. Again, there is a notable difference in the thickness of the bacterial surface structures, displaying markedly decreased thickness for the mutant as confirmed by measurements of the surface material (mean surface thickness) shown in **Figure 3C**.

### 3.3 ScSOF contributes to fibronectin-binding of *S. canis*

**Figure 4:**
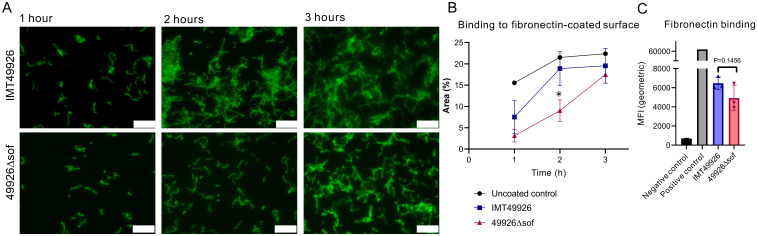
ScSOF plays a role in fibronectin-binding. **(A)** S. canis IMT49926 and 49926Δsof (green) on a fibronectin-coated coverslip fixed at different time points. Images are acquired with a DMI6000B fluorescence microscope. Scale bars indicate 10 µm. **(B)** Area covered with bacteria (determined by ImageJ analysis of images of three independent experiments. **(C)** FACS analysis of the direct fibronectin-binding ability of IMT49926 and 49926Δsof using FITC-conjugated fibronectin. Data represents geometric mean fluorescence intensity (MFI) ± SD of three independent experiments.

Domain homology comparisons identified three fibronectin-binding repeats in the protein sequence of ScSOF, pointing to a further important characteristic of ScSOF as a fibronectin-binding protein. To analyze the impact of ScSOF in fibronectin binding of *S. canis*, the wild type strain and the isogenic ScSOF deletion mutant were used in two functional assays. First, immunofluorescence was used to visualize bacteria bound to a fibronectin-coated coverslip at different time points as shown in **Figure 4A**. After 2 hours, the *sof*-deletion mutant covers a substantially smaller fibronectin-coated area than the wild type. However, after 3 hours, this difference is no longer visible. This may indicate that ScSOF primarily contributes to early adhesion events, while prolonged incubation allows compensatory binding by additional surface-associated factors or accumulation of nonspecific bacterial attachment. The quantification of fluorescence signals confirms the significant reduction of fibronectin binding by the *sof*-deletion mutant after 2 hours (9.03% ± 2.52%, p = 0.0218)) compared to the wild type (18.9% ± 3.95%) (**Figure 4B**). Second, binding of FITC-labelled recombinant fibronectin to wild type and isogenic *sof*-deletion mutant was detected by FACS analyses. The geometric mean fluorescence intensity confirmed a slightly higher binding activity of wild type bacteria to FITC-labelled fibronectin than of the *sof*-deletion mutant (**Figure 4C**).

### 3.4 Confirmation of the attenuated TraDIS mutant phenotype by the isogenic deletion mutant of the surface protein ScSOF

**Figure 5:**
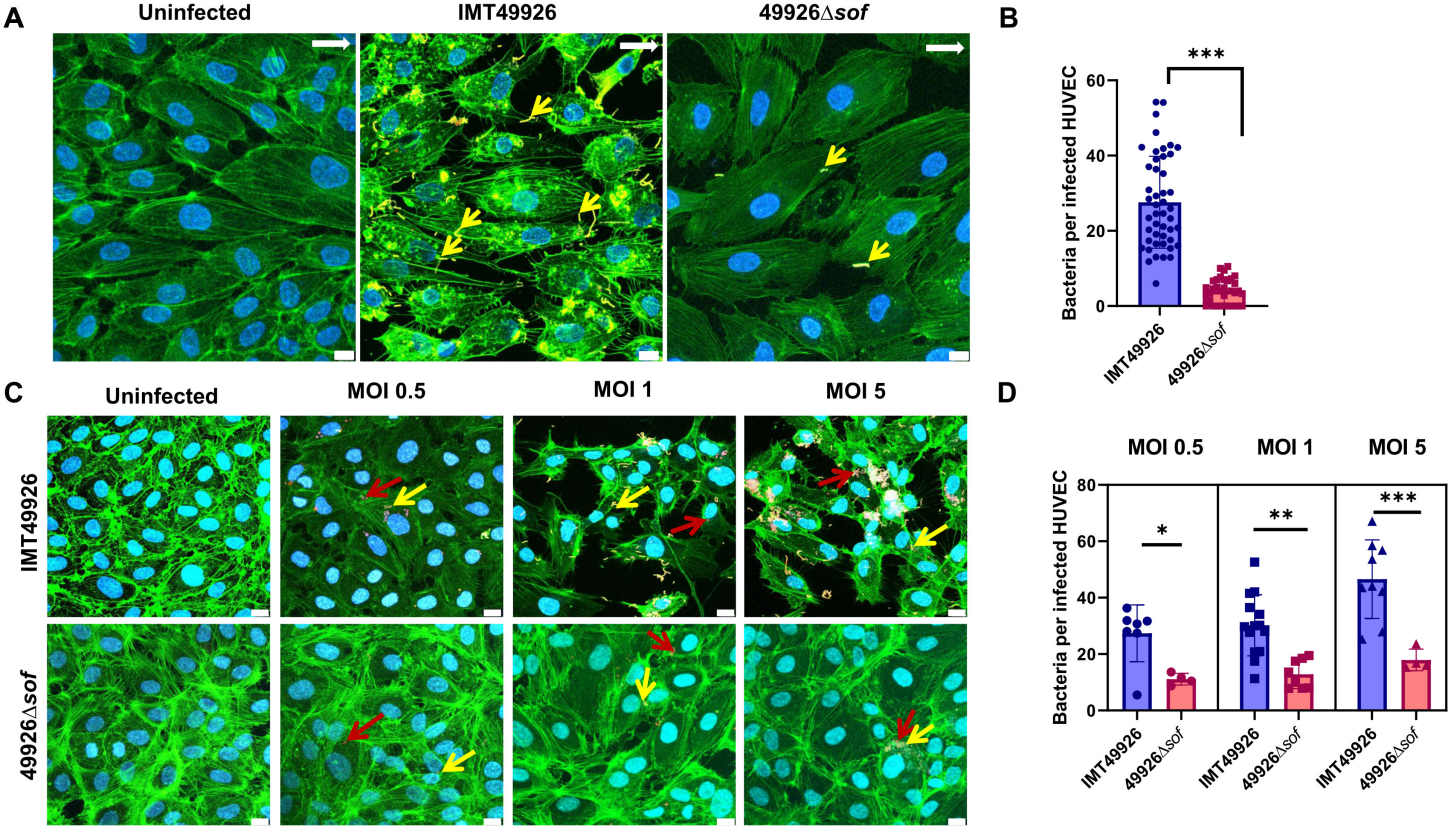
Deletion of the sof gene leads to a decrease in adherence and invasion of HUVECs. **(A)** Representative images of HUVEC infection with IMT49926 and the 49926Δsof mutant at MOI of 10 and shear stress of 6 dyn/cm² for up to 2.5 h (white arrows indicate direction of flow). Actin cytoskeleton (green), nuclei (blue), adherent streptococci (yellow) and internalized bacteria (red). Scale bars represent 10 µm. **(B)** Quantification of cell-attached bacteria after infection under microfluidic conditions. **(C)** HUVEC monolayers were infected with IMT49926 and the 49926Δsof mutant at MOI 0.5, 1 and 5 under static conditions. Staining and imaging as above. Scale bars represent 10 µm. **(C)** Quantification of bacterial adherence to HUVEC at different MOIs (0.5, 1, and 5) under static conditions. * Indicates p < 0.05, ** indicates p < 0.01, *** indicates p < 0.001.

To confirm the attenuated phenotype of the transposon mutant, the isogenic deletion mutant was compared to the IMT49926 wild type strain in the endothelial infection of HUVEC cells under flow conditions that was used to generate the TraDIS output pools. Wildtype infection resulted in abundant endothelial association and cytoskeletal remodeling, whereas the *sof* deletion mutant displayed markedly reduced bacterial interaction and limited cytoskeletal disruption. In the left panel of **Figure 5A**, uninfected HUVEC cells under flow conditions mimicking vascular shear stress are shown. The uninfected cells exhibit intact stress fibers and a tight cytoskeletal organization. In the middle panel, the wild type infected cells show cytoskeletal remodeling including loss of stress fibers and formation of membrane protrusions and intracellular actin aggregates, with abundant bacterial association (yellow arrows). In the right panel, significantly reduced bacterial adherence and limited cytoskeletal perturbation is visualized after infection with the deletion mutant, comparable to the uninfected control. A quantification of the cell-associated bacteria also confirms a decrease in bacterial adherence to the endothelial cells (**Figure 5B**).

To determine whether this phenotype was dependent on flow-induced host responses, additional infections were performed under static conditions using increasing MOIs (0.5 – 5). Wildtype bacteria induced progressive cytoskeletal disruption and bacterial aggregation in a dose-dependent manner, whereas the *sof* deletion mutant exhibited strongly reduced adherence and invasion across all MOIs tested (**Figure 5C**). Quantitative analyses confirmed significantly decreased endothelial association of the mutant strain in comparison to the wild type (**Figure 5D**). Together, these findings demonstrate that ScSOF contributes substantially to endothelial interaction under both flow and static conditions. A similar decrease in bacterial adherence and invasion was detected upon infection of A549 lung epithelial cells (**Supplementary Figure 5**), further demonstrating the importance of ScSOF in the pathogenicity of *S. canis*.

### 3.5 ScSOF is involved in wound healing of endothelial cells

**Figure 6:**
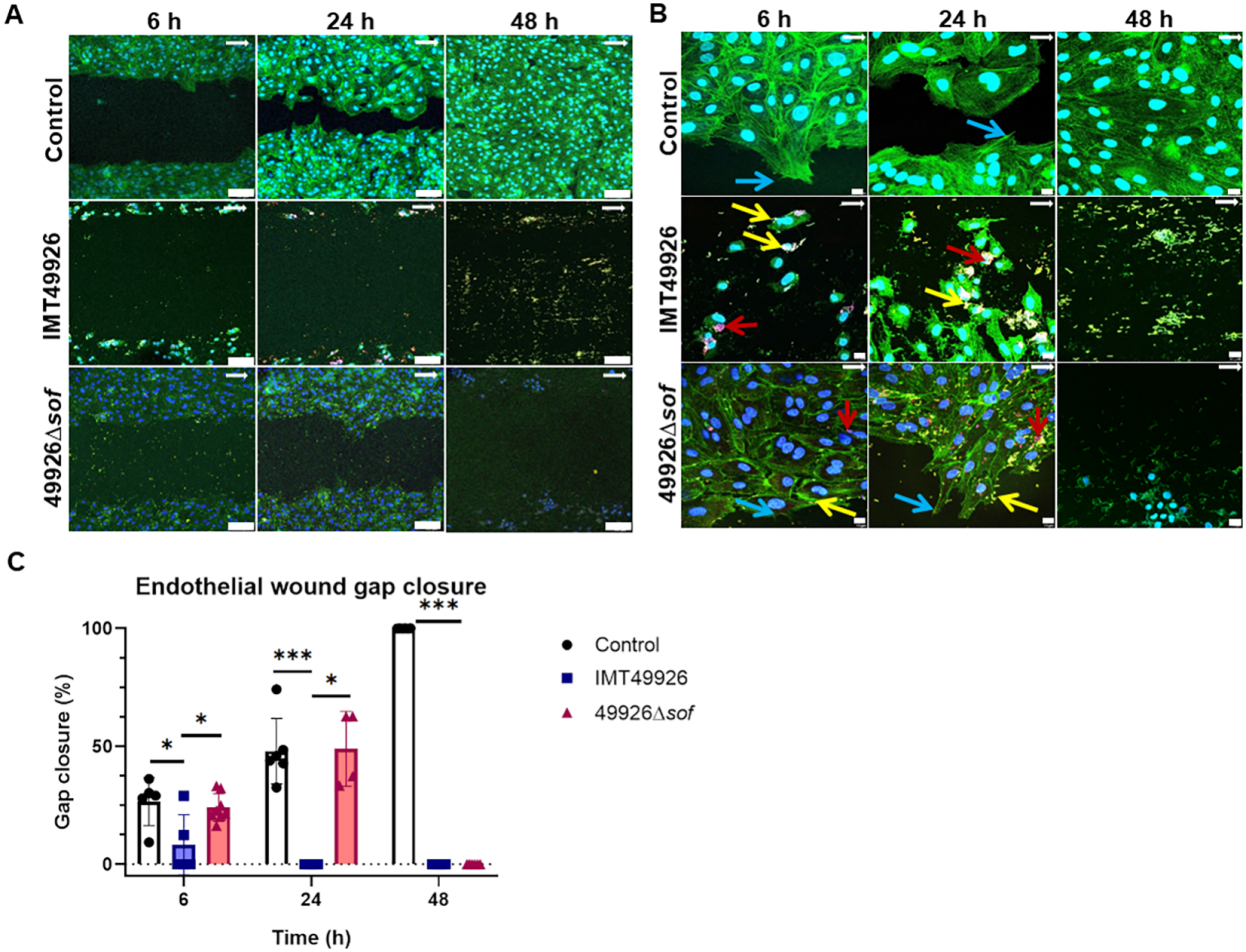
ScSOF plays a role in endothelial wound healing. **(A)** Endothelial cell morphology was visualized by DIF staining at time points 6 h, 24 h and 48 h. Representative overlay images are shown for each time point of the CSMA without bacteria (ctrl), and in the presence of wild type S. canis or 49926Δsof. White arrows mark the direction of flow. Scale bar represents 100 μm. **(B)** Representative microscopic images of CSMA results after DIF-staining at 630-fold magnification. Blue arrows point to cell evaginations at the growing cell borders (ctrl). Yellow arrows point to adherent S. canis, and red arrows point to internalized bacteria. White arrows mark the direction of flow. Images generated using CLSM (Leica Sp8) at 630-fold magnification and a zoom factor of 0.75. Scale bar represents 10 μm. **(C)** At indicated time points, progress of gap closure was calculated in percent in relation to the starting point, which is defined as 0% gap closure. Values are expressed as mean values with standard deviation. * Indicates p < 0.05, and *** indicates p < 0.001, according to one-way ANOVA and Tukey HSD *post hoc* tests. For the 24 h and 48 h samples homoscedasticity was not met (Levene p < 0.05); therefore, Welch’s ANOVA and Games-Howell *post hoc* testing were applied.

Septic bloodstream infections and infective endocarditis are linked to endothelial cell damage. Using the Chamber-Separation cell Migration Assay (CSMA), HUVECs were exposed to increasing shear stress (3 to 10 dyn/cm² over 2 h), and then infected with *S. canis* (**Figures 6A, B**). Immune fluorescent imaging of wound regeneration after staining of HUVEC cytoskeleton in green and cell nuclei with DAPI in blue clearly demonstrated that in uninfected controls, the cell-free gap of two adjacent HUVEC layer closed progressively over time, reaching nearly complete closure by 48 h (**Figures 6A**, control). Infection of HUVEC with *S. canis* wild type IMT49926 markedly impairs endothelial cell wound closure under physiological shear stress (**Figure 6A**), even enhancing the size of the cell-free gap area (**Figure 6A**). **Figure 6B** depicts magnifications of specific infection sites during CSMA with wild type *S. canis* bacteria and arrows point to bacteria attached (yellow) or internalized into HUVEC (red arrows). Comparison of infected HUVEC with the uninfected control visualizes morphological changes typically indicating cell damage already after 6 h of infection with wild type bacteria (**Figure 6B**). In contrast, incubation with the *sof* deletion mutant partially rescues regenerative capacity at 24 hours, although this protective effect is no longer sustained at 48 hours (**Figures 6A, B**).

In addition to microscopic imaging, endothelial wound gap closure was also quantified by measurement of gap sizes as depicted in **Figure 6C**. Resulting data confirmed visual observation. Detailed gap closure values for the uninfected control were: 26.5% ± 9.1% at 6 h, 48% ± 12.7% at 24 h, and 99.98% ± 0.01% at 48 h. For wild type-infected cells, closure was 8.3% ± 11.4% at 6 h and 0% at both 24 h and 48 h (**Figure 6C**). Only a partial cell regeneration was observed during infection of HUVEC with the *sof* deletion mutant, resulting in 24.2% ± 5.3% closure of the cell-free gap after 6 h and up to 49% ± 13.8% of gap closure after 24 h of cultivation. These values are comparable to the uninfected control, reaching 48% ± 12.7% gap closure after 24 h of cultivation (p = 0.99; **Figure 6C**). However, by 48 h of cultivation under infection with the deletion mutant, this restorative effect was no longer evident, and the gap area expanded similarly to wild type-infected cells. For *sof* deletion mutant -infected cells, closure was 24.2% ± 5.3% at 6 h and 49% ± 13.8% at 24 h. Statistical analysis confirmed significant differences between the uninfected control and wild type-infected cells at all time points (p < 0.05), as well as between wild type- and *sof* deletion mutant infected cells at 6 h and 24 h. At 48 h, only the uninfected control achieved complete gap closure, while both infected groups showed gap expansion due to cell loss.

### 3.6 *S. canis* inhibits cell proliferation and cell migration under physiological microfluidic conditions

**Figure 7:**
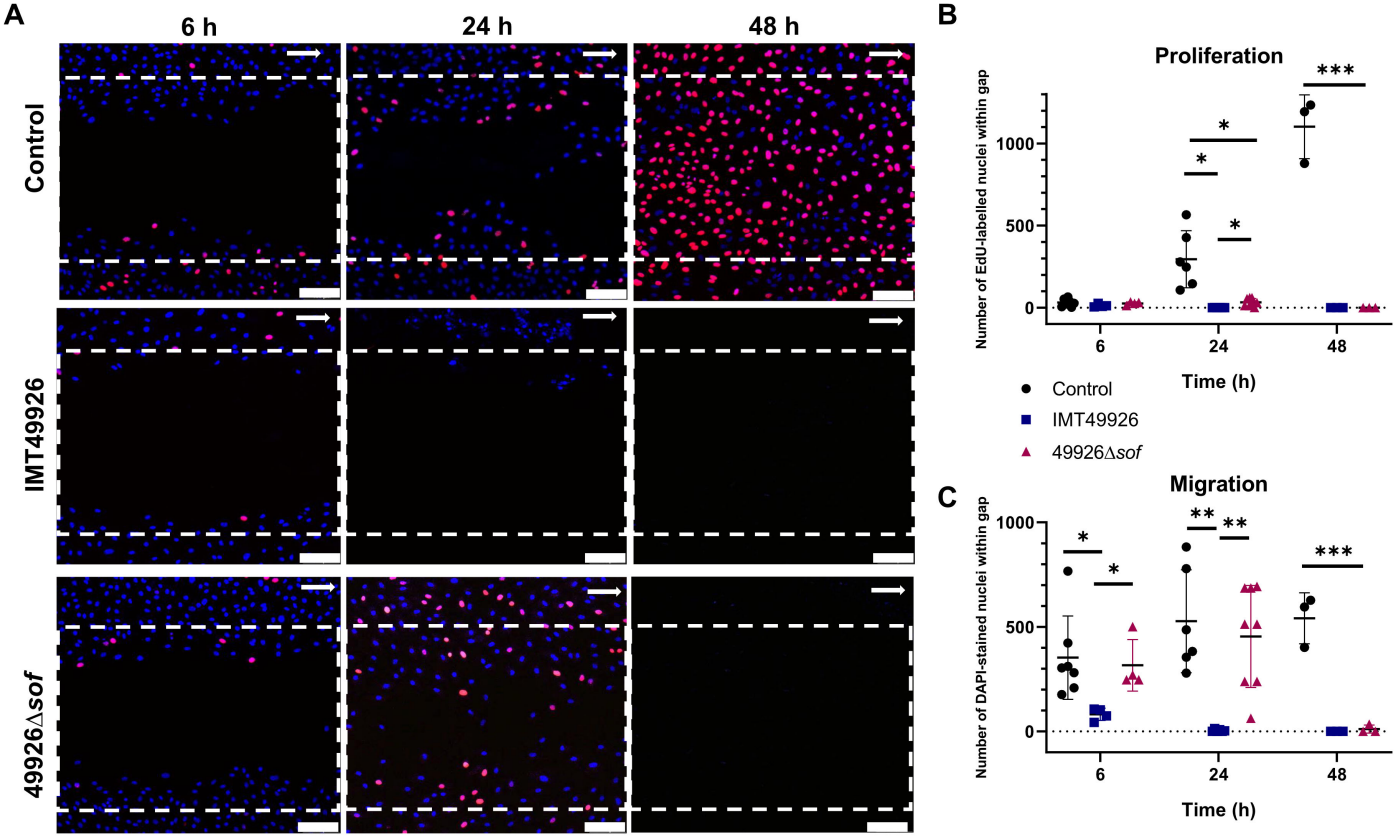
Differential microscopic evaluation of cell proliferation and cell migration with and without S. canis infection. **(A)** CSMA was performed for indicated time periods without bacteria (control), and after infection with IMT49926 and 49926Δsof at MOI 5 in the presence of EdU, which incorporates into proliferating HUVEC nuclei. In CSMA samples without bacteria, gap closure was completed after 48 h of cell cultivation. In the presence of bacteria, cell-free gaps were not closed after 48 h of cell cultivation. Representative microscopic fluorescence images are shown for each sample. Images were generated using Scale bar represents 100 μm. Proportion of proliferating HUVECs **(B)** and migrating cells **(C)** was quantified by counting the red and blue fluorescing cell nuclei in the defined gap area at indicated time points (6 h, 24 h and 48 h. Experiments were performed in three independent assays, each in triplicate, and a minimum of four different fields of view were randomly chosen for quantification of cell nuclei. Data are expressed as mean value including standard deviation. * Indicates p < 0.05, ** indicates p < 0.01, and *** indicates p < 0.001, according to one-way ANOVA and Tukey HSD *post hoc* tests. For the 24 h samples homoscedasticity was not met (Levene p < 0.05); therefore, Welch’s ANOVA and Games-Howell *post hoc* testing were applied.

Vascular shear stress acts as a key regulator of endothelial proliferation, migration, and barrier function. As demonstrated in **Figure 6**, closure of the cell free gap of uninfected HUVEC was achieved after 48 h of cultivation under microfluidic under shear stress of 10 dyn/cm². To determine how much of the gap closure was caused by cell proliferation versus how much by cell migration, nuclei of HUVEc cells were stained with DAPI for visualization of cell migration and additionally with EdU, which enables detection of red nuclei indicating proliferating cells (**Figure 7A**). Representative immunofluorescence images clearly visualize closure of the cell free gap of uninfected HUVEC after 48 h cultivation under flow by both migrating and proliferating HUVEC (**Figure 7A**). In contrast, no cell nuclei were detected after 48h in samples infected with wild type bacteria or with the deletion mutant.

Proportions of proliferating and migrating cells were quantitatively determined by quantifying red- and blue-fluorescent nuclei within the defined cell-free gap area at the indicated time points of cultivation (6 h, 24 h, and 48 h; **Figures 7B, C**). Experiments were conducted in three independent assays, each performed in triplicate, and at least four randomly selected fields of view were analyzed per wound for nuclear quantification. Data are presented as mean ± standard deviation. In uninfected samples, amount of red nuclei representing proliferating HUVEC cells increased from 30.9 ± 21.6 nuclei at 6 h to 1101.7 ± 159 nuclei at 48 h, while number of nuclei of migrating cells, which were stained in blue, rose from 352.9 ± 184.4 nuclei to 541 ± 99.4 nuclei (**Figures 7B, C**). After 48 h of HUVEC cultivations, when wound closure was complete, cell proliferation contributed more strongly to wound repair under flow conditions than cell migration. Infection with wild type *S. canis* significantly reduced cell proliferation, resulting in 12.3 ± 8.3 nuclei at 6 h; 0.33 ± 0.47 nuclei at 24 h; none at 48 h) and migration (82 ± 25.7 nuclei at 6 h; 4.33 ± 5.4 nuclei at 24 h; none at 48 h) compared to the uninfected control. Statistical significance based on one-way ANOVA followed by Tukey’s HSD *post hoc* test. For the 24 h time point, the assumption of homoscedasticity was violated (Levene’s test p < 0.05 p<0.05); therefore, Welch’s ANOVA with Games–Howell *post hoc* testing was applied. In presence of the *sof* deletion mutant, amount of cell proliferation and cell migration remained comparable to controls at time points of 6 h and of 24 h of cultivation, thereby indicating significant differences from wild type infection. These results demonstrate that *S. canis* wild type infection impairs both, endothelial cell proliferation and migration, during wound healing under shear stress.

### 3.7 Prevalence of the *sof* gene in *S. canis* population indicates association of *sof* with distinct evolutionary lineages

**Figure 8:**
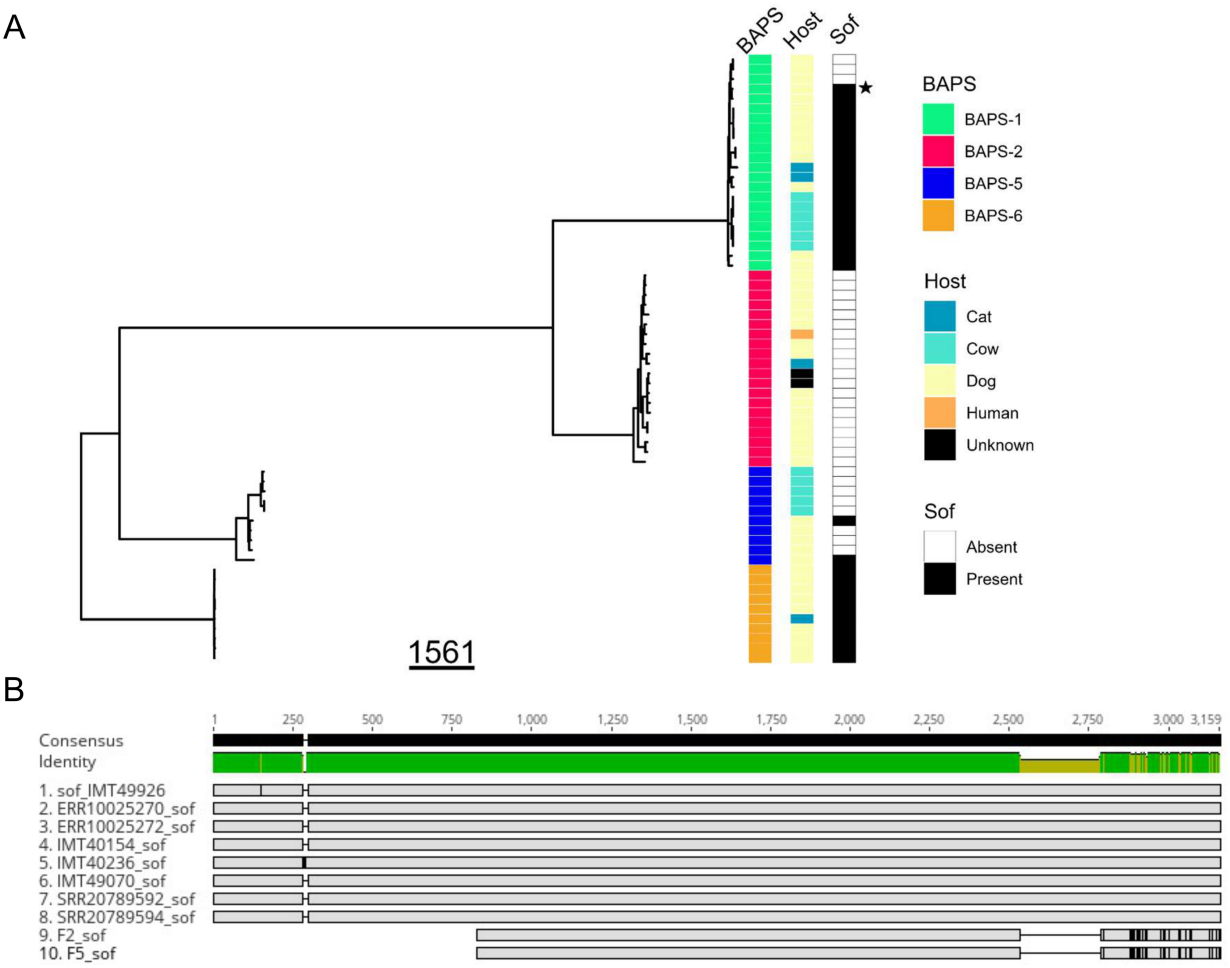
Analysis of the prevalence of the sof gene within a subpopulation of S. canis. **(A)** Maximum Likelihood core genome tree of 62 S. canis isolates represented by BAPS cluster, host origin, and the presence of the sof gene. Scale bar represents the number of SNPs based on the length of the tree branches. Black star represents the position of IMT49926 in the tree. **(B)** Sequence alignment of SOF encoding genes from S. canis strains in BAPS cluster 1, visualized with Geneious Prime 2025.2.2.

Based on the maximum likelihood tree of **Figure 8A**, the *sof* gene appears to be variably distributed and lineage-associated rather than uniformly present across the population. There is a clear clustering of *sof*-positive isolates within phylogenetic branches, which appears to correlate with the BAPS 1 and 6 clusters. These results suggest that *sof* prevalence is linked to specific lineages rather than randomly distributed. Since host origin varies within *sof*-positive and *sof*-negative groups, the gene does not appear to be strictly host-specific. The clustering of *sof*-positive isolates within specific phylogenetic lineages indicates a structured population distribution of the gene; however, the evolutionary mechanisms underlying this pattern remain unresolved. The *sof* gene is highly conserved across BAPS 1 cluster isolates over most of its length, as can be seen in **Figure 8B**, suggesting preservation of core functional domains alongside localized diversification.

## 4 Discussion

Despite increasing recognition of *S. canis* as a causative agent of infective endocarditis (IE) in both dogs and humans, the molecular mechanisms underlying its adherence to the host endothelium remain uncharacterized. While factors such as the M protein SCM have been implicated in immune evasion and early colonization, other specific adherence factors and their roles in endothelial interactions remain poorly understood (Pagnossin et al., 2022).

We employed a high-throughput TraDIS approach in combination with a physiologically relevant *in vitro* endothelial infection model that incorporates shear stress, to identify bacterial determinants contributing to endothelial interaction in *S. canis*. Using this approach, we identified ScSOF as a multifunctional virulence-associated factor involved in fibronectin binding, serum opacification, cytoskeletal perturbation, and association with the endothelial cells. The transposon mutant library provided sufficient genome coverage and high-resolution mapping of gene function. The comparison between input and non-adherent output pools revealed significant shifts in mutant abundance, indicating efficient selection pressure and successful enrichment for adherence-associated genes. We identified multiple genes potentially involved in adherence and invasion of endothelial cells. Only three LPXTG-motif containing surface proteins passed our applied threshold (logFC > 2, q < 0.01), providing evidence that they are critical for adherence. Among these, a fibronectin-binding protein with high homology to known serum opacity factors (SOFs) emerged as a promising candidate for further functional validation. ScSOF was selected due to its predicted fibronectin-binding capacity and homology to virulence-associated SOFs in other streptococcal species.

Deletion of *sof* confirmed the role of ScSOF in mediating endothelial cell adherence and invasion. Under flow conditions that mimic vascular shear stress, wild type IMT49926 induced substantial cytoskeletal rearrangement and bacterial adherence, whereas the *sof* deletion mutant showed significantly reduced attachment and minimal host cytoskeletal disruption. This phenotype was consistently observed under static conditions and at different MOIs, confirming the importance of ScSOF for endothelial cell adherence, independent of flow-induced responses. The pronounced cytoskeletal alterations observed during wild type infection further support a role for ScSOF in active host-cell interaction rather than passive surface attachment alone. Such cytoskeletal remodeling may facilitate endothelial invasion, barrier disruption, or stabilization of bacterial colonization under shear stress conditions.

The signature characteristic of SOF proteins is their ability to opacify serum. Our data demonstrated that cell-associated ScSOF contributes to serum opacification, with both covalent and non-covalent surface associations depending on the bacterial growth phase. Hereby, detection of ScSOF in SDS extracts from overnight cultures suggests a non-covalent association to the bacterial surface, although the observed increase in extractable ScSOF protein might reflect a combination of altered surface association and protein release, which is mediated by bacterial lysis.

In addition to loss of opacification activity, the *sof* deletion mutant displayed enhanced β-hemolysis on blood agar, implicating a role for ScSOF in the modulation of hemolytic activity. This suggests that ScSOF has similar functional properties as SOF of *S. pyogenes*, which is also described as inhibitor of β-hemolytic activity (Zhu et al., 2017). The multifunctional phenotype associated with ScSOF suggests that this protein may contribute to several interconnected stages of host interaction during infective endocarditis development. Fibronectin binding may facilitate initial endothelial attachment, while effects on surface architecture and cytoskeletal perturbation could promote stable colonization and invasion under vascular conditions. In parallel, serum opacification activity and modulation of ß-hemolysis may influence host lipid interactions and bacterial fitness within the bloodstream environment.

Electron microscopy highlighted the role of ScSOF as a major surface-associated factor. SEM and TEM imaging demonstrated a significant reduction in the thickness and complexity of surface structures in the *sof* deletion mutant compared to the wild type, suggesting ScSOF contributes either directly to the extracellular matrix or indirectly by recruiting other surface-associated materials.

Binding assays validated the contribution of ScSOF to the fibronectin-binding ability of *S. canis*. Flow cytometry with FITC-labelled fibronectin and immunofluorescence microscopy showed a reduction in fibronectin binding by the 49926Δ*sof* strain, particularly at earlier time points, reinforcing the importance of ScSOF in initiating host interactions. Although this difference diminishes over time, likely due to compensatory mechanisms by other fibronectin-binding surface proteins or nonspecific binding, early adhesion events are critical in pathogenesis, particularly for infective endocarditis (IE), where initial endothelial attachment is a prerequisite for disease progression (Chorianopoulos et al., 2009). Other binding characteristics of ScSOF could be tested in further studies, as SOF of *S. pyogenes* has for example been shown to bind to fibulin-1, another extracellular host matrix protein (Courtney et al., 2009).

Consistent with reduced cell attachment, CSMA analyses showed that the *sof* deletion mutant had significantly better cell layer generation compared to the wild type. In the absence of infection, HUVEC monolayers fully closed the cell-free gap within 48 h under microfluidic conditions. Infection with wild type severely impaired both proliferation and migration, causing wound expansion rather than closure. In contrast, the *sof* deletion mutant allowed significantly increased regeneration compared to the wild type after 24 hours, but both infected conditions inhibited wound closure after 48 hours. The reduced adherence of the mutant likely contributes to its diminished impact on endothelial repair. Overall, these results demonstrate that SOF expression in *S. canis* is a key factor in disrupting endothelial wound healing under flow conditions, making it an important bacterial factor in the development of infective endocarditis.

We further showed that the *sof* deletion mutant had a decreased adherence to epithelial cells using A549 lung epithelial cells. This is in line with results from the SOF protein of *S. pyogenes*, which was shown to play a role in adherence to different epithelial cell lines, mainly by adherence to the host target matrix component fibronectin (Oehmcke et al., 2004). It was also shown for *S. pyogenes* that next to the decrease in adherence to epithelial cells, elimination of SOF led to a decrease of virulence in a murine model of necrotizing skin infection (Timmer et al., 2006). Because the ScSOF has a high similarity to the SOF of *S. pyogenes*, it is likely that ScSOF plays a similar role in *S. canis*, where a decrease in virulence could be expected upon deletion of the *sof* gene.

Population genomic analysis demonstrated that *sof* is unevenly distributed across the analyzed *S. canis* population and is predominantly associated with the BAPS 1 and 6 lineages. The *sof* gene is conserved amongst the BAPS 1 cluster. While the clustering of *sof*-positive isolates may be consistent with phylogenetic inheritance patterns, alternative explanations such as horizontal gene transfer, recombination, or historical gene gain and loss events cannot be excluded. Importantly, the restricted distribution of *sof* among distinct lineages may indicate that ScSOF-associated endothelial interaction phenotypes are not equally represented across the species population. Future comparative genomic and functional studies integrating recombination analyses, genomic context investigation, and virulence phenotyping will be necessary to clarify the evolutionary dynamics and potential clinical significance of *sof* within *S. canis* populations.

Overall, our findings identified ScSOF as a multifunctional surface protein of *S. canis* that plays a role in IE pathophysiology. Our data emphasize the necessity of investigating bacterial–endothelial interactions under physiological flow. Static culture fails to recapitulate shear stress–induced mechanotransduction, including upregulation of adhesion molecules (ICAM-1, VCAM-1) and mechanosensitive complexes (PECAM-1, VE-cadherin, VEGFR2) (Conway and Schwartz, 2012; Davies et al., 1997), whereas flow conditions induce endothelial phenotypes that better reflect *in vivo* hemodynamics (Samijo et al., 1998). SOF was identified as a key adhesion factor under both static and flow conditions, supporting its relevance in bloodstream infections and infective endocarditis, where adhesion occurs under shear stress. This mechanism is underscoring the importance of hydrodynamic models for translational relevance. CSMA under flow further demonstrated that wild type infection markedly impaired endothelial wound healing, while the *sof* mutant partially restored regenerative capacity. This shear-dependent phenotype highlights the role of SOF in endothelial dysfunction, indicating that it contributes not only to adhesion but also to impaired vascular repair during *S. canis* infection and is involved in the first step of infection. Building on this, SOF represents a promising target for therapeutic intervention. Inhibition of SOF-mediated adhesion at early stages of infection could prevent stable endothelial attachment and subsequent biofilm formation under shear conditions. Moreover, its surface exposure and functional relevance make SOF a potential candidate for vaccine development aimed at limiting vascular colonization and systemic dissemination.

Future work should explore the precise molecular interactions between ScSOF and fibronectin and other host matrix proteins such as fibulin, the structural biology of ScSOF itself, and its regulation under *in vivo* conditions. Additionally, given its similarity to SOFs in *S. pyogenes* and *S. dysgalactiae*, comparative functional analyses may reveal conserved virulence strategies across pyogenic streptococci. The two other genes that were identified in the TraDIS analysis, an IgG-binding protein G homologue and the M-like protein SCM type II, are of interest for further research too. Streptococcal M proteins are key virulence factors and often found to play a role in the interaction with host cells and host matrix proteins (Oehmcke et al., 2010; Fulde et al., 2011; Bergmann et al., 2017; Lapschies et al., 2023). The IgG-binding protein G homologue could potentially bind IgG and albumin, properties that can promote formation of vegetations by facilitating adhesion to fibrin-platelet aggregates on damaged heart valves (Nygren et al., 1988; Talay et al., 1996; Douglas and Ford, 1997).

## Data Availability

The datasets presented in this study can be found in online repositories. The names of the repository/repositories and accession number(s) can be found in the article/**Supplementary Material**.

## Supplementary material

Supplementary Figure 1 Analysis of the sof gene in Artemis. Top bar shows transposon insertions that were found in this gene within the transposon mutant library.

Supplementary Figure 2 Insufficient separation of non-adherent and adherent transposon mutant after first infection round. Volcano plot of a comparative analysis of the generated output pool (non-adherent bacteria after first selection round) and the input library showing the logFC of all genes after the first round of infection compared to the input pool (control).

Supplementary Figure 3 Comparison of IMT49926 wild type and isogenic *sof* knockout mutant. **(A)** Whole genome sequence of IMT49926 wild type (inner circles) and BLAST comparison with the 49926Δ*sof* in the outer circle with the dark red colour indicating the similarity in percentage. A red arrow is added to indicate the location of the *sof* gene deletion. Visualization was done with Proksee. **(B)** Growth curves of IMT49926 wild type and 49926Δsof in Todd Hewitt Broth and Endothelial Cell Growth Medium. No difference was observed in growth between the deletion mutant and the wild type.

Supplementary Figure 4 Comparison of ScSOF with similar proteins and protein families. **(A)** Interpro search of ScSOF for representative protein families. **(B)** Alignment of fibronectin binding repeats (red boxes) using a reference protein sequence of SOF in *S. dysgalactiae* and *S. pyogenes*. **(C)** BLAST results for *sof* gene nucleotide sequence of IMT49926 using the core nucleotide database of NCBI. **(D)** Protein BLAST result of ScSOF using the ClusteredNR database of NCBI.

Supplementary Figure 5 A549 epithelial cells after infection with IMT49926 and 49926Δ*sof* at MOI 5. Staining was executed as in the HUVEC infection experiment, but phalloidin staining is visualized in white. Adherent bacteria are shown in yellow and internalized bacteria in red as before. Scale bar represents 10 µm. Imaged with a DMI6000B fluorescence microscope at 630x magnification. A quantification of the total CFU after infection is shown on the right. *** indicates p < 0.001.

Supplementary Figure 6 Series of scanning electron and transmission electron microscopic images showing notable difference in the thickness of bacterial surface structures. **(A)** SEM visualization of IMT49926 in the top panel and of the 49926Δ*sof* mutant in the bottom panel. Scale bars indicate 1 µm. **(B)** TEM images of IMT49926 (top) and the 49926Δ*sof* mutant (bottom). Scale bars indicate 1 µm (left panel) or 200 nm (right panel). **(C)** SEM images show the measurement of bacterial surface structure thickness.

Supplementary Table 1 Output pool data with logFC and q values after first and second selection steps.

Supplementary Table 2 List of transposon mutants of 94 genes that fit the logFC>2 and q<0.01 conditions after the second infection selection step.

Supplementary Table 3 List of LPxTG anchoring-domain containing genes with their corresponding logFC and q values.
