# Supplementary figures and images for: Discovery of a new fibronectin-binding surface protein of Streptococcus canis with serum opacification activity through transposon directed insertion-site sequencing
Source: Front Cell Infect Microbiol. 2026 Jun 29;16:1867913. doi: 10.3389/fcimb.2026.1867913 (PMC13357522; doi:10.3389/fcimb.2026.1867913)

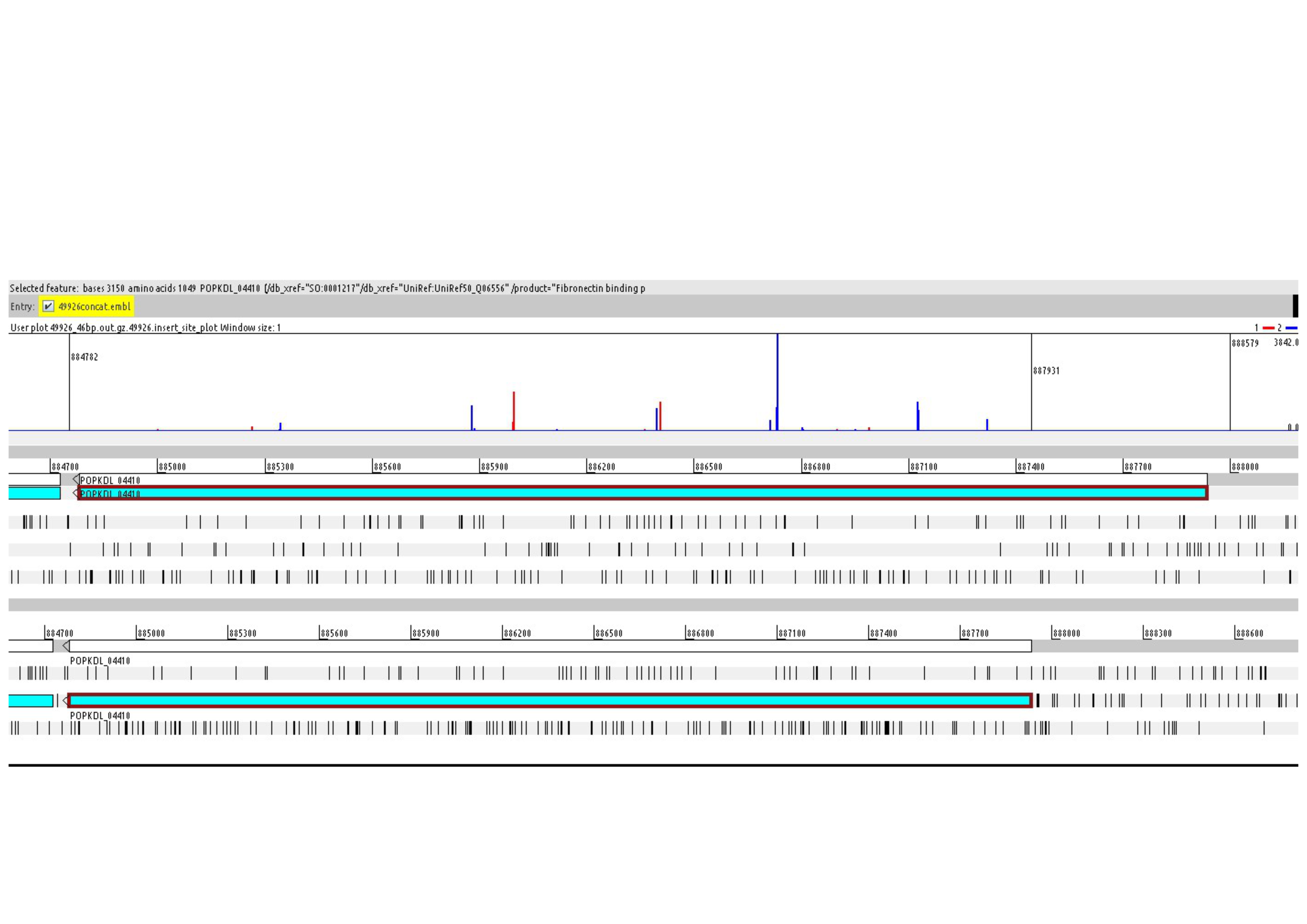

Supplement: Supplementary Figure 1 — Analysis of the sof gene in Artemis. Top bar shows transposon insertions that were found in this gene within the transposon mutant library. [file Image1.tiff]

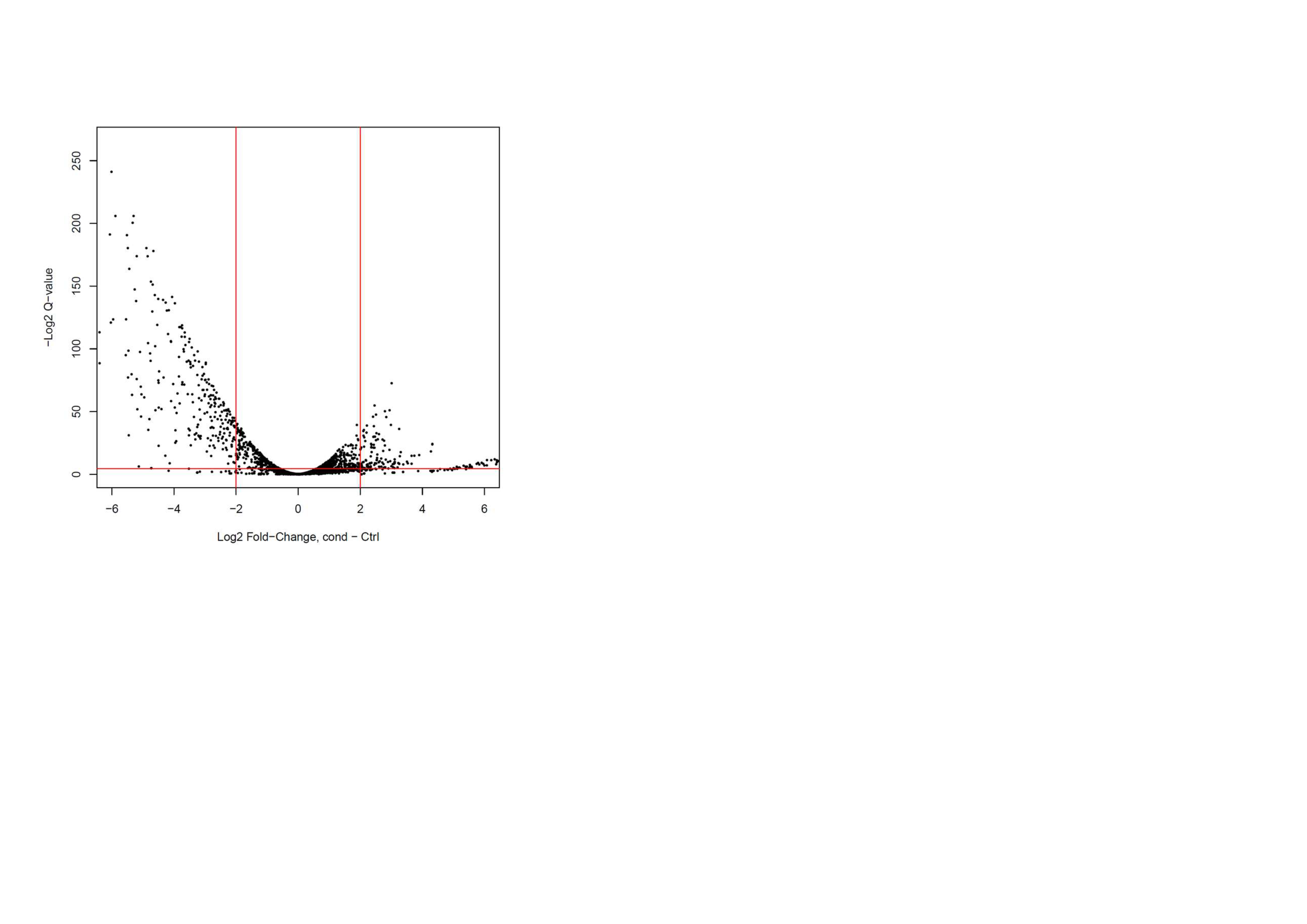

Supplement: Supplementary Figure 2 — Insufficient separation of non-adherent and adherent transposon mutant after first infection round. Volcano plot of a comparative analysis of the generated output pool (non-adherent bacteria after first selection round) and the input library showing the logFC of all genes after the first round of infection compared to the input pool (control). [file Image2.tiff]

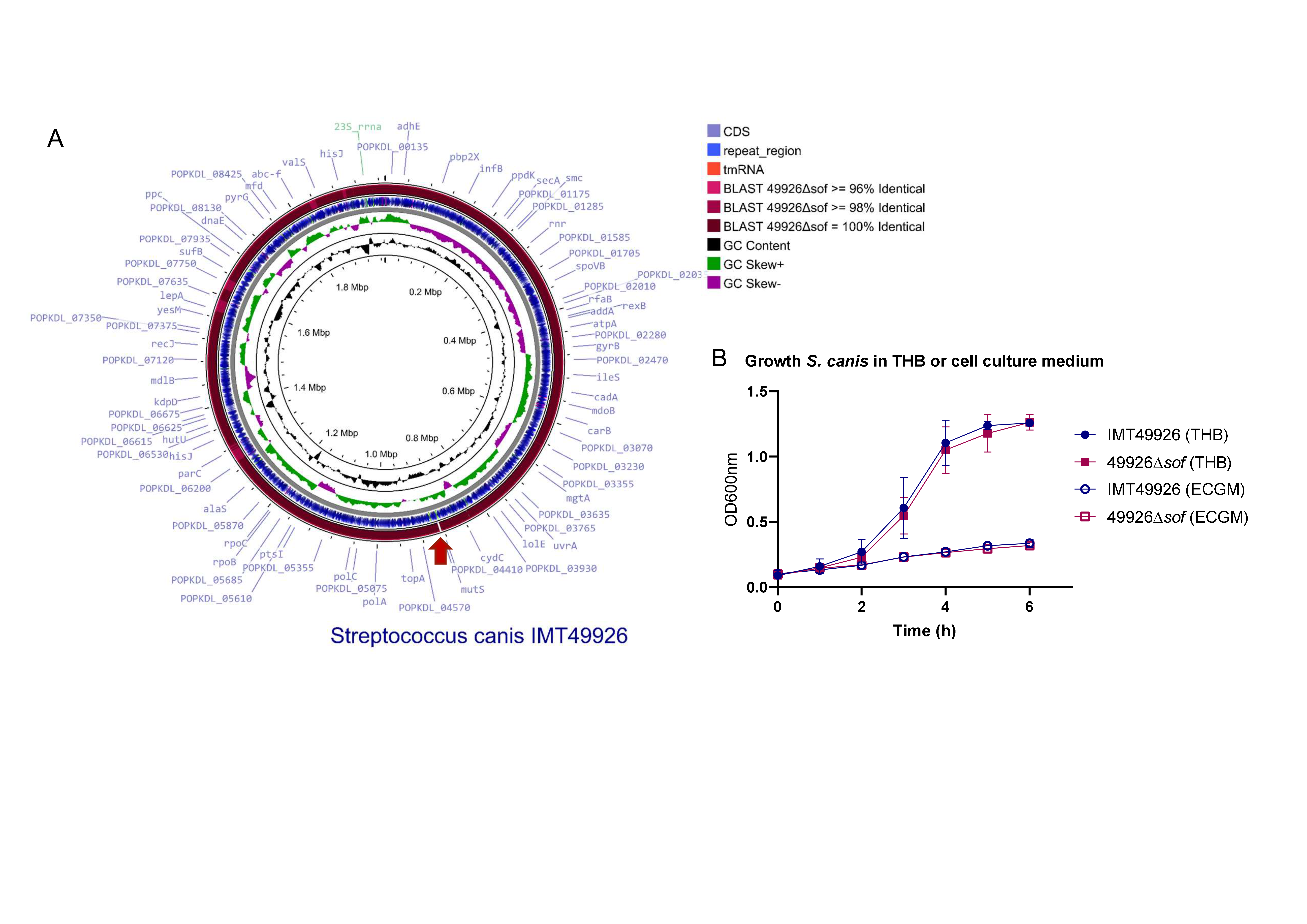

Supplement: Supplementary Figure 3 — Comparison of IMT49926 wild type and isogenic sof knockout mutant. (A) Whole genome sequence of IMT49926 wild type (inner circles) and BLAST comparison with the 49926Δsof in the outer circle with the dark red colour indicating the similarity in percentage. A red arrow is added to indicate the location of the sof gene deletion. Visualization was done with Proksee. (B) Growth curves of IMT49926 wild type and 49926Δsof in Todd Hewitt Broth and Endothelial Cell Growth Medium. No difference was observed in growth between the deletion mutant and the wild type. [file Image3.tiff]

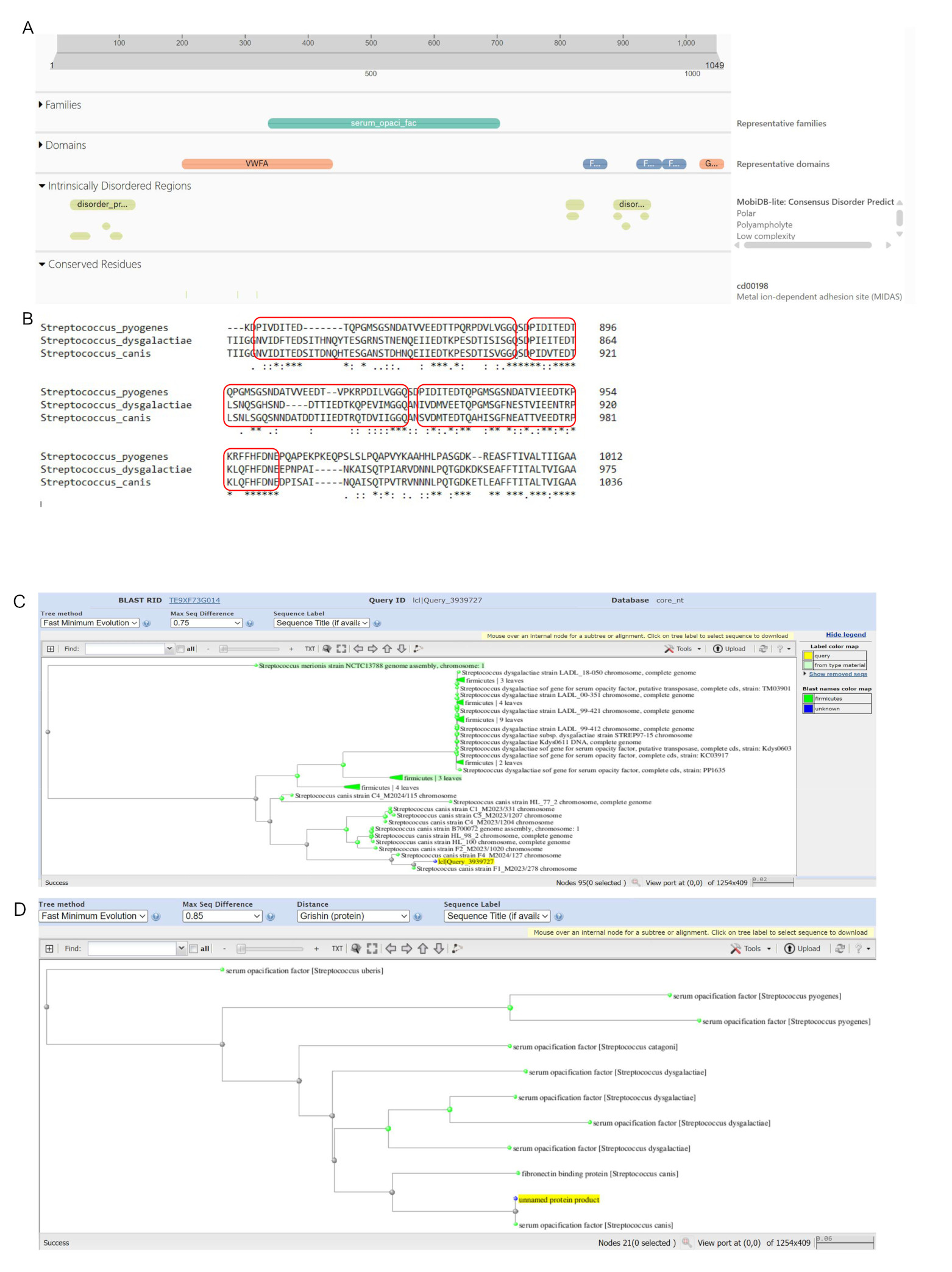

Supplement: Supplementary Figure 4 — Comparison of ScSOF with similar proteins and protein families. (A) Interpro search of ScSOF for representative protein families. (B) Alignment of fibronectin binding repeats (red boxes) using a reference protein sequence of SOF in S. dysgalactiae and S. pyogenes. (C) BLAST results for sof gene nucleotide sequence of IMT49926 using the core nucleotide database of NCBI. (D) Protein BLAST result of ScSOF using the ClusteredNR database of NCBI. [file Image4.tiff]

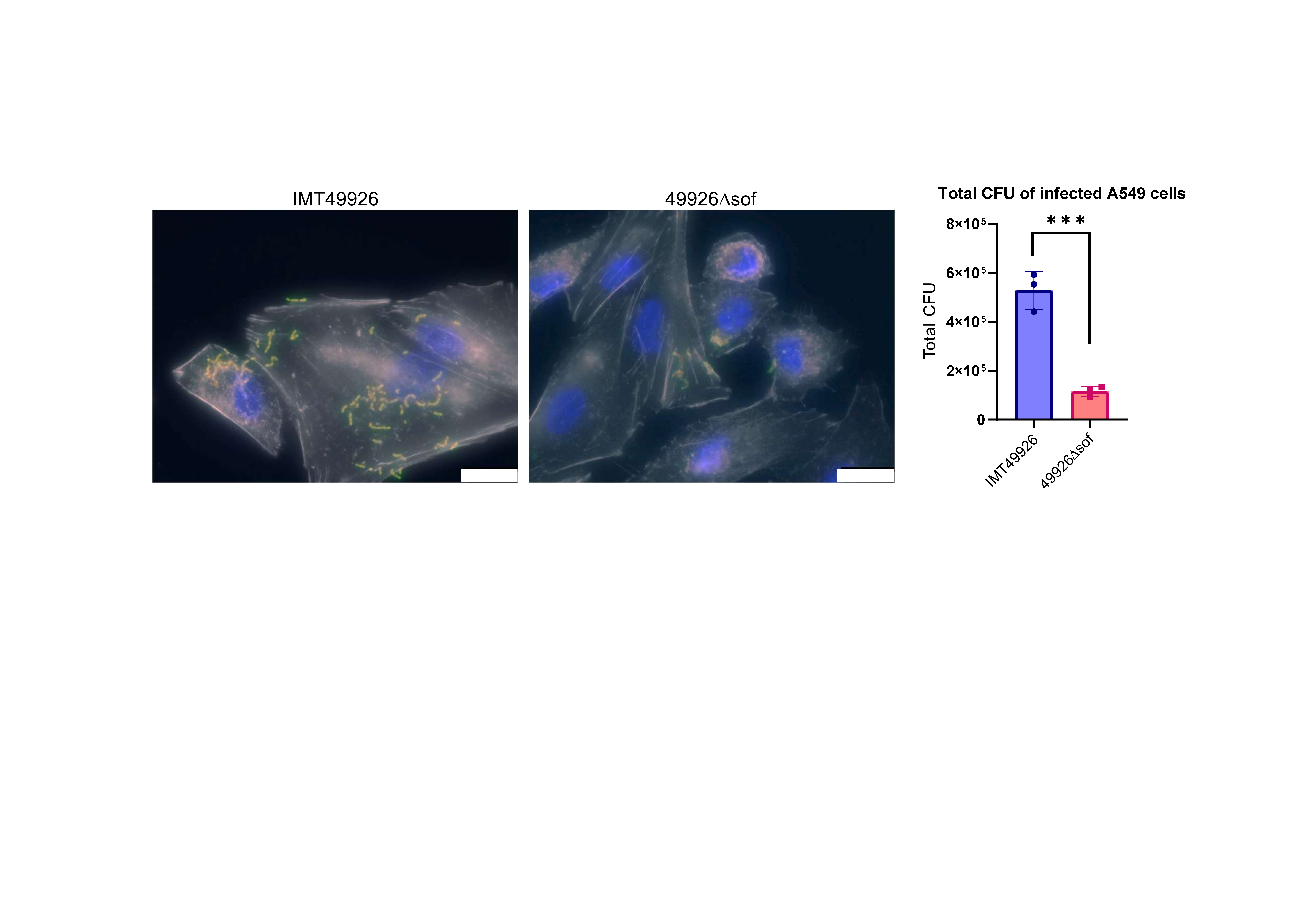

Supplement: Supplementary Figure 5 — A549 epithelial cells after infection with IMT49926 and 49926Δsof at MOI 5. Staining was executed as in the HUVEC infection experiment, but phalloidin staining is visualized in white. Adherent bacteria are shown in yellow and internalized bacteria in red as before. Scale bar represents 10 µm. Imaged with a DMI6000B fluorescence microscope at 630x magnification. A quantification of the total CFU after infection is shown on the right. *** indicates p < 0.001. [file Image5.tiff]

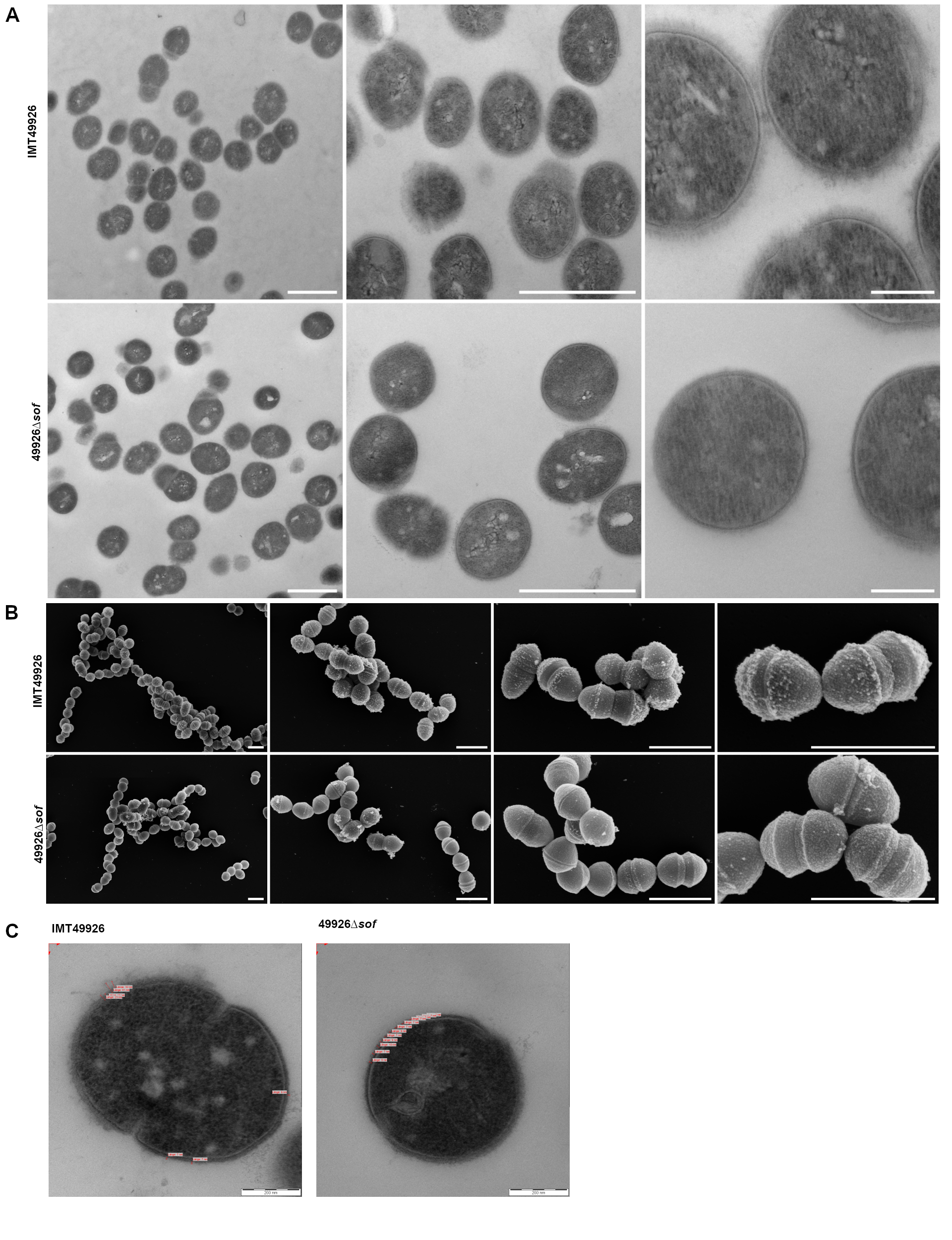

Supplement: Supplementary Figure 6 — Series of scanning electron and transmission electron microscopic images showing notable difference in the thickness of bacterial surface structures. (A) SEM visualization of IMT49926 in the top panel and of the 49926Δsof mutant in the bottom panel. Scale bars indicate 1 µm. (B) TEM images of IMT49926 (top) and the 49926Δsof mutant (bottom). Scale bars indicate 1 µm (left panel) or 200 nm (right panel). (C) SEM images show the measurement of bacterial surface structure thickness. [file Image6.tif]
