# Supplementary material for: Discovery of a new fibronectin-binding surface protein of Streptococcus canis with serum opacification activity through transposon directed insertion-site sequencing
Source: Front Cell Infect Microbiol. 2026 Jun 29;16:1867913. doi: 10.3389/fcimb.2026.1867913 (PMC13357522; doi:10.3389/fcimb.2026.1867913)
Supplement: Supplementary Table 1 — Output pool data with logFC and q values after first and second selection steps. [file DataSheet1.pdf]

locus_tag	gene_name	function	COG	logFC (first	q.value (fir	logFC (seco	q.value (second selection)
POPKDL_0(	POPKDL_0(	rod shape-(	M	0,335847	0,302682	0,842239	0,315604
POPKDL_0(	mreD	rod shape-(	M	0,508618	0,136645	1,874105	0,009193
POPKDL_0(	POPKDL_0(	CHAP doma	S	0,444077	0,517463	1,664149	0,181468
POPKDL_0(	prs1	ribose-phos	F	1,335564	0,10117	1,207863	0,451627
POPKDL_0(	recO	DNA repair	L	0,222026	0,721391	2,626162	0,126387
POPKDL_0(	plsX	phosphate	I	1,492963	0,180882	2,389736	0,207374
POPKDL_0(	acpP	acyl carrier protein		4,8961	0,043495	3,647743	0,47521
POPKDL_0(	purC	phosphorib	F	-1,16533	0,01665	-1,0187	0,307555
POPKDL_0(	POPKDL_0(	phosphorib	F	-2,16185	4,85E-05	-1,89662	0,019911
POPKDL_0(	purF	amidophos	F	1,42023	0,041081	2,257122	0,054075
POPKDL_0(	purM	phosphorib	F	0,48866	0,553521	1,432475	0,308093
POPKDL_0(	purN	phosphorib	F	1,85932	0,053437	3,751014	0,0052
POPKDL_0(	purH	bifunctiona	F	0,059407	1	0,483434	0,719207
POPKDL_0(	POPKDL_0(	amidase	S	0,743173	0,028724	0,784644	0,121589
POPKDL_0(	purD	phosphorib	F	-0,16072	0,759497	0,756848	0,567457
POPKDL_0(	purE	5-(carboxya	F	1,381406	0,128156	-2,15728	0,289313
POPKDL_0(	purK	5-(carboxya	F	1,733037	0,025077	1,941092	0,128924
POPKDL_0(	POPKDL_0(	hypothetical protein		-9,06417	3,40E-50	-5,08262	1,02E-07
POPKDL_0(	purB	adenylosuc	F	1,408965	0,024542	0,975602	0,434621
POPKDL_0(	rgg4	transcriptional regulato		-0,25257	0,450757	-0,49439	0,441294
POPKDL_0(	POPKDL_0(	hypothetica	K	4,275906	3,19E-06	1,705844	0,480924
POPKDL_0(	ruvB	Holliday jur	L	0,404976	0,607943	-0,24255	1
POPKDL_0(	POPKDL_0(	low molecu	T	-0,4128	0,225501	0,325532	0,685776
POPKDL_0(	POPKDL_0(	1-phosphat	S	-3,52959	1,13E-11	-3,98554	1,08E-10
POPKDL_0(	POPKDL_0(	acetyltrans	I	1,028362	0,005559	1,65084	0,004322
POPKDL_0(	adhE	bifunctiona	C	1,737296	0,000162	4,009893	7,45E-07
POPKDL_0(	adhP	alcohol deh	C	-1,22274	1,72E-05	-1,23924	0,037527
POPKDL_0(	POPKDL_0(	Threonine s	E	0,173337	0,749714	2,30952	0,178126
POPKDL_0(	POPKDL_0(	Threonine s	E	0,72292	0,787211	0,932898	0,78672
POPKDL_0(	POPKDL_0(	MATE famil	V	1,184195	0,00578	0,971504	0,196229

POPKDL_0(	POPKDL_0(	IS30 family	L	-3,25072	0,377489	-2,69135	0,672526
POPKDL_0(	rpsJ	30S ribosor	J	6,329409	0,000249		
POPKDL_0(	rplC	50S ribosor	J	1,219047	0,300825	0,227471	0,87215
POPKDL_0(	rplD	50S ribosor	J	1,116738	0,275862	1,515774	0,447154
POPKDL_0(	rplW	50S ribosor	J	1,314325	0,622144	0,970031	0,605818
POPKDL_0(	rplB	50S ribosor	J	4,320544	6,69E-08	2,099333	0,315279
POPKDL_0(	rpsS	30S ribosor	J	2,124151	0,622144	5,709524	0,035683
POPKDL_0(	rplV	50S ribosor	J	-2,16099	0,610007	0,644106	0,872211
POPKDL_0(	rpsC	30S ribosor	J	-1,82569	0,414772	-0,50557	1
POPKDL_0(	rplP	50S ribosor	J	0,799639	0,333517	2,220991	0,106616
POPKDL_0(	rpmC	50S ribosor	J	5,207793	0,028154		
POPKDL_0(	rpsQ	30S ribosor	J	2,805184	0,020887	0,932875	0,78672
POPKDL_0(	rplN	50S ribosor	J	0,394846	0,85766	3,021043	0,101826
POPKDL_0(	rplX	50S ribosor	J	1,026438	0,52385	-0,3275	1
POPKDL_0(	rplE	50S ribosor	J	2,51126	0,060076	0,968945	0,605818
POPKDL_0(	rpsZ	type Z 30S r	J	5,392889	0,014379	4,245168	0,264335
POPKDL_0(	rpsH	30S ribosor	J	3,002938	0,01139	4,556751	0,016401
POPKDL_0(	rplF	50S ribosor	J	0,635545	0,673167	1,695208	0,384721
POPKDL_0(	rplR	50S ribosor	J	0,496344	0,474444	-0,02225	1
POPKDL_0(	rpsE	30S ribosor	J	0,297996	0,732509	-5,07704	0,094793
POPKDL_0(	rpmD	50S ribosor	J	0,141004	1	-4,11916	0,200876
POPKDL_0(	rplO	50S ribosor	J	6,527135	5,36E-05	4,789025	0,131772
POPKDL_0(	secY	preprotein t	U	2,710239	0,00149	1,077249	0,672526
POPKDL_0(	POPKDL_0(	adenylate k	F	-0,23212	1	2,760333	0,029437
POPKDL_0(	infA	translation	J	-0,55681	0,891545	-1,45596	0,675805
POPKDL_0(	rpmJ	50S ribosor	J	3,08883	0,396674		
POPKDL_0(	rpsM	30S ribosor	J	0,954168	0,408002	-4,51329	0,097049
POPKDL_0(	rpsK	30S ribosor	J	-0,10938	0,870019	1,739333	0,351517
POPKDL_0(	rpoA	DNA-direct	K	0,847785	0,418398	0,322634	0,926024
POPKDL_0(	rplQ	50S ribosor	J	-0,4745	1	-0,32742	1
POPKDL_0(	POPKDL_0(	Integrase c	L	0,701096	0,046833	0,002234	1

POPKDL_0(	hpf	Ribosome h	J	-0,58405	0,078103	-0,25986	0,675805
POPKDL_0(	POPKDL_0(	ComF oper	S	0,677991	0,060076	1,399667	0,010451
POPKDL_0(	POPKDL_0(	DNA/RNA h	L	-0,78426	0,005399	-0,78684	0,176043
POPKDL_0(	POPKDL_0(	YigZ family	S	0,653336	0,127846	0,00319	1
POPKDL_0(	cysK	cysteine sy	E	-2,27657	7,25E-12	-2,35884	0,000498
POPKDL_0(	POPKDL_0(	S1 RNA-bin	J	-0,93135	0,014485	-3,03812	0,007498
POPKDL_0(	POPKDL_0(	Putative bif	G	-2,8685	2,12E-22	-3,05003	4,03E-09
POPKDL_0(	citB	DNA-bindir	K	-5,35061	8,66E-20	-5,32221	5,04E-11
POPKDL_0(	POPKDL_0(	two-compo	T	0,968288	0,027257	0,906877	0,213477
POPKDL_0(	POPKDL_0(	transporter	S	-2,30889	7,15E-09	-1,03238	0,183365
POPKDL_0(	pknB	Stk1 family	K L T	-0,70213	0,315694	0,204924	0,939214
POPKDL_0(	pTC1	Stp1/IreP fa	T	1,55151	0,04657	-1,78061	0,631588
POPKDL_0(	rsmB	16S rRNA (c	J	0,25514	0,577078	0,385548	0,703606
POPKDL_0(	fmt	methionyl-t	J	0,803938	0,460128	3,19994	0,055203
POPKDL_0(	priA	primosoma	L	1,014002	0,085971	1,043164	0,41647
POPKDL_0(	rpoZ	DNA-direct	K	1,132752	0,230501	-4,51329	0,097049
POPKDL_0(	gmk	guanylate k	F	1,533957	0,317201	3,706643	0,016656
POPKDL_0(	rny	ribonucleas	D	1,85113	0,007619	1,957294	0,186225
POPKDL_0(	POPKDL_0(	acetyl-CoA	I	-1,36351	8,77E-06	-1,3662	0,025067
POPKDL_0(	POPKDL_0(	hypothetica	E G	0,932649	0,01285	0,787439	0,437463
POPKDL_0(	luxS	S-ribosylho	H	-1,89933	3,12E-11	-2,07821	3,55E-05
POPKDL_0(	POPKDL_0(	Mid-cell-an	D	2,36582	4,56E-06	1,866024	0,047396
POPKDL_0(	rlmL	RNA methy	L	1,91331	0,002047	1,485018	0,181176
POPKDL_0(	RNaseP_bact_b			-0,02202	0,870837	1,224094	0,667057
POPKDL_0(	gpsB	cell divisior	D	2,124906	0,622144	4,918392	0,090612
POPKDL_0(	POPKDL_0(	YspA SLOG	S	3,054842	0,019026	2,834743	0,152797
POPKDL_0(	recU	Holliday jur	L	-1,26599	1	0,934716	0,674084
POPKDL_0(	pbp1a	penicillin-b	M	0,477982	0,60045	1,192678	0,288245
POPKDL_0(	pepC	aminopepti	E	-0,54616	0,06437	-0,62738	0,223396
POPKDL_0(	nadE	ammonia-d	H	0,518807	0,73915	1,432417	0,447078
POPKDL_0(	pncB	nicotinate p	H	2,108137	0,001078	0,407619	0,951103

POPKDL_0(	ansP	amino acid	E	-1,22473	1,34E-05	-0,87826	0,097049
POPKDL_0(	trxB	thioredoxin	C	-0,23878	1	2,630977	0,051886
POPKDL_0(	POPKDL_0(	DUF4059 d	S	2,040494	1		
POPKDL_0(	glnQ	amino acid	E	-2,0191	0,254106	-2,55544	0,264974
POPKDL_0(	hisM	amino acid	P	3,504286	0,000923	3,710954	0,051711
POPKDL_0(	POPKDL_0(	amino acid	E T	0,66639	0,03978	0,625387	0,376327
POPKDL_0(	srmB	DEAD/DEAI	J K L	1,675677	0,121095	1,145302	0,563062
POPKDL_0(	mraY	phospho-N	M	3,897386	2,43E-05	3,880086	0,021346
POPKDL_0(	pbp2X	penicillin-b	M	0,957311	0,148574	1,938353	0,057955
POPKDL_0(	ftsL	cell divisior	D	0,271634	0,881368	-4,11916	0,200876
POPKDL_0(	rsmH	16S rRNA (c	J	-1,15071	0,115086	1,959648	0,207374
POPKDL_0(	POPKDL_0(	Gamma-glu	M	0,968739	0,00628	0,883861	0,319113
POPKDL_0(	proA	glutamate-!	E	-2,31368	5,11E-16	-2,19549	4,46E-06
POPKDL_0(	proB	Glutamate	E	-1,85831	5,07E-11	-1,34025	0,031156
POPKDL_0(	POPKDL_0(	ABC transp	V	-2,5107	9,32E-15	-2,70902	9,25E-08
POPKDL_0(	ccmA	3-dehydroq	Z	0,371922	0,283053	0,657963	0,264858
POPKDL_0(	POPKDL_0(	bacteriocin	S	0,346489	0,430859	-0,18052	0,871182
POPKDL_0(	tkt	transketola	G	0,504237	0,446435	1,575525	0,26613
POPKDL_0(	fsa	fructose-6-	H	1,392901	0,003744	3,176035	0,001293
POPKDL_0(	POPKDL_0(	DNA-bindir	K	-2,16364	9,78E-13	-2,36082	3,77E-05
POPKDL_0(	fadH2	NADH pero	C	0,808073	0,004319	0,75442	0,184307
POPKDL_0(	glpF	aquaporin	U	-3,0121	1,69E-19	-3,50339	0,000882
POPKDL_0(	glpO	type 1 glyce	C	-1,79055	1,11E-06	-2,13743	5,25E-05
POPKDL_0(	glpK	glycerol kin	F	-2,38028	1,44E-10	-1,83808	0,003193
POPKDL_0(	POPKDL_0(	Uncharacte	T	2,003865	1,11E-06	3,724564	3,32E-07
POPKDL_0(	POPKDL_0(	DUF896 do	S	5,04568	0,046824	2,677054	0,675805
POPKDL_0(	glyS	Glycine--tR	J	1,850759	0,006113	2,250427	0,053736
POPKDL_0(	glyQ	glycine--tRI	J	2,473919	0,00216	2,313641	0,177576
POPKDL_0(	ypbQ	Isoprenylcy	S	-0,39282	0,319671	-1,04421	0,148003
POPKDL_0(	POPKDL_0(	2,5-diketo-I	C	-0,62475	0,147916	-0,26609	0,799968
POPKDL_0(	nagA	N-acetylglu	G	-1,68599	2,03E-08	-2,33958	5,52E-05

POPKDL_0(	nptA	sodium-de|	P	-1,26138	2,97E-05	-0,87842	0,177576
POPKDL_0(	POPKDL_0(	Putative lipoprotein		-3,14961	7,78E-14	-3,02387	5,76E-09
POPKDL_0(	POPKDL_0(	fatty acid-b	S	1,303222	0,010375	0,072836	0,951419
POPKDL_0(	POPKDL_0(	TetR family	K	1,398021	0,00097	0,996488	0,336526
POPKDL_0(	cof	Cof-type HAD-IIB family		-1,03119	0,000852	-1,49786	0,024062
POPKDL_0(	yadS	HAD superf	S	-1,30893	0,001839	-1,73357	0,002587
POPKDL_0(	POPKDL_0(	protein lac)	G	-3,69162	9,49E-31	-3,45017	1,66E-08
POPKDL_0(	lacD	tagatose-bi	G	-13,4461	2,95E-74	-9,22031	2,46E-23
POPKDL_0(	lacCpfkB	tagatose-6-	H	0,791398	0,005767	1,23639	0,021539
POPKDL_0(	lacB	galactose-6	G	1,090576	0,009366	0,60413	0,459387
POPKDL_0(	lacA	galactose-6	G	0,295394	0,701419	1,374908	0,197981
POPKDL_0(	sgcC	PTS galacti	G	-0,1571	0,632799	-0,25591	0,75758
POPKDL_0(	sgaB	PTS fructos	G	0,976388	0,139306	1,623223	0,218698
POPKDL_0(	ptsN	PTS galacto	G	-0,30592	0,364987	-0,17081	0,822374
POPKDL_0(	glpR	DeoR family transcripti		-1,45305	0,028479	-0,66627	0,590799
POPKDL_0(	POPKDL_0(	carbonate (	K?	-9,10429	1,01E-29	-8,05375	1,51E-11
POPKDL_0(	cadA	heavy meta	P	0,410363	0,254106	0,234441	0,756621
POPKDL_0(	POPKDL_0(	CopY/TcrY t	K	0,844223	0,032638	0,473073	0,602004
POPKDL_0(	POPKDL_0(	esterase	I	0,385094	0,204764	0,505296	0,33811
POPKDL_0(	POPKDL_0(	hypothetical protein		-2,06913	1,33E-09	-2,6828	3,22E-05
POPKDL_0(	rbfA	30S ribosor	J	0,016438	0,911641	-4,82253	0,051886
POPKDL_0(	infB	translation	J	1,969234	0,004255	2,654832	0,034324
POPKDL_0(	rpl7Ae	YlxQ-relate	J	-1,4853	0,606661	0,226966	0,87215
POPKDL_0(	ylxR	DUF448 do	K	2,336994	0,012117		
POPKDL_0(	nusA	transcriptic	K	0,313288	0,67784	3,359131	0,008816
POPKDL_0(	rimP	ribosome m	J	-3,25072	0,377333	2,37224	0,051886
POPKDL_0(	trmB	tRNA (guan	J	-2,8023	1,09E-19	-1,16169	0,144942
POPKDL_0(	cotS	aminoglycc	M	2,750785	0,030061	2,998621	0,055579
POPKDL_0(	POPKDL_0(	multidrug A	U	-0,03369	0,946803	0,814061	0,319113
POPKDL_0(	POPKDL_0(	multidrug A	V	-0,17062	0,68902	-0,64152	0,422697
POPKDL_0(	POPKDL_0(	HIT family p	F G	1,603807	2,54E-05	1,197596	0,110805

POPKDL_0(	POPKDL_0(	chemotaxis protein		0,492041	0,353252	0,255528	0,871409
POPKDL_0(	POPKDL_0(	transporter	K	1,935402	0,003866	2,076532	0,078824
POPKDL_0(	POPKDL_0(	N-acetyltra	K	0,653329	0,074335	0,297707	0,73742
POPKDL_0(	tsaE	tRNA (aden	M	0,008418	0,922934	-0,57065	1
POPKDL_0(	POPKDL_0(	guanine pe	S	-2,25834	3,60E-13	-2,70648	2,30E-07
POPKDL_0(	cof	HAD family	S	0,705603	0,075158	0,594001	0,556005
POPKDL_0(	SSRC34_2			1,32201	0,364462	-0,30899	0,862941
POPKDL_0(	SSRC34_1			-7,99284	1,73E-64	-7,44797	9,43E-13
POPKDL_0(	manX	PTS system	G	0,820745	0,125061	0,946562	0,291863
POPKDL_0(	manY	PTS manno	G	0,466621	0,438646	-0,29276	0,799968
POPKDL_0(	manZ	PTS manno	G	0,955114	0,135753	1,308695	0,220074
POPKDL_0(	POPKDL_0(	DUF956 do	S	-3,51786	4,49E-10	-1,39727	0,191141
POPKDL_0(	POPKDL_0(	hypothetica	S	-2,81826	7,02E-12	-2,64028	6,24E-05
POPKDL_0(	POPKDL_0(	Membrane	S	-8,87108	3,17E-73	-7,49809	1,67E-12
POPKDL_0(	serS	serine--tRN	J	0,478072	0,601655	2,603505	0,044496
POPKDL_0(	accA	acetyl-CoA	I	0,763194	0,31402	1,622245	0,243672
POPKDL_0(	accD	acetyl-CoA	I	-2,00962	0,359041	-2,1572	0,289313
POPKDL_0(	accC	acetyl-CoA	I	-0,19134	0,861235	-1,49784	0,371712
POPKDL_0(	fabZ	3-hydroxya	I	1,200562	0,520462	0,644077	0,872211
POPKDL_0(	accB	acetyl-CoA	I	2,780002	0,021137	1,133373	0,634516
POPKDL_0(	fabF	beta-ketoa	I	0,010204	0,820115	2,057199	0,110023
POPKDL_0(	fabG	3-oxoacyl-[	I Q	0,750161	0,453973	-0,84115	1
POPKDL_0(	fabD	ACP S-malo	I	1,212904	0,162213	-0,82412	0,84546
POPKDL_0(	fabK	enoyl-[acyl-	S	0,755588	0,437246	-1,38948	0,862941
POPKDL_0(	acpP	acyl carrier	I Q	4,314684	0,148235		
POPKDL_0(	fabH	3-oxoacyl-A	I	1,657517	0,125331	0,809759	0,60228
POPKDL_0:	marR	MarR family	K	3,106528	0,002129	-0,02257	1
POPKDL_0:	POPKDL_0:	enoyl-CoA I	I	2,620506	0,015697	2,049699	0,278301
POPKDL_0:	dnaJ	molecular c	O	1,45469	0,18428	0,865004	0,757905
POPKDL_0:	POPKDL_0:	hypothetical protein		5,50761	0,030764		
POPKDL_0:	dnaK	molecular c	O	2,237857	0,003993	1,982717	0,125639

POPKDL_0:	POPKDL_0:	Protein Grp	O	-1,99938	0,119131	3,266821	0,027947
POPKDL_0:	hrcA	heat-induci	K	2,308133	0,002088	-1,38893	0,862941
POPKDL_0:	POPKDL_0:	N-acetylmu	N U	-0,3945	0,296513	-0,36032	0,685776
POPKDL_0:	POPKDL_0:	D-alanyl-D-	M	1,118819	0,000216	0,680844	0,464523
POPKDL_0:	phoE	histidine ph	G	-4,61744	9,78E-44	-4,34264	8,18E-10
POPKDL_0:	POPKDL_0:	GlsB/YeaQ/	S	-2,43096	6,33E-13	-1,80274	0,002907
POPKDL_0:	POPKDL_0:	hypothetica	E G	0,644946	0,030574	1,004542	0,09115
POPKDL_0:	gatB	Asp-tRNA(A	J	1,672361	0,016044	1,328221	0,405743
POPKDL_0:	gatA	Asp-tRNA(A	J	1,608175	0,030417	1,856906	0,149488
POPKDL_0:	gatC	Asp-tRNA(A	J	2,127424	0,622144	4,2461	0,264974
POPKDL_0:	ppdK	pyruvate, p	G	-1,71907	1,79E-05	-1,47773	0,009193
POPKDL_0:	POPKDL_0:	phosphoen	S	-0,22964	0,534192	-0,21522	0,742113
POPKDL_0:	POPKDL_0:	CBS domai	K	0,726035	0,016224	0,674114	0,299866
POPKDL_0:	POPKDL_0:	rhodanese-	P	5,910762	0,001754	7,519719	0,00526
POPKDL_0:	POPKDL_0:	isochorism	Q	-2,40543	3,01E-17	-2,74563	6,84E-07
POPKDL_0:	codY	GTP-sensin	K	1,219879	0,341404	1,870653	0,236466
POPKDL_0:	aspB	aminotrans	E	-1,27223	0,043466	-0,72749	0,527025
POPKDL_0:	POPKDL_0:	universal st	T	-0,05879	0,902654	0,104401	0,996857
POPKDL_0:	POPKDL_0:	haloacid de	S	1,949797	0,00217	2,226198	0,000128
POPKDL_0:	POPKDL_0:	L-asparagir	E J	0,348541	0,455716	0,10178	0,883776
POPKDL_0:	recG	ATP-depend	L	-0,15263	1	1,31169	0,328321
POPKDL_0:	POPKDL_0:	HMP/thiam	P	-0,55779	0,0696	-0,8938	0,161136
POPKDL_0:	POPKDL_0:	cobalamin	P	-0,85547	0,003408	0,245455	0,772686
POPKDL_0:	POPKDL_0:	Hypothetica	S	-1,21003	0,000121	-1,52283	0,024771
POPKDL_0:	POPKDL_0:	ABC transp	V	0,811958	0,006577	1,317193	0,040125
POPKDL_0:	POPKDL_0:	ABC transp	V	-3,08002	2,13E-24	-2,84158	7,11E-07
POPKDL_0:	fepC	iron ABC tra	H P	2,359467	7,72E-08	3,111707	7,22E-08
POPKDL_0:	fepD	ABC transp	P	1,891074	1,44E-12	4,032055	0,000105
POPKDL_0:	isdE	heme ABC t	P	-4,88789	5,19E-55	-4,10021	1,87E-07
POPKDL_0:	POPKDL_0:	HemeBindi	S	0,822219	0,005731	1,904443	0,000222
POPKDL_0:	POPKDL_0:	amino acid	M	-1,49313	2,41E-07	-1,54417	0,005841

POPKDL_0:	POPKDL_0:	CHAP dom:	M	-1,97432	9,08E-07	-1,31994	0,05922
POPKDL_0:	alr	alanine rac	E	0,820775	0,244376	-0,98244	0,672526
POPKDL_0:	acpS	holo-ACP s	I	5,748049	0,003091		
POPKDL_0:	secA	preprotein	U	1,899533	0,000209	3,121059	0,01191
POPKDL_0:	POPKDL_0:	Transposas	L	-0,87553	0,001664	-0,94119	0,135384
POPKDL_0:	pspC	PspC doma	K T	2,126595	0,622144		
POPKDL_0:	POPKDL_0:	SprT family	S	0,273024	0,712091	-0,01073	1
POPKDL_0:	tex	RNA-bindin	K	-2,38151	4,86E-16	-2,20288	5,63E-06
POPKDL_0:	POPKDL_0:	Phosphate	P	-2,22188	0,171649	1,490983	0,197859
POPKDL_0:	cFF1	cupin	S	0,816221	0,298696	-0,61011	0,871409
POPKDL_0:	POPKDL_0:	ABC transporter ATP-bi		1,563042	0,038912	1,606102	0,230407
POPKDL_0:	yraQ	putative pe	S	-1,97405	1,39E-10	-1,03686	0,068426
POPKDL_0:	ycgQ	TIGR03943	S	2,716352	4,56E-09	2,766584	5,95E-06
POPKDL_0:	bglB	6-phospho-	G	1,396822	6,78E-06	1,218702	0,017646
POPKDL_0:	nagE	PTS beta-gl	G	0,665059	0,024402	0,65425	0,240907
POPKDL_0:	POPKDL_0:	transcriptic	K	0,724571	0,016063	0,877772	0,091423
POPKDL_0:	Spy490483c		E G P	0,922006	0,017022	0,849847	0,245041
POPKDL_0:	araJ	MFS transporter		-1,21971	2,34E-05	-1,18497	0,03809
POPKDL_0:	ftsY	signal reco	U	-0,39219	0,892638	0,49375	0,710279
POPKDL_0:	cof	Cof-type HAD-IIB family		0,77504	0,041143	0,629289	0,563062
POPKDL_0:	cof	HAD family hydrolase		-1,2698	0,001105	-2,1546	0,084768
POPKDL_0:	POPKDL_0:	serine prot	O	0,198736	0,556427	1,564724	0,024415
POPKDL_0:	smc	chromoson	D	-3,81682	9,72E-18	-3,5288	9,61E-06
POPKDL_0:	rnc	ribonucleas	J	2,13939	0,030061	1,957315	0,288118
POPKDL_0:	POPKDL_0:	MBL fold m	S	-5,19976	1,38E-23	-4,23761	2,44E-06
POPKDL_0:	POPKDL_0:	Histidine ki	T	1,101929	0,033818	0,82211	0,53313
POPKDL_0:	POPKDL_0:	Transcriptic	KT	1,771434	0,143761	1,44384	0,621417
POPKDL_0:	POPKDL_0:	DUF3114 d	S	0,520118	0,230211	-0,30608	0,779191
POPKDL_0:	POPKDL_0:	aquaporin family protei		0,436228	0,234372	0,066018	0,965905
POPKDL_0:	POPKDL_0:	PTS manno	G	0,841585	0,022125	0,486999	0,578991
POPKDL_0:	dhaL	dihydroxya	S	1,234562	3,89E-05	1,004938	0,09807

POPKDL_0:	dhaK	dihydroxya	G	0,473653	0,17416	1,859386	0,003714
POPKDL_0:	dhaS	dihydroxya	G	1,222525	0,000179	1,376789	0,066959
POPKDL_0:	dhaQ	DhaKLM op	G	0,196911	0,668902	-0,10331	0,927202
POPKDL_0:	POPKDL_0:	hypothetical protein		0,718142	0,173148	0,312131	0,851735
POPKDL_0:	POPKDL_0:	multidrug A	S	0,585342	0,060781	0,663605	0,230407
POPKDL_0:	POPKDL_0:	ABC transporter perme		0,723776	0,030593	0,763675	0,226326
POPKDL_0:	POPKDL_0:	sugar ABC transporter A		-3,39133	5,08E-18	-3,80752	8,04E-11
POPKDL_0:	thrS	threonine--	J	2,274331	0,024767	3,461753	0,00295
POPKDL_0:	POPKDL_0:	1,2-diacylg	M	1,10033	0,213814	1,369284	0,329139
POPKDL_0:	rfaB	glycosyl tra	M	-0,61296	0,856291	-1,43581	0,459814
POPKDL_0:	POPKDL_0:	alpha-amyl	G	2,438313	2,80E-12	3,251431	5,32E-11
POPKDL_0:	ccpA	catabolite c	K	-0,51528	0,657379	-0,54677	0,953488
POPKDL_0:	pepP	peptidase N	E	-0,79439	0,139224	-0,70577	0,365788
POPKDL_0:	nfnB	NAD(P)H-d	C	1,260675	4,97E-05	1,072495	0,217093
POPKDL_0:	gloA	lactoylgluta	E	-0,32112	0,509892	0,19821	0,8833
POPKDL_0:	POPKDL_0:	glycosyltrar	M	-0,96331	0,001326	-0,97483	0,061061
POPKDL_0:	POPKDL_0:	Cell division protein Fts		1,154307	0,160644	2,279769	0,139778
POPKDL_0:	smpB	SsrA-bindir	O	2,337983	0,017251	2,145612	0,220581
POPKDL_0:	POPKDL_0:	DEDD-Tnp-IS110 doma		0,551178	0,178842	0,691579	0,39816
POPKDL_0:	POPKDL_0:	IS110 famil	L	1,406583	0,186687	-0,91245	1
POPKDL_0:	rnr	ribonucleas	J	-2,90883	2,27E-09	-1,98765	0,001632
POPKDL_0:	secG	preprotein t	U	-0,04228	0,799056	0,053407	1
POPKDL_0:	POPKDL_0:	Multidrug resistance pr		-3,75431	9,45E-34	-3,64753	8,91E-14
POPKDL_0:	herA	ATP-binding	S	-1,86238	6,78E-07	-1,75768	0,000777
POPKDL_0:	POPKDL_0:	hypothetica	S	-0,48492	0,120696	-0,32032	0,605725
POPKDL_0:	POPKDL_0:	ABC transp	V	2,201262	9,88E-11	2,332983	6,10E-07
POPKDL_0:	coaE	dephospho	H	1,824943	0,009434	2,237582	0,16315
POPKDL_0:	mutM	DNA-forma	K	0,907758	0,003264	0,517983	0,545003
POPKDL_0:	rgg2	quorum-sensing systen		0,175708	0,622144	0,209822	0,775659
POPKDL_0:	POPKDL_0:	Peptide pheromone SHP2				5,914775	0,042729
POPKDL_0:	POPKDL_0:	transglutan	D	2,52072	1,33E-10	3,441697	1,64E-08

POPKDL_0:	POPKDL_0:	hypothetica	F	-0,96051	0,001326	-0,92856	0,097925
POPKDL_0:	POPKDL_0:	DNA mismatch repair p		0,577894	0,098351	0,2694	0,770311
POPKDL_0:	POPKDL_0:	hypothetical protein		2,211443	0,090216	3,287577	0,108781
POPKDL_0:	era	GTPase Era	M	1,065391	0,26315	2,507301	0,101399
POPKDL_0:	dgkA	UDP kinase	M	-1,03733	0,247233	0,488399	0,769904
POPKDL_0:	ybeY	rRNA matu	S	0,466881	0,67784	-0,46518	1
POPKDL_0:	udg4	uracil-DNA	L	1,74248	0,002766	-0,4069	0,964819
POPKDL_0:	phoH	phosphate	T	0,229798	0,501226	0,288167	0,675633
POPKDL_0:	POPKDL_0:	Myosin-cro	S	-0,16518	0,794383	-6,05551	0,012817
POPKDL_0:	POPKDL_0:	adhesin	M	-4,59694	4,19E-16	-4,61147	2,02E-11
POPKDL_0:	yozE	UPF0346 p	S	0,913927	0,142479	-0,41817	0,820414
POPKDL_0:	msrA	Peptide me	O	0,905101	0,006348	0,64419	0,365601
POPKDL_0:	cvfB	RNA-bindin	S	-3,41091	6,31E-20	-2,57231	0,004288
POPKDL_0:	frr	ribosome re	J	0,35037	0,782794	-4,11916	0,200876
POPKDL_0:	pyrH	UMP kinase	F	0,954825	0,50501	0,965967	0,703629
POPKDL_0:	POPKDL_0:	hypothetica	J	2,17765	0,120827	1,537513	0,499118
POPKDL_0:	rplA	50S ribosor	J	5,405496	0,059572	6,580823	0,018821
POPKDL_0:	rplK	50S ribosor	J	4,363041	0,14123		
POPKDL_0:	POPKDL_0:	DUF3397 d	S	-2,45443	2,65E-09	-2,11717	0,008533
POPKDL_0:	POPKDL_0:	cell divisior	D	-0,34358	0,817109	2,083953	0,084142
POPKDL_0:	POPKDL_0:	hypothetica	O	0,751044	0,186702	1,126769	0,089948
POPKDL_0:	POPKDL_0:	peptidylprolyl isomeras		0,566332	0,340967	0,452022	0,710633
POPKDL_0:	znuB	manganese	P	-3,1377	6,47E-10	-2,63932	0,006373
POPKDL_0:	POPKDL_0:	manganese	P	6,37591	0,003982	5,482926	0,044581
POPKDL_0:	mtsA	Metal ABC	P	-5,04686	6,49E-20	-3,13446	0,008935
POPKDL_0:	Spy490380c			-0,04281	0,799056	-2,69135	0,672526
POPKDL_0:	POPKDL_0:	MarR family	K	0,277362	0,588619	0,775657	0,540976
POPKDL_0:	mtnN	5'-methylth	E	0,827611	0,622888	4,111069	0,006606
POPKDL_0:	macP	cell wall sy	M	5,004254	0,033648	2,677765	0,675805
POPKDL_0:	POPKDL_0:	ADP-ribose	L	-5,4798	5,81E-24	-5,78012	5,09E-16
POPKDL_0:	glmU	bifunctiona	M	2,747379	0,003492	2,563768	0,069225

POPKDL_0:	POPKDL_0:	Scyllo-inosi	S	1,189742	0,069517	2,307196	0,037809
POPKDL_0:	POPKDL_0:	RNA-bindin	P	1,007265	0,046934	0,367602	0,737516
POPKDL_0:	fabG	3-oxoacyl-/	I Q	0,817169	0,017508	1,378275	0,021539
POPKDL_0:	POPKDL_0:	DUF2829 domain-containing protein				6,390996	0,02438
POPKDL_0:	POPKDL_0:	hypothetica	S	-4,02561	2,20E-22	-3,11238	5,69E-06
POPKDL_0:	brnQ	branched-c	E	0,531537	0,077022	0,374593	0,617234
POPKDL_0:	nrdE	class 1b rib	F	0,843982	0,0087	1,19894	0,028651
POPKDL_0:	nrdI	class Ib ribo	F	0,602036	0,069139	0,264025	0,74691
POPKDL_0:	nrdF	class 1b rib	F	0,617114	0,054993	1,162529	0,03169
POPKDL_0:	metG	methionine	J	0,38305	0,32773	-0,12771	0,903333
POPKDL_0:	POPKDL_0:	YoaK family	S	0,84838	0,014078	0,738358	0,319001
POPKDL_0:	POPKDL_0:	Putative me	S	-1,9493	7,77E-12	-1,18481	0,094778
POPKDL_0:	POPKDL_0:	peptidase S	O	-1,31243	5,47E-06	-1,3966	0,002091
POPKDL_0:	lctO	L-lactate ox	C	0,702723	0,156643	0,945469	0,330207
POPKDL_0:	POPKDL_0:	lactate perr	C	1,870134	1,10E-07	1,778107	0,010131
POPKDL_0:	xth	exodeoxyril	L	0,092218	0,855006	-1,36433	0,322737
POPKDL_0:	POPKDL_0:	HAD family	S	-2,59278	5,82E-14	-1,25782	0,112928
POPKDL_0:	POPKDL_0:	LacI family	K	1,115031	0,00578	1,722116	0,002587
POPKDL_0:	POPKDL_0:	VOC family	E	0,540528	0,192398	0,17189	0,898317
POPKDL_0:	POPKDL_0:	VOC family	S	1,344964	0,133522	2,351797	0,072365
POPKDL_0:	arsC	arsenate re	P	1,061498	0,00086	0,897414	0,238475
POPKDL_0:	POPKDL_0:	cysteine m	L	2,601619	2,29E-10	2,6157	0,000909
POPKDL_0:	POPKDL_0:	GNAT famil	M	0,841923	0,005659	0,98544	0,073882
POPKDL_0:	POPKDL_0:	PF03932 fa	P	0,177656	0,756058	1,49892	0,097071
POPKDL_0:	POPKDL_0:	DUF5684 d	S	-0,60351	0,057586	0,338405	0,684101
POPKDL_0:	rsmI	16S rRNA (c	H	-2,72655	1,20E-07	-2,32593	0,001764
POPKDL_0:	yabA	DNA replica	L	-2,68806	4,40E-07	-8,12629	5,10E-06
POPKDL_0:	POPKDL_0:	signal pepti	S	1,703058	3,19E-05	2,318033	9,49E-05
POPKDL_0:	holB	DNA polym	L	2,132402	0,058597	0,054035	1
POPKDL_0:	tmk	dTMP kinas	F	0,747048	0,364987	0,144243	1
POPKDL_0:	POPKDL_0:	CBS domai	S	1,630305	0,005057	2,066263	0,045821

POPKDL_0:	POPKDL_0:	DUF2129 d	S	-2,32513	4,37E-07	-3,66951	0,001875
POPKDL_0:	POPKDL_0:	Control of c	S	0,871485	0,001901	1,699943	0,004633
POPKDL_0:	clpP	ATP-depend	O U	2,388306	0,058244	0,96942	0,605818
POPKDL_0:	upp	uracil phos	F	-0,18917	0,743712	0,153839	0,927202
POPKDL_0:	spoVB	polysaccha	S	2,36124	0,001732	2,936437	0,02312
POPKDL_0:	murE	UDP-N-ace	M	0,632034	0,426646	1,273213	0,248974
POPKDL_0:	POPKDL_0:	iron ABC tra	H P	0,094794	0,794383	0,660869	0,18739
POPKDL_0:	POPKDL_0:	Iron(3+)-hy	P	0,133955	0,683547	0,381355	0,502767
POPKDL_0:	POPKDL_0:	iron ABC tra	P	0,542584	0,077019	0,646416	0,266866
POPKDL_0:	POPKDL_0:	ferrichrome	P	0,253342	0,660412	0,257133	0,795658
POPKDL_0:	POPKDL_0:	Hypothetica	S	-0,37086	0,258424	-0,50482	0,41115
POPKDL_0:	ppaC	manganese	C	-1,10274	0,898278	1,526424	0,229091
POPKDL_0:	pflA	pyruvate fo	C	0,201772	0,772998	1,052901	0,302652
POPKDL_0:	tlyC	HlyC/CorC	S	-4,68651	3,03E-46	-4,82664	3,46E-14
POPKDL_0:	mraW	SAM-depen	J	0,525804	0,250432	1,748564	0,095583
POPKDL_0:	yhcC	TIGR01212	S	0,622159	0,069139	0,512058	0,519127
POPKDL_0:	POPKDL_0:	Undecapre	I	-2,02718	5,42E-09	-2,67696	0,000232
POPKDL_0:	POPKDL_0:	ECF transporter S comp		-1,57546	0,019054	-1,99964	0,039037
POPKDL_0:	POPKDL_0:	Clostridial hydrophobic		1,483566	8,11E-05	1,676214	0,013389
POPKDL_0:	POPKDL_0:	hypothetica	O	2,040445	1		
POPKDL_0:	trmL	tRNA (uridir	J	0,886224	0,002803	0,810116	0,214864
POPKDL_0:	yidD	membrane	S	1,507607	0,00049	1,02788	0,279232
POPKDL_0:	rsuA	pseudourid	J	1,760537	5,82E-07	2,545902	2,16E-05
POPKDL_0:	scpB	SMC-Scp co	D	-4,79184	5,68E-14	-5,33984	6,35E-11
POPKDL_0:	scpA	segregation	D	1,651634	0,127446	2,511216	0,102703
POPKDL_0:	xerD	site-specifi	L	-0,96219	0,001804	-0,16664	0,822338
POPKDL_0:	ytoI	CBS domai	S	-0,23592	0,622144	-1,2744	0,189224
POPKDL_0:	POPKDL_0:	YfcE family	S	0,315743	0,32285	-0,2368	0,805861
POPKDL_0:	rdgB	nucleoside	F	-0,11075	0,815673	-0,94379	0,325409
POPKDL_0:	racE	glutamate r	M	2,165511	0,063892	4,461849	0,00868
POPKDL_0:	yneF	UPF0154 pr	S	-0,71332	0,92756	-4,82253	0,051886

POPKDL_0:	POPKDL_0:	BAX inhibit	S	-0,04858	0,902654	0,613757	0,417324
POPKDL_0:	rnaY	HAD family	S	3,742268	3,44E-05	3,818852	0,027411
POPKDL_0:	spoU	23S rRNA m	J	0,426386	0,430937	0,351873	0,777537
POPKDL_0:	POPKDL_0:	hypothetica	C O	1,171652	0,048217	-0,10612	0,983117
POPKDL_0:	acyP	acylphosph	C	0,292322	0,47532	1,629912	0,032981
POPKDL_0:	yidC	membrane	U	-0,91497	0,084386	-0,30099	0,831653
POPKDL_0:	greA	transcriptic	K	-7,98059	7,23E-27	-7,40166	2,22E-09
POPKDL_0:	POPKDL_0:	Endolytic m	S	-0,83861	0,039028	-0,73504	0,499118
POPKDL_0:	POPKDL_0:	GNAT famil	K	-1,90409	6,78E-09	-1,83941	0,008816
POPKDL_0:	murC	UDP-N-ace	M	-0,06482	1	0,502958	0,902747
POPKDL_0:	POPKDL_0:	hypothetical protein		-0,05405	1	1,79019	0,259545
POPKDL_0:	POPKDL_0:	RNA helica:	L	-1,12128	0,006577	-0,93608	0,095056
POPKDL_0:	der	ribosome b	S	-0,17386	0,87374	0,095141	1
POPKDL_0:	POPKDL_0:	hypothetica	E	1,520878	0,000246	1,783975	0,045233
POPKDL_0:	POPKDL_0:	sugar transporter		1,915822	0,01424	2,781682	0,051886
POPKDL_0:	galE	UDP-glucos	M	2,031466	0,012388	1,875358	0,200876
POPKDL_0:	POPKDL_0:	phosphogly	M	0,094399	0,795035	0,23828	0,742113
POPKDL_0:	POPKDL_0:	DUF2304 d	M	1,341724	0,099226	-0,63464	0,833873
POPKDL_0:	POPKDL_0:	glycosyl tra	M	0,666058	0,208783	-0,46172	0,58283
POPKDL_0:	POPKDL_0:	Glyco-trans	M	0,292005	0,571235	-1,10112	0,214864
POPKDL_0:	POPKDL_0:	DUF2142 d	M	-0,71276	0,226437	-2,73497	0,008256
POPKDL_0:	POPKDL_0:	rhamnosylt	M	-1,17912	0,509032	-0,8834	0,84962
POPKDL_0:	POPKDL_0:	glycosyltrar	M	-0,45255	0,886794	0,775278	0,621417
POPKDL_0:	POPKDL_0:	glycosyltrar	M	-0,64695	0,444076	1,053243	0,538109
POPKDL_0:	POPKDL_0:	phosphate/	P M	0,742829	0,403976	-0,66142	0,861724
POPKDL_0:	POPKDL_0:	LPS ABC tra	P M	1,826894	0,028102	1,664907	0,27082
POPKDL_0:	wcaA	glycosyltrar	M	1,464876	0,080691	3,284637	0,043269
POPKDL_0:	rfaB	glycosyl tra	M	0,956697	0,17307	2,138374	0,206116
POPKDL_0:	rfbD	dTDP-4-del	M	0,611937	0,712091	1,446416	0,470918
POPKDL_0:	paaD	aromatic rii	K	-3,46959	3,56E-27	-3,68975	2,13E-10
POPKDL_0:	rpoD	RNA polym	K	2,217189	0,015265	1,901624	0,236538

POPKDL_02	POPKDL_02	DNA primas	L	1,723277	0,134429	1,758294	0,166963
POPKDL_02	mscL	large condu	M	-0,46355	0,396035	0,602519	0,694076
POPKDL_02	rpsU	30S ribosor	J	-0,12791	0,885464	-3,57512	0,437463
POPKDL_02	POPKDL_02	amino acid	E T	-1,14828	0,000861	-0,73518	0,281651
POPKDL_02	POPKDL_02	aminotrans	E	1,16453	3,12E-05	1,207021	0,014199
POPKDL_02	addA	helicase-ex	L	0,417729	0,462195	0,737218	0,666615
POPKDL_02	rexB	ATP-depend	L	1,639616	0,002719	0,692757	0,664711
POPKDL_02	POPKDL_02	hypothetica	S	1,318161	0,225578	1,180477	0,540972
POPKDL_02	POPKDL_02	Putative ne	S	-1,26929	0,00022	-0,8862	0,227557
POPKDL_02	POPKDL_02	Putative ne	S	1,729571	0,06956	2,065668	0,167003
POPKDL_02	POPKDL_02	peroxiredox	O	-0,39327	0,476922	-1,33711	0,08042
POPKDL_02	pheT	phenylalan	J	0,46466	0,486181	0,166682	0,940809
POPKDL_02	POPKDL_02	hypothetical protein		-1,88237	0,063217	-0,46483	0,848163
POPKDL_02	pheS	phenylalan	J	-0,19756	0,934684	2,95165	0,026265
POPKDL_02	POPKDL_02	DNA-entry r	F	0,881055	0,008804	0,454335	0,603253
POPKDL_02	POPKDL_02	DNA-direct	S	0,664688	1	-3,57512	0,437463
POPKDL_02	murA	UDP-N-ace	M	0,521832	0,433378	1,274273	0,383605
POPKDL_02	POPKDL_02	DUF1146 d	S	1,112549	0,00017	0,994493	0,096085
POPKDL_02	atpC	F0F1 ATP sy	C	0,805671	0,335609	1,456075	0,41368
POPKDL_02	atpD	F0F1 ATP sy	C	0,812578	0,298696	0,446018	0,843254
POPKDL_02	atpG	F0F1 ATP sy	C	2,905288	0,039455	4,028933	0,013837
POPKDL_02	atpA	F0F1 ATP sy	C	0,924107	0,408002	1,462643	0,343913
POPKDL_02	atpH	F0F1 ATP sy	C	3,247994	0,002589	0,96914	0,605818
POPKDL_02	atpF	F0F1 ATP sy	C	1,518907	0,227546	-2,69135	0,672526
POPKDL_02	atpB	F0F1 ATP sy	C	2,011145	0,00991	0,172808	1
POPKDL_02	atpE	F0F1 ATP sy	C	0,765121	0,749714	-4,51329	0,097049
POPKDL_02	glgA	glycogen sy	G	0,497139	0,183448	0,954209	0,1133
POPKDL_02	glgD	glucose-1-p	G	0,669101	0,050236	0,599847	0,431354
POPKDL_02	POPKDL_02	Glucose-1-	H	1,206405	0,003608	1,801871	5,27E-05
POPKDL_02	glgB	1,4-alpha-g	G	1,345725	0,000517	1,256258	0,031025
POPKDL_02	pulA	type I pullul	G	0,181915	0,571235	0,04285	0,973146

POPKDL_02	POPKDL_02	diacylglyce	I	1,13783	0,327716	2,826504	0,126145
POPKDL_02	ligA	NAD-deper	L	0,290039	0,610804	1,005003	0,365601
POPKDL_02	POPKDL_02	Queuosine transporter		0,737581	0,024963	0,584928	0,41368
POPKDL_02	POPKDL_02	nuclease	N	-1,18763	1,63E-05	-1,31147	0,019525
POPKDL_02	POPKDL_02	ABC transp	V	0,625541	0,517463	-0,35976	0,888555
POPKDL_02	POPKDL_02	SagG family	V	-0,45858	0,711276	0,612258	0,74476
POPKDL_02	POPKDL_02	ABC transp	V	0,347266	0,674498	-0,02641	1
POPKDL_02	POPKDL_02	SagF family	S	-2,30534	1,02E-14	-2,83692	2,64E-07
POPKDL_02	POPKDL_02	CPBP famil	S	-0,50506	0,167229	-0,63764	0,494264
POPKDL_02	POPKDL_02	streptolysir	S	0,502344	0,090216	0,329089	0,60467
POPKDL_02	POPKDL_02	streptolysir	S	1,645368	2,75E-05	2,271716	2,09E-05
POPKDL_02	POPKDL_02	streptolysir	C	1,35829	3,41E-06	1,301162	0,019615
POPKDL_02	sagA			0,093447	0,840232	-3,6547	0,00647
POPKDL_02	sagA	TOMM fami	S	0,901899	0,229938	2,420653	0,051473
POPKDL_02	eno	phosphopy	G	3,652766	3,63E-05	3,186175	0,058582
POPKDL_02	POPKDL_02	DUF1694 d	S	0,676372	0,12803	1,186543	0,193259
POPKDL_02	POPKDL_02	Integrase ca	L	-5,43998	5,04E-50	-8,01394	3,48E-21
POPKDL_02	ezrA	septation ri	D	1,589357	0,035019	3,398079	0,021496
POPKDL_02	gyrB	DNA topois	L	2,939592	1,32E-06	1,063233	0,470918
POPKDL_02	POPKDL_02	DNA gyrase	S	-3,49164	2,82E-11	-10,8965	2,16E-14
POPKDL_02	POPKDL_02	Peptidoglyc	D	0,079218	0,887236	1,050779	0,110805
POPKDL_02	Arg_trna			1,438572	0,326777	-2,69135	0,672526
POPKDL_02	rplS	50S ribosor	J	5,435768	0,014092	2,502932	1
POPKDL_02	POPKDL_02	hypothetica	L	0,235415	0,58382	0,168655	0,854222
POPKDL_02	POPKDL_02	UPF0236 fa	S	0,514059	0,172229	0,332682	0,665267
POPKDL_02	POPKDL_02	hypothetical protein		-2,26394	1,45E-11	-2,85407	3,01E-05
POPKDL_02	POPKDL_02	hypothetical protein		0,970174	0,022305	0,587888	0,565832
POPKDL_02	arcC	carbamate	E	0,669153	0,076846	0,061157	0,99132
POPKDL_02	asnA	aspartate--	E	-4,50703	8,25E-43	-4,30522	1,27E-14
POPKDL_02	rsmD	16S rRNA (g	L	-3,97383	9,41E-17	-1,73665	0,181176
POPKDL_02	coaD	pantetheine	H	0,978079	0,365642	5,053028	0,001422

POPKDL_02	POPKDL_02	peptidase S	T	0,697927	0,19435	0,127673	0,922213
POPKDL_02	POPKDL_02	LacI family	K	-3,45938	2,20E-26	-3,54	2,62E-13
POPKDL_02	rbsK	ribokinase	H	-0,90456	0,033304	-0,65498	0,384487
POPKDL_02	rbsD	D-ribose py	G	-0,24698	0,454807	-0,71524	0,323986
POPKDL_02	ccmA	heme ABC	G	-1,79042	8,47E-11	-1,90165	0,000235
POPKDL_02	rbsC	ribose ABC	G	-4,06126	2,74E-43	-4,34903	1,48E-14
POPKDL_02	POPKDL_02	D-ribose AE	G	-1,53603	4,90E-08	-1,8837	0,001934
POPKDL_02	POPKDL_02	DUF1027 d	J	1,128421	0,010235	0,105459	0,951419
POPKDL_02	rlmN	23S rRNA (a	J	-2,38876	1,14E-11	-2,537	3,53E-05
POPKDL_02	POPKDL_02	VanZ family	V	0,034837	0,931068	-0,31885	0,621417
POPKDL_02	POPKDL_02	multidrug A	V	-0,77382	0,016332	-1,07245	0,08449
POPKDL_02	POPKDL_02	multidrug A	V	-0,90684	0,001379	-0,93537	0,110882
POPKDL_02	POPKDL_02	ABC transp	V	-1,09991	0,000107	-1,13743	0,030501
POPKDL_02	POPKDL_02	ABC transp	V	-0,55945	0,075801	-1,11513	0,059333
POPKDL_02	POPKDL_02	sensor histi	T	-1,07559	0,000786	-0,04366	0,974725
POPKDL_02	POPKDL_02	DNA-bindin	K	0,731364	0,01665	0,724553	0,200876
POPKDL_02	POPKDL_02	Leader pep	N O U	-6,39801	8,32E-35	-4,80486	5,54E-12
POPKDL_02	dps	DNA starva	P	-0,85471	0,100206	-0,53374	0,699437
POPKDL_02	yqgQ	DUF910 do	S	1,220144	0,113209	-1,22921	0,578991
POPKDL_02	nagC	glucokinase	G	1,113483	0,043366	0,540201	0,446417
POPKDL_02	pspE	rhodanese-	P	-0,05169	0,888583	0,167174	0,8363
POPKDL_02	typA	translationa	T	0,873147	0,147916	1,527595	0,21827
POPKDL_02	POPKDL_02	DUF3165 d	S	-9,9624	4,22E-23	-5,6898	2,80E-06
POPKDL_02	POPKDL_02	ribonuclease		-1,25356	7,04E-06	-0,63786	0,305453
POPKDL_02	murD	UDP-N-ace	M	2,674422	0,000164	1,312975	0,338432
POPKDL_02	murG	UDP-N-ace	M	2,965788	0,000706	2,155255	0,238852
POPKDL_02	POPKDL_02	cell divisior	D	0,719831	0,337508	1,059927	0,540976
POPKDL_02	ftsA	cell divisior	D	1,954356	0,003294	2,240644	0,076148
POPKDL_02	ftsZ	cell divisior	D	1,301482	0,140275	2,608762	0,023966
POPKDL_02	POPKDL_02	YggS family	S	1,630158	0,008164	1,091002	0,364249
POPKDL_02	POPKDL_02	cell divisior	D	-7,1427	2,60E-24	-4,90699	1,71E-06

POPKDL_02	POPKDL_02	YggT family	D	0,002914	0,787655	-1,78006	0,631588
POPKDL_02	POPKDL_02	RNA bindin	S	0,03372	0,815673	-5,29331	0,076752
POPKDL_02	divIVA	cell divisior	D	1,733567	0,102158	2,994496	0,023847
POPKDL_02	ileS	Isoleucine-	J	1,530214	0,009786	2,50316	0,037527
POPKDL_02	POPKDL_02	ribose-5-ph	S	1,427367	1,67E-06	3,716773	7,29E-05
POPKDL_02	POPKDL_02	NUDIX hydr	L	-2,15003	4,99E-13	-1,60332	0,00174
POPKDL_02	clpA	ATP-depen	O	-1,13562	0,000144	-1,09922	0,039295
POPKDL_02	ykuJ	DUF1797 d	S	3,531175	0,002334	4,582309	0,004699
POPKDL_02	hisM	amino acid	P	-3,55534	5,80E-20	-2,75764	0,000187
POPKDL_02	ehuAglnQ	glutamine A	E	-4,84806	4,73E-53	-4,28513	4,27E-09
POPKDL_02	POPKDL_02	ferrous iron	P	-1,39337	9,41E-06	-2,0773	0,000441
POPKDL_02	POPKDL_02	transcriptic	K	-1,19249	6,91E-05	-1,37959	0,006657
POPKDL_02	POPKDL_02	Sulfite expo	S	0,279281	0,385719	1,640945	0,027023
POPKDL_02	POPKDL_02	ornithine cy	E	0,13767	0,694484	0,524148	0,378355
POPKDL_02	POPKDL_02	phosphoglu	G	0,602356	0,090216	0,691353	0,205079
POPKDL_02	POPKDL_02	bifunctiona	F	-0,62755	0,417083	-2,19018	0,195665
POPKDL_02	POPKDL_02	carbohydra	H	1,13965	0,000119	1,312008	0,007272
POPKDL_02	xseA	exodeoxyrit	L	0,375216	0,332679	2,160622	0,025557
POPKDL_02	xseB	exodeoxyrit	L	0,433668	0,743712	-0,82495	0,553442
POPKDL_02	POPKDL_02	polyprenyl s	H	-0,40608	0,39426	-0,19418	0,861729
POPKDL_02	yqxC	TlyA family	J	-1,52918	0,033818	-1,44187	0,10162
POPKDL_02	POPKDL_02	ArgR family	K	0,757723	0,019144	0,808715	0,162856
POPKDL_02	recN	DNA repair	L	-1,14894	0,000643	-0,67747	0,2983
POPKDL_02	POPKDL_02	GRAM-POS-ANCHORIN		0,647246	0,032162	0,406232	0,558042
POPKDL_02	POPKDL_02	DEDD-Tnp-	X	-0,172	0,606661	-0,01108	1
POPKDL_02	degV	DegV doma	T	-1,99783	1,09E-05	-2,2068	0,004903
POPKDL_02	POPKDL_02	Spore germ	E	-1,22628	0,000314	-1,42588	0,019822
POPKDL_02	POPKDL_02	DUF2140 d	S	-1,91529	1,54E-06	-3,60005	3,71E-07
POPKDL_02	hup	DNA-bindir	L	4,712363	0,095774		
POPKDL_02	POPKDL_02	Major Facili	E G P	1,102487	0,003794	1,768212	0,000909
POPKDL_02	POPKDL_02	MutR family	K	0,893922	0,003362	1,086471	0,071745

POPKDL_0:	cadA	cadmium-t	P	1,154659	0,000152	0,953142	0,064282
POPKDL_0:	pyrD	dihydroorot	F	0,22491	0,73915	-1,12861	0,125559
POPKDL_0:	gpmA	phosphogly	G	3,101195	0,001317	3,07035	0,038394
POPKDL_0:	POPKDL_0:	transcriptic	S	-6,69646	4,95E-32	-6,00342	3,69E-09
POPKDL_0:	POPKDL_0:	Membrane protein		-0,62801	0,038743	-0,4625	0,420668
POPKDL_0:	focA	formate-nit	P	1,23212	0,001121	0,97605	0,204103
POPKDL_0:	pbp2b	penicillin-b	M	-0,53028	0,102368	-0,67936	0,229022
POPKDL_0:	recR	recombination mediatc		5,910483	0,001743	5,985589	0,037916
POPKDL_0:	POPKDL_0:	D-alanine--	L	2,821632	0,000295	3,174484	0,046598
POPKDL_0:	murF	UDP-N-ace	F	0,069108	0,899112	-0,39935	0,799583
POPKDL_0:	POPKDL_0:	TIGR02206	M	2,033617	3,69E-07	2,571694	1,49E-07
POPKDL_0:	POPKDL_0:	hypothetica	S	-11,6495	2,10E-41	-11,0078	5,65E-15
POPKDL_0:	prfC	peptide cha	J	-1,61639	6,02E-07	-2,66241	0,000439
POPKDL_0:	POPKDL_0:	helicase	S	-0,29892	0,448393	-0,6613	0,532428
POPKDL_0:	pepT	peptidase T	E	2,121455	2,33E-07	3,891013	3,09E-06
POPKDL_0:	POPKDL_0:	Pore formir	S	2,115592	2,27E-11	2,315021	5,63E-06
POPKDL_0:	POPKDL_0:	LysM peptic	M	1,880364	5,13E-10	2,477408	2,16E-05
POPKDL_0:	cmk	(d)CMP kin:	F	-1,26636	1	3,064247	0,097049
POPKDL_0:	infC	translation	J	3,081588	0,00312	0,05307	1
POPKDL_0:	rpmI	50S ribosor	J	2,793151	0,622144	4,67165	0,21631
POPKDL_0:	rplT	50S ribosor	J	1,791718	0,150115	-0,84102	1
POPKDL_0:	mdoB	alkaline ph	M	2,737663	2,47E-06	2,020017	0,106515
POPKDL_0:	rlmK	23S rRNA G	J	0,050829	0,934684	-0,34065	0,819673
POPKDL_0:	aroD	3-dehydroq	E	1,991116	0,044271	-3,57512	0,437463
POPKDL_0:	aroC	chorismate	E	0,62426	0,169188	0,888337	0,322737
POPKDL_0:	POPKDL_0:	YlbF/YmcA	S	1,632366	0,028154	2,752601	0,035696
POPKDL_0:	gorA	glutathione	C	-0,09156	0,815673	-0,24673	0,754073
POPKDL_0:	folC	dihydrofola	H	0,717988	0,075379	0,582618	0,437463
POPKDL_0:	POPKDL_0:	hypothetica	S	0,622478	0,110014	0,551737	0,615121
POPKDL_0:	POPKDL_0:	Cysteine de	E	-2,93472	5,14E-21	-2,3754	9,30E-06
POPKDL_0:	thiI	putative tRI	H	-1,00299	0,022315	-1,75217	0,003087

POPKDL_02	capA	Poly-gamm	M	-2,78305	1,43E-13	-3,07636	3,05E-07
POPKDL_02	rplU	50S ribosor	J	4,823152	0,08189	2,770064	0,675805
POPKDL_02	ysxB	ribosomal-|	J	4,798779	0,063892	6,339179	0,026276
POPKDL_02	rpmA	50S ribosor	J	-1,67537	0,743712	-4,11916	0,200876
POPKDL_02	lysR	LysR family	K	0,063146	0,996718	-4,42023	4,27E-05
POPKDL_02	lspA	signal pepti	M U	-0,09624	0,855382	-0,28182	0,779791
POPKDL_02	rluA	RluA family	J	-2,56848	9,62E-09	-3,25351	0,006852
POPKDL_02	POPKDL_02	TVP38/TME	S	-0,77833	0,072387	-0,22574	0,828906
POPKDL_02	pyrR	bifunctiona	F	2,045227	0,150971	3,517461	0,023581
POPKDL_02	uraA	uracil perm	F	-3,45907	1,09E-07	-2,83725	0,014724
POPKDL_02	pyrB	aspartate c	F	-0,31756	0,657379	-2,55312	0,000152
POPKDL_02	carA	carbamoyl	F	0,129482	0,882521	-2,46997	0,003122
POPKDL_02	carB	carbamoyl-	F	-2,85016	1,48E-07	-3,69677	1,89E-08
POPKDL_02	POPKDL_02	efflux trans	M	-0,41962	0,181494	-0,06956	0,9267
POPKDL_02	POPKDL_02	macrolide A	V	1,437702	0,000654	1,885398	0,028059
POPKDL_02	POPKDL_02	MacB-like p	S	0,282545	0,467813	0,135891	0,872211
POPKDL_02	POPKDL_02	SNARE ass	C	2,213356	2,02E-12	1,893306	0,004138
POPKDL_02	POPKDL_02	glycerophos	J	-0,23071	0,470257	-0,18447	0,799583
POPKDL_02	rpsP	30S ribosor	J	5,185105	0,022901	2,679558	0,675805
POPKDL_02	ylqC	RNA-bindin	L	5,833262	0,004337	5,190333	0,090054
POPKDL_02	rimM	ribosome m	J	0,039584	1	-1,85041	0,437463
POPKDL_02	trmD	tRNA (guan	J	1,22656	0,377302	0,731811	0,736363
POPKDL_02	trxB	Ferredoxin-	C	-3,33123	2,89E-10	-4,85446	1,32E-06
POPKDL_03	pfoR	Phosphotra	S	-1,5529	6,29E-08	-1,26551	0,009731
POPKDL_03	apbA	2-dehydrop	H	0,320604	0,316227	0,068351	0,940809
POPKDL_03	POPKDL_03	DeoR family	K	-1,61848	1,19E-06	-0,58322	0,350692
POPKDL_03	pfkB	1-phosphof	H	-0,77655	0,011359	-0,58005	0,3305
POPKDL_03	ptsN	PTS fructos	G	0,145236	0,653555	0,309627	0,641328
POPKDL_03	POPKDL_03	N-acetylmu	N U	0,245405	0,643769	0,100528	0,984973
POPKDL_03	POPKDL_03	N-acetylmu	N U	-6,3955	2,21E-27	-6,50531	1,41E-19
POPKDL_03	POPKDL_03	hypothetical protein		0,949343	0,001342	3,527009	0,000193

POPKDL_0:	POPKDL_0:	Transposas	X	0,828191	0,457615	-0,91258	1
POPKDL_0:	POPKDL_0:	EDD domai	S	-0,10597	0,824485	-0,32544	0,778667
POPKDL_0:	cca	CCA tRNA r	J	0,354807	0,69196	3,004508	0,055696
POPKDL_0:	abcf	ABC-F type	S	-2,08792	3,14E-09	-2,40297	2,94E-05
POPKDL_0:	gdhA	NADP-spec	E	1,320435	0,004769	1,566604	0,002649
POPKDL_0:	POPKDL_0:	peptide def	J	0,122878	0,879541	0,569665	0,563408
POPKDL_0:	POPKDL_0:	bifunctiona	F	0,184774	0,709825	0,976481	0,195665
POPKDL_0:	tetA46	ABC transp	V	0,625147	0,074202	0,311817	0,685338
POPKDL_0:	tetB46	ABC transp	V	-0,52296	0,077583	-0,41176	0,499118
POPKDL_0:	mvk	mevalonate	I	0,86803	0,442224	-4,51329	0,097049
POPKDL_0:	mvaD	diphosphor	I	-0,31325	0,797663	-5,48135	0,04973
POPKDL_0:	eRG12	phosphome	I	2,593624	0,001326	4,249395	0,013389
POPKDL_0:	fni	type 2 isop	C	1,290559	0,148022	0,237677	0,933511
POPKDL_0:	POPKDL_0:	hydroxymet	C	1,505809	0,011141	0,191043	0,97778
POPKDL_0:	POPKDL_0:	hydroxymet	I	1,917394	0,043869	0,380768	0,921073
POPKDL_0:	POPKDL_0:	thymidylate	F	1,812493	0,068342	2,055176	0,250118
POPKDL_0:	POPKDL_0:	Dihydrofola	H	2,680797	0,014307	-2,69135	0,672526
POPKDL_0:	POPKDL_0:	Hypothetical membran		0,061766	1	-1,389	0,862941
POPKDL_0:	clpX	ATP-depen	O	3,384856	0,004225	-2,69135	0,672526
POPKDL_0:	yihA	ribosome biogenesis G		5,185455	0,024402	5,478364	0,025624
POPKDL_0:	POPKDL_0:	Virulence fa	D	2,098902	4,19E-11	4,560741	3,11E-06
POPKDL_0:	POPKDL_0:	IS30 family transposas		1,350652	2,53E-05	1,256978	0,099901
POPKDL_0:	POPKDL_0:	HTH-38 domain-contai		-6,97098	3,91E-42	-5,02484	2,61E-16
POPKDL_0:	POPKDL_0:	ISL3 family transposas		-1,62384	6,87E-08	-1,751	0,021043
POPKDL_0:	POPKDL_0:	hypothetical protein		0,919216	0,005119	0,510118	0,56796
POPKDL_0:	POPKDL_0:	voltage-gat	P	-0,09104	0,811661	-0,20982	0,746429
POPKDL_0:	POPKDL_0:	chorismate	E	-0,44107	0,328399	-0,57282	0,684101
POPKDL_0:	fldA	flavodoxin	C	1,415599	0,26547	4,229247	0,016232
POPKDL_0:	add	adenosine	F	-0,09305	0,946803	0,405731	0,772253
POPKDL_0:	POPKDL_0:	Disulfide-b	H	2,987621	1,40E-12	3,751138	3,88E-12
POPKDL_0:	POPKDL_0:	Glutathione	O	-0,57788	0,098351	-0,55477	0,482305

POPKDL_03	POPKDL_03	Acetyltrans	S	0,997254	0,003224	1,02944	0,100292
POPKDL_03	nrnA	bifunctiona	S	1,482045	0,147202	-0,29701	1
POPKDL_03	rpmE2	type B 50S	J	3,094144	0,397057		
POPKDL_03	POPKDL_03	PTS fructos	G	0,691107	0,064403	2,085284	0,005892
POPKDL_03	POPKDL_03	PTS fructos	G	0,688888	0,037829	1,069925	0,038367
POPKDL_03	POPKDL_03	PTS fructos	G	2,356055	2,98E-07	2,756166	3,05E-07
POPKDL_03	POPKDL_03	PTS manno	G	-0,14574	0,751742	-0,74531	0,634516
POPKDL_03	POPKDL_03	beta-galact	G	0,742425	0,011519	1,133738	0,037775
POPKDL_03	POPKDL_03	GntR family	K	0,268555	0,566427	1,20902	0,152797
POPKDL_03	lacD	tagatose-bi	G	3,225679	0,001986	1,925789	0,288118
POPKDL_03	agaS	tagatose-6-	M	-4,47912	2,01E-25	-4,23175	1,41E-12
POPKDL_03	mngR	GntR family	K	-1,06742	0,0069	0,881582	0,332123
POPKDL_03	adcA	Zinc-bindin	P	-0,46571	0,115357	-0,69239	0,24163
POPKDL_03	pepDA	C69 family	E	-1,45021	1,70E-05	-1,73113	0,002653
POPKDL_03	whiA	DNA-bindir	K	-1,29851	0,00442	-2,17896	0,005052
POPKDL_03	cofD	Putative glu	S	0,061651	0,873385	0,216422	0,771542
POPKDL_03	rapZ	RNase adaj	S	0,78401	0,103175	0,374381	0,73742
POPKDL_03	POPKDL_03	DUF1003 d	S	0,697502	0,085971	0,694555	0,291863
POPKDL_03	asnS	asparagine	J	1,844346	0,010757	0,029957	1
POPKDL_03	POPKDL_03	aspartate a	E	1,237239	0,077508	1,076016	0,431411
POPKDL_03	POPKDL_03	bifunctiona	L	-0,22883	0,55932	-0,2392	0,754073
POPKDL_03	POPKDL_03	Putative me	S	1,849202	2,43E-05	2,232243	3,38E-05
POPKDL_03	ftsX	permease-l	D	0,206995	0,795381	1,973068	0,180141
POPKDL_03	ftsE	cell divisior	D	0,637293	0,634846	0,813723	0,771421
POPKDL_03	prfB	peptide cha	J	-0,98058	0,643939	0,852758	0,515637
POPKDL_03	queG	tRNA epoxy	C	1,754701	1,23E-07	2,100037	7,31E-05
POPKDL_03	POPKDL_03	fructose-bi	G	0,316931	0,377826	2,266322	0,009462
POPKDL_03	POPKDL_03	HAD family	S	-1,75309	3,49E-08	-1,32647	0,013243
POPKDL_03	POPKDL_03	hypothetica	S	-0,32118	0,34716	0,295754	0,617423
POPKDL_03	POPKDL_03	hypothetical protein		-2,1622	0,610007	1,113829	0,547325
POPKDL_03	POPKDL_03	3',5'-cyclic adenosine r		0,867015	0,014485	0,252076	0,870001

POPKDL_03	POPKDL_03	hypothetical protein		-2,41027	3,51E-08	-1,86504	0,081506
POPKDL_03	POPKDL_03	Hyaluronat	M	0,909912	0,001204	0,752353	0,217651
POPKDL_03	POPKDL_03	Bifunctiona	G	-0,72939	0,062193	-0,85231	0,302652
POPKDL_03	rbsK	sugar kinas	H	0,075922	0,842649	-0,16257	0,860098
POPKDL_03	rpiB	Ribose/Gal	G	1,425291	0,179817	2,27461	0,039818
POPKDL_03	fabG	gluconate 5	I Q	-0,27956	0,610007	-1,59443	0,165882
POPKDL_03	POPKDL_03	PTS N-acety	G	-2,78489	5,45E-22	-3,21941	2,44E-06
POPKDL_03	POPKDL_03	Unsaturate	S	-1,71809	9,85E-10	-0,21861	0,808328
POPKDL_03	agaB	PTS N-acety	G	-0,64301	0,025819	-0,91448	0,26613
POPKDL_03	manY	PTS manno	G	0,970996	0,000605	1,090022	0,032981
POPKDL_03	manZ	PTS N-acety	G	0,129303	0,712114	0,092464	0,907261
POPKDL_03	POPKDL_03	oligohyalur	S	0,624303	0,058985	0,507673	0,415252
POPKDL_03	purR	transcriptic	K	0,766609	0,056025	1,717303	0,020291
POPKDL_03	POPKDL_03	hypothetical protein		0,463432	0,234372	0,307107	0,860098
POPKDL_03	mgtA	calcium-tra	P	0,269224	0,616157	-0,03496	1
POPKDL_03	SSRC10			-7,7412	9,08E-10	-1,06301	0,41368
POPKDL_03	POPKDL_03	DUF1934 d	S	0,084097	0,87374	-0,33701	0,860098
POPKDL_03	ydhJ	phosphohy	S	-3,32002	5,28E-28	-3,56996	6,75E-11
POPKDL_03	yidA	sugar-phos	S	0,339594	0,881368	-0,11744	1
POPKDL_03	POPKDL_03	UDP-N-ace	M	1,106559	0,283141	2,354198	0,112928
POPKDL_03	POPKDL_03	peptidoglyc	G	2,21799	0,005327	-0,13611	1
POPKDL_03	tpiA	triose-phos	L	1,217541	0,12296	1,763656	0,256159
POPKDL_03	POPKDL_03	IS30 family	X	-0,43784	0,203006	-0,04284	0,976978
POPKDL_03	POPKDL_03	Poly-beta-1	E	0,480932	0,32088	1,352252	0,014967
POPKDL_03	POPKDL_03	peptide AB(	P	0,230096	0,58415	1,275837	0,164962
POPKDL_03	POPKDL_03	ABC transp	E P	0,318369	0,307962	1,32694	0,033527
POPKDL_03	POPKDL_03	ABC transp	P	0,385639	0,562126	1,622761	0,042049
POPKDL_03	POPKDL_03	ABC transp	P	0,910985	0,018154	0,242368	0,879274
POPKDL_03	rnj1	Ribonuclea	S	-0,13193	0,766959	1,619192	0,222384
POPKDL_03	rpoY	DNA-direct	S	-1,12426	0,743712	-1,387	0,862941
POPKDL_03	tsaB	tRNA (aden	O	-0,05803	0,946803	0,645908	0,660435

POPKDL_03	rimI	ribosomal p	K	0,248364	0,609203	-0,08868	0,976978
POPKDL_03	tsaD	tRNA (aden	O	0,634758	0,610007	0,90226	0,578842
POPKDL_03	rpsN	30S ribosor	J	-0,33018	0,342954	-0,52233	0,564759
POPKDL_03	POPKDL_03	hypothetical protein		3,252876	1,30E-11	3,538167	8,95E-10
POPKDL_03	POPKDL_03	hypothetical protein		-0,6782	0,030217	-0,39414	0,528763
POPKDL_03	POPKDL_03	hypothetical protein		-1,87847	1,48E-07	-1,46718	0,019822
POPKDL_03	yhiN	aminoacetone oxidase		-0,11753	0,721391	0,998662	0,130745
POPKDL_03	POPKDL_03	Uncharacte	K	-1,58393	0,02751	-2,09155	0,17576
POPKDL_03	dnaQ	3'-5' exonu	L	-0,79032	0,005731	-1,1726	0,039794
POPKDL_03	POPKDL_03	MerR family	K	1,353572	3,63E-05	1,909989	0,000732
POPKDL_03	ybaB	YbaB/EbfC	S	0,964875	0,002065	0,791845	0,220226
POPKDL_03	POPKDL_03	Exported protein		0,289239	0,68294	0,251661	0,817672
POPKDL_03	POPKDL_03	DNA-bindin	T	0,127313	0,722869	-0,05977	0,964987
POPKDL_03	POPKDL_03	sensor histi	T	-0,79514	0,006296	-0,63566	0,289104
POPKDL_03	POPKDL_03	transcriptio	K	-0,83357	0,085597	-0,94829	0,573704
POPKDL_03	POPKDL_03	XRE family	K	4,491773	0,140534	5,166309	0,067307
POPKDL_03	POPKDL_03	pyridoxami	S	-0,94577	0,017611	-1,33129	0,330207
POPKDL_03	POPKDL_03	Xaa-Pro dip	E	-2,21065	5,13E-10	-2,61967	1,20E-05
POPKDL_03	POPKDL_03	aquaporin	U	0,445751	0,622144	3,47708	0,024125
POPKDL_03	POPKDL_03	sodium:sol	S	-3,63144	9,60E-32	-3,67075	6,34E-13
POPKDL_03	POPKDL_03	peptidase	E	-0,49901	0,124998	-0,65363	0,335943
POPKDL_03	POPKDL_03	serine hydr	V	-4,60521	1,82E-31	-3,72274	4,32E-09
POPKDL_03	POPKDL_03	GNAT famil	S	-0,10441	0,78288	0,315704	0,675805
POPKDL_03	pflB	formate C-a	C	-2,52452	2,76E-10	-2,07087	0,000713
POPKDL_03	dinB	DNA polym	L	2,348735	4,74E-08	2,650617	7,92E-05
POPKDL_03	POPKDL_03	Putative membrane pro		0,818207	0,005022	0,912315	0,07703
POPKDL_03	POPKDL_03	ATP-depen	L	-1,54271	1,71E-07	-1,72714	0,000104
POPKDL_03	lepB	signal pepti	U	-0,1206	0,759052	0,875317	0,680542
POPKDL_03	rnhC	ribonucleas	L	0,507128	0,533791	2,668558	0,101447
POPKDL_03	POPKDL_03	Cell division protein Zap		-10,4615	8,69E-48	-11,6899	6,85E-20
POPKDL_03	POPKDL_03	colicin V pr	S	-2,33014	0,180882	1,017139	0,530043

POPKDL_0:	POPKDL_0:	endonuclea	L	-1,96896	3,06E-05	-0,99192	0,291646
POPKDL_0:	POPKDL_0:	FAD-contai	C	-1,45001	2,34E-06	-1,6976	0,001384
POPKDL_0:	trxA	thioredoxin	O	-4,27822	3,42E-05	-0,63178	0,634346
POPKDL_0:	mutY	A/G-specifi	L	0,641805	0,041612	2,024095	0,00295
POPKDL_0:	POPKDL_0:	hypothetical protein		1,078446	0,000262	1,184901	0,010483
POPKDL_0:	rpsF	30S ribosor	J	-1,25906	0,732123	3,239261	0,089137
POPKDL_0:	ssb	Single-strar	L	1,692362	0,129166	-0,91156	1
POPKDL_0:	rpsR	30S ribosor	J	6,075804	0,007021	5,413499	0,045179
POPKDL_0:	POPKDL_0:	DUF1129 d	S	-0,02596	0,947433	-0,12037	0,867356
POPKDL_0:	corA	magnesium	P	0,461793	0,622144	2,830967	0,039666
POPKDL_0:	uvrA	excinuclea:	L	-0,38056	0,649759	-0,13169	0,927117
POPKDL_0:	POPKDL_0:	bacteriocin	C O	-7,47533	1,11E-68	-6,327	3,19E-10
POPKDL_0:	POPKDL_0:	calcium-bir	L?	-0,48311	0,099461	-0,71752	0,23776
POPKDL_0:	pepP	aminopepti	E	-4,66332	2,64E-54	-5,21259	1,34E-17
POPKDL_0:	comEB	competenc	F	2,048577	0,005941	2,967681	0,012817
POPKDL_0:	efp	elongation	J	-1,23209	0,745949	0,471596	0,963992
POPKDL_0:	POPKDL_0:	Asp23/Gls2	S	1,060931	0,426708	2,319228	0,158775
POPKDL_0:	nusB	transcriptic	K	3,045452	0,383782	5,798702	0,022904
POPKDL_0:	purR	LacI family	K	-0,04442	0,902654	-0,33266	0,693709
POPKDL_0:	POPKDL_0:	sucrose-6-|	G	1,610224	0,054778	1,453422	0,213809
POPKDL_0:	nagE	PTS beta-gl	G	-0,62104	0,03978	-0,72432	0,16621
POPKDL_0:	POPKDL_0:	Endo-beta-	G	0,14549	0,65549	0,224571	0,73742
POPKDL_0:	nagC	fructokinas	G K	-1,57287	2,80E-08	-1,69872	0,003187
POPKDL_0:	manA	mannose-6	G	-0,6671	0,125859	0,067435	0,940809
POPKDL_0:	cfg	CAMP factc	N	-2,69117	4,69E-17	-2,57241	3,85E-07
POPKDL_0:	POPKDL_0:	cation-efflu	P	1,038647	0,147804	2,266375	0,016586
POPKDL_0:	alsT	sodium:ala	E	-3,9575	2,64E-08	-3,92568	2,38E-05
POPKDL_0:	pcrA	DNA helica	L	2,537542	0,000381	2,851786	0,013389
POPKDL_0:	POPKDL_0:	Transglyco:	S	0,070359	0,900195	0,131935	0,976978
POPKDL_0:	amaP	alkaline shock respons		0,576046	0,060266	0,986232	0,074195
POPKDL_0:	POPKDL_0:	DUF2273 domain-cont		0,935474	0,058501	0,433813	0,758681

POPKDL_0:	yloU	Asp23/Gls24 family env		2,337442	4,83E-05	0,924004	0,603131
POPKDL_0:	POPKDL_0:	CsbD family protein		-0,0001	1	-1,63533	0,319113
POPKDL_0:	yloU	Asp23/Gls24 family env		-1,6549	1,45E-05	-2,51256	0,001902
POPKDL_0:	khtT	GntR family	K	0,446486	0,622144	2,018225	0,302652
POPKDL_0:	POPKDL_0:	hypothetical protein		-0,43088	0,465766	-0,2984	0,953182
POPKDL_0:	POPKDL_0:	hypothetical protein		-0,01523	0,975662	1,41463	0,21631
POPKDL_0:	lolD	macrolide /	V	-0,00724	1	0,670028	0,258143
POPKDL_0:	lolE	peptide AB(	V	-0,30171	0,412491	-0,60365	0,304701
POPKDL_0:	POPKDL_0:	restriction (	V	1,529167	0,002783	1,373067	0,101558
POPKDL_0:	POPKDL_0:	Aromatic ac	E	-0,64326	0,158002	2,090909	0,074926
POPKDL_0:	POPKDL_0:	DUF2130 d	S	1,9344	6,33E-09	2,322297	9,98E-06
POPKDL_0:	truB	tRNA pseuc	J	0,726913	0,078103	0,611807	0,563188
POPKDL_0:	ribF	bifunctiona	H	-0,53053	0,661271	1,14955	0,442029
POPKDL_0:	spx	Global tran	K	3,255198	0,003172	-2,69135	0,672526
POPKDL_0:	POPKDL_0:	Uncharacte	S	0,876599	0,020337	0,792864	0,357124
POPKDL_0:	suhB	inositol mo	G	1,123107	0,001116	0,69141	0,353629
POPKDL_0:	ncl1	RNA methy	J	-2,03927	1,40E-13	-1,69117	0,000287
POPKDL_0:	pstS	phosphate-	P	0,762281	0,385622	-0,99549	0,234101
POPKDL_0:	pstC	phosphate	P	1,154109	0,13209	-0,07764	1
POPKDL_0:	pstA	phosphate	P	-0,47763	0,445097	-1,37763	0,162496
POPKDL_0:	pstB	phosphate	P	-2,04683	0,001804	-3,10856	3,61E-07
POPKDL_0:	pstB	phosphate	P	0,375545	0,63309	-1,5971	0,02372
POPKDL_0:	phoU	phosphate	P	-2,20617	0,607445	2,958054	0,066801
POPKDL_0:	POPKDL_0:	aminopepti	E	0,686251	0,025862	0,484305	0,397433
POPKDL_0:	ompR	DNA-bindir	T	-0,68168	0,216786	-0,09638	0,938074
POPKDL_0:	POPKDL_0:	sensor histi	T	1,384502	0,000181	1,852856	0,003058
POPKDL_0:	rpsT	30S ribosor	J	-4,33533	5,91E-24	-6,28382	1,00E-12
POPKDL_0:	coaA	type I pantc	F	0,747743	0,453524	-3,57512	0,437463
POPKDL_0:	POPKDL_0:	methyltran:	J	0,819453	0,007288	1,140768	0,039774
POPKDL_0:	pdpdeoA	pyrimidine-	F	1,611455	1,53E-07	2,108645	2,09E-05
POPKDL_0:	deoC	deoxyribos(	F	-1,00299	0,000784	-0,09721	0,919246

POPKDL_03	cdd	cytidine dea	F	-3,18651	9,37E-10	-3,10073	0,000466
POPKDL_03	med	BMP family	S	0,995058	0,001112	2,396998	0,000318
POPKDL_03	ccmA	heme ABC	S	1,054086	0,000435	3,2273	0,000158
POPKDL_03	nupP	branched-c	S	-0,96475	0,001246	0,56042	0,493131
POPKDL_03	nupQ	sugar ABC t	S	-3,97023	9,18E-42	-1,58567	0,097049
POPKDL_03	POPKDL_03	phosphoglu	G	0,228402	0,751003	1,043927	0,45133
POPKDL_04	POPKDL_04	pantotheni	S	-2,51375	7,07E-11	-2,81264	9,75E-05
POPKDL_04	coaC	phosphopa	H	0,543333	0,337508	-2,11357	0,182769
POPKDL_04	POPKDL_04	phosphopa	H	-3,95306	2,77E-11	-4,27178	2,36E-13
POPKDL_04	POPKDL_04	protein--pro	H	-2,41194	5,18E-18	-0,82941	0,312037
POPKDL_04	POPKDL_04	NADH-depe	C	1,113597	0,003439	1,233978	0,019839
POPKDL_04	POPKDL_04	LLM class f	C	1,224127	5,28E-05	0,917976	0,178126
POPKDL_04	gcvH	Glycine clea	E	0,519636	0,218201	0,487547	0,609021
POPKDL_04	POPKDL_04	protein-ADI	S	1,357619	5,80E-06	1,789915	0,001424
POPKDL_04	POPKDL_04	Protein ADI	K	-3,20298	4,18E-22	-3,37707	2,20E-09
POPKDL_04	lplA	Lipoate--pr	H	-1,18431	0,000316	-0,68298	0,28343
POPKDL_04	fhs1	formate--te	F	0,195214	0,684316	0,024739	0,996857
POPKDL_04	cls	cardiolipin	I	-0,97784	0,01424	-1,97173	0,001345
POPKDL_04	asd		F	-5,95876	6,65E-38	-6,72042	9,09E-13
POPKDL_04	nrdD	ATP-binding	K	1,667135	0,000547	1,705443	0,025476
POPKDL_04	POPKDL_04	PLP-dependent aminot		1,588316	2,35E-05	2,104049	0,000274
POPKDL_04	POPKDL_04	pyridoxami	H	-1,04037	0,00041	-1,29967	0,037533
POPKDL_04	POPKDL_04	ECF-type ril	S	0,553806	0,078103	0,448177	0,421857
POPKDL_04	ccmA	heme ABC	S	1,040697	0,00032	0,945956	0,055801
POPKDL_04	POPKDL_04	CsbD famil	K	-5,48635	5,26E-55	-4,75053	2,48E-16
POPKDL_04	POPKDL_04	hypothetical protein		-5,88506	1,03E-62	-4,52876	4,06E-11
POPKDL_04	POPKDL_04	Protein essC		0,270324	0,465525	2,524924	0,008481
POPKDL_04	POPKDL_04	hypothetical protein		-0,21299	0,598907	-0,46841	0,608023
POPKDL_04	POPKDL_04	DUF1492 domain-conta		-0,57222	0,205197	-1,12065	0,185471
POPKDL_04	POPKDL_04	DUF1492 domain-conta		-2,82606	8,69E-19	-2,87197	1,04E-07
POPKDL_04	POPKDL_04	hypothetical protein		2,767653	8,56E-09	3,388958	6,85E-13

POPKDL_0	POPKDL_0	hypothetical protein		0,103722	0,950507	0,389787	0,705373
POPKDL_0	tnpIS3	IS3 family t	X	-3,38607	9,81E-27	-3,48652	1,28E-08
POPKDL_0	POPKDL_0	HTH cro/C1	K	-0,7404	0,015007	-0,25935	0,693709
POPKDL_0	POPKDL_0	HTH cro/C1	K	1,272335	2,27E-06	1,325786	0,012465
POPKDL_0	POPKDL_0	Virulence p	S	-2,73634	7,13E-22	-3,42785	2,78E-06
POPKDL_0	hinT	Purine nucl	F G	-2,11415	1,11E-08	-1,83034	0,000552
POPKDL_0	znuB	zinc ABC tr	P	-2,00194	2,70E-05	-2,52641	0,015084
POPKDL_0	znuC	zinc ABC tr	P	1,970839	0,017977	1,556577	0,464523
POPKDL_0	marR	zinc-depen	K	-0,33395	1	1,765597	0,236655
POPKDL_0	ispE	4-(cytidine	F	-1,55403	4,34E-08	-1,64264	0,01152
POPKDL_0	POPKDL_0	farnesyl pyr	H	2,457962	4,22E-07	3,941158	1,05E-08
POPKDL_0	cydC	thiol reduct	V	0,482357	0,101491	0,345551	0,602004
POPKDL_0	cydD	thiol reduct	V	0,678476	0,026074	0,804685	0,157169
POPKDL_0	cydB	cytochrome	C	0,020871	0,977869	-0,19229	0,84546
POPKDL_0	POPKDL_0	cytochrome	C	-0,95842	0,00209	-0,37939	0,600698
POPKDL_0	POPKDL_0	FAD-depen	C	0,933237	0,002069	0,726032	0,211941
POPKDL_0	POPKDL_0	1,4-dihydro	H	0,530517	0,086713	0,127118	0,870001
POPKDL_0	POPKDL_0	Transposas	X	1,915139	0,133509	2,369973	0,171873
POPKDL_0	POPKDL_0	Transposas	X	1,046502	0,00216	-0,09252	0,966386
POPKDL_0	POPKDL_0	Transposas	X	1,771938	0,00635	1,101534	0,353968
POPKDL_0	tnpA	IS200/IS60	X	-5,26516	4,30E-45	-6,41206	5,65E-15
POPKDL_0	POPKDL_0	Phosphogly	S	0,367568	0,755572	-0,38867	0,831256
POPKDL_0	sdaAA	L-serine am	E	8,235117	1,19E-09	2,678763	0,675805
POPKDL_0	sdaAB	L-serine am	E	-4,40295	2,23E-16	-4,06747	3,74E-05
POPKDL_0	mnmA	tRNA 2-thio	J	0,988679	0,33773	0,747438	0,692941
POPKDL_0	POPKDL_0	hypothetica	U	-1,26052	0,732123	0,933176	0,78672
POPKDL_0	POPKDL_0	Nudix hydro	F	0,52129	0,088205	-0,12655	0,925666
POPKDL_0	mnmG	tRNA uridin	D	1,35069	0,085597	0,909035	0,628339
POPKDL_0	gdpP	DHH family	T	-0,30324	0,706744	0,431027	0,771542
POPKDL_0	rplI	50S ribosor	J	0,879586	0,60045	-0,50543	1
POPKDL_0	dnaB	replicative	L	-0,40425	0,504488	1,340645	0,337469

POPKDL_04	POPKDL_04	Biofilm forn	S	4,95743	0,098477		
POPKDL_04	rpsD	30S ribosor	J	4,322237	5,07E-08	0,96881	0,605818
POPKDL_04	acrR	TetR/AcrR f	K	0,767139	0,084386	0,388139	0,690239
POPKDL_04	POPKDL_04	YhgE/Pip dc	V	-1,87928	1,83E-05	-1,49738	0,074259
POPKDL_04	POPKDL_04	DUF4097 d	S	-1,72798	0,001877	-1,89002	0,007494
POPKDL_04	POPKDL_04	DUF1700 d	S	-6,01215	2,60E-73	-5,72947	8,58E-23
POPKDL_04	padR	PadR family	K	1,24706	0,305428	-1,38759	0,862941
POPKDL_04	POPKDL_04	IS982 famil	L	-5,06054	1,39E-14	-3,52402	0,000231
POPKDL_04	POPKDL_04	coenzyme /	L	0,032292	0,996718	0,336527	0,779191
POPKDL_04	POPKDL_04	hypothetica	S	2,313508	0,095774	-2,69135	0,672526
POPKDL_04	POPKDL_04	DUF368 do	S	-0,18124	0,730832	0,745789	0,602004
POPKDL_04	rpmG	50S ribosor	J	-2,20513	0,607467	-3,57512	0,437463
POPKDL_04	rpmF	50S ribosor	J	3,864818	0,163036	4,246832	0,265992
POPKDL_04	hisS	histidine--tl	J	0,025508	1	2,360705	0,06855
POPKDL_04	aspS	aspartate--'	J	1,788778	0,003866	0,749833	0,693569
POPKDL_04	yitT	YitT family p	S	1,511084	0,039268	1,79229	0,158111
POPKDL_04	yitT	Uncharacte	S	1,360635	0,002109	1,884267	0,000385
POPKDL_04	yitT	Uncharacte	S	0,852154	0,003106	0,767626	0,161375
POPKDL_04	POPKDL_04	Putative AB	S	-0,61116	0,042143	-1,01588	0,085682
POPKDL_04	argS	arginine--tF	J	2,540855	0,000117	2,753261	0,077471
POPKDL_04	argR	arginine rep	K	1,526813	8,90E-08	1,490084	0,001934
POPKDL_04	POPKDL_04	Control of c	S	0,245186	0,732003	0,987865	0,377033
POPKDL_04	mutS	DNA misma	L	-1,60558	9,31E-08	-1,58479	0,003899
POPKDL_04	Spy1786666					6,054463	0,020611
POPKDL_04	POPKDL_04	hypothetical protein		1,2594	0,000418	0,26315	0,847524
POPKDL_04	POPKDL_04	Fibronectin	D	0,311017	0,335407	2,472691	0,007272
POPKDL_04	POPKDL_04	hypothetical protein		5,531298	0,005448	6,61068	0,006657
POPKDL_04	POPKDL_04	Putative tra	K	0,143823	0,752849	-0,37971	0,693709
POPKDL_04	POPKDL_04	Prolyl-tRNA editing prot		0,189774	0,707222	-1,98561	0,364867
POPKDL_04	POPKDL_04	adhesion p	P	-1,4992	0,00099	-1,50162	0,01152
POPKDL_04	POPKDL_04	histidine tri	S	-2,45823	1,01E-15	-2,03992	8,98E-05

POPKDL_04	POPKDL_04	DUF1002 d	S	-0,00189	1	0,41539	0,520141
POPKDL_04	POPKDL_04	adhesion p	P	-1,4223	1,48E-06	-1,46089	0,00295
POPKDL_04	POPKDL_04	histidine tri	S	0,336668	0,374597	0,499039	0,41368
POPKDL_04	Spy392987			-10,2516	1,73E-25	-9,61041	5,45E-09
POPKDL_04	fmhB	UDP-N-ace	V	-1,41326	6,78E-07	-0,89867	0,12321
POPKDL_04	POPKDL_04	Transposas	L	-2,77668	0,246969	-4,11916	0,200876
POPKDL_04	guaA	glutamine-I	F	0,702936	0,426765	1,638536	0,144131
POPKDL_04	mngR	GntR family	K	0,625633	0,454011	-0,24952	0,85676
POPKDL_04	ylxM	putative DN	S	1,920662	0,172229	-0,91065	1
POPKDL_04	POPKDL_04	Signal reco	U	0,399272	0,649759	1,808223	0,207911
POPKDL_04	POPKDL_04	DNA-bindir	K	1,463641	2,54E-05	2,497315	0,000252
POPKDL_04	xerS	tyrosine rec	D	0,82788	0,317201	1,698098	0,206788
POPKDL_04	POPKDL_04	NusG-II dor	S	-1,91935	9,84E-08	-2,35207	6,79E-06
POPKDL_04	POPKDL_04	1,4-dihydro	H	-0,31143	0,379169	-0,26034	0,751763
POPKDL_04	POPKDL_04	thiamine bi	H	-2,21847	1,10E-13	-1,02593	0,09724
POPKDL_04	POPKDL_04	FMN-bindir	S	-2,66324	1,25E-15	-3,10804	2,37E-07
POPKDL_04	POPKDL_04	polyprenyl s	H	0,989016	0,010908	2,187155	0,000245
POPKDL_04	POPKDL_04	NADH dehy	C	0,715499	0,015687	4,065477	0,00031
POPKDL_04	POPKDL_04	heptapreny	S	-2,87294	2,09E-19	-2,7114	9,84E-07
POPKDL_04	POPKDL_04	histidine kii	T	-0,70273	0,024767	-0,56644	0,343188
POPKDL_04	POPKDL_04	DNA-bindir	K T	0,751519	0,024244	0,581734	0,347476
POPKDL_04	POPKDL_04	ABC transp	V	-0,21894	0,513261	-0,00549	1
POPKDL_04	POPKDL_04	ABC transp	V	-4,50393	2,84E-23	-4,37636	1,67E-12
POPKDL_04	POPKDL_04	peptidase S	M	-1,76162	1,44E-10	-2,05927	6,96E-05
POPKDL_04	POPKDL_04	PASTA dom	S	-1,09602	0,000137	-0,63459	0,241803
POPKDL_04	POPKDL_04	Phosphatid	J	0,072452	0,844792	-0,03293	0,98443
POPKDL_04	POPKDL_04	AB hydrolas	I	-1,5151	5,54E-08	-1,66737	0,000433
POPKDL_04	citC	[citrate (prc	H	-1,88926	8,22E-06	-2,81715	6,10E-05
POPKDL_04	oadA	oxaloaceta	C	-2,3176	3,60E-16	-2,09087	5,15E-05
POPKDL_04	POPKDL_04	Citrate lyas	H	-2,81477	6,87E-09	-5,69497	4,51E-06
POPKDL_04	citF	citrate lyas	H	-0,8249	0,014509	-1,12663	0,145208

POPKDL_04	citE	citrate (pro	G	-1,66636	3,38E-05	-2,37737	0,007271
POPKDL_04	citD	citrate lyas	C	-1,50371	0,0003	-3,77816	0,000209
POPKDL_04	POPKDL_04	Membrane protein		0,278771	0,60045	0,228591	0,84546
POPKDL_04	oadB	sodium ion	C	-0,18912	0,639938	0,149715	0,832292
POPKDL_04	POPKDL_04	acetyl-CoA	I	-1,39788	0,001342	-2,4493	0,043244
POPKDL_04	POPKDL_04	hypothetical protein		-2,67074	1,13E-17	-3,23019	1,92E-07
POPKDL_04	citM	citrate tran	C	-1,21663	1,97E-05	-1,54786	0,006606
POPKDL_04	gntR	GntR family	K	-0,82831	0,011609	-1,12834	0,128521
POPKDL_04	POPKDL_04	putative 2-(	F	0,256745	0,454429	0,364826	0,545003
POPKDL_04	POPKDL_04	Transition s	S	-0,12612	0,704701	-0,67344	0,301572
POPKDL_04	oadB	glutaconyl-	C	0,022402	0,960585	0,703551	0,249206
POPKDL_04	POPKDL_04	Glutaconyl-	I	2,793278	1,18E-07	3,494702	3,29E-11
POPKDL_04	POPKDL_04	hypothetical protein		-0,05158	0,929316	0,044406	0,986114
POPKDL_04	oadA	oxaloaceta	C	-1,55055	1,65E-08	-1,38422	0,003431
POPKDL_04	trmFO	methylenet	J	1,476474	3,79E-05	1,589944	0,008882
POPKDL_04	POPKDL_04	DUF3307 d	O U	-0,8478	0,045112	-0,87149	0,11491
POPKDL_04	POPKDL_04	DNA-bindir	K	2,17831	0,000375	0,446065	0,843254
POPKDL_04	topA	type I DNA	L	1,362726	0,182572	2,452172	0,031528
POPKDL_04	dprA	DNA-proce	L U	1,201526	0,000715	1,139493	0,075504
POPKDL_04	POPKDL_04	Ribonuclea	L	-2,49303	2,42E-17	-3,1452	5,46E-06
POPKDL_04	ylqF	ribosome biogenesis G		-0,46617	0,821146	2,269478	0,155804
POPKDL_04	POPKDL_04	hypothetical protein		0,751895	0,602858	-3,57512	0,437463
POPKDL_04	yqfA	hemolysin III		1,696008	0,07824	2,71063	0,088521
POPKDL_04	lCB5	diacylglycerol kinase		0,142881	0,73284	0,00043	1
POPKDL_04	Cys_trna			-4,73092	0,033648	-4,11916	0,200876
POPKDL_04	POPKDL_04	hypothetica	S	-1,88923	7,15E-11	-2,37932	3,01E-05
POPKDL_04	POPKDL_04	GNAT famil	M	0,857727	0,060781	-0,23061	0,90878
POPKDL_04	POPKDL_04	2-keto-3-de	K	1,093381	0,000125	1,336561	0,011434
POPKDL_04	POPKDL_04	Multiple an	S	-0,48554	0,199808	-0,75024	0,29494
POPKDL_04	POPKDL_04	amidohydro	K	0,18194	0,665994	0,14174	0,915955
POPKDL_04	cspA	Major cold	K	2,716721	0,06719	1,81434	0,365788

POPKDL_04	ctsR	CtsR family	O	-1,20989	0,082535	-2,21635	0,180204
POPKDL_04	POPKDL_04	chaperone	O	-0,88265	0,003486	-0,88679	0,123423
POPKDL_04	groES	co-chapero	O	3,045055	0,383782	5,448896	0,04973
POPKDL_04	groL	chaperonin	O	0,546837	0,40257	0,798649	0,646026
POPKDL_04	POPKDL_04	MutR family transcriptio		-6,80399	9,81E-06	-6,16932	0,009283
POPKDL_04	POPKDL_04	hypothetica	V	-0,19073	0,563257	-0,30351	0,655372
POPKDL_04	POPKDL_04	Major Facili	E G P	0,732713	0,014509	0,277151	0,745859
POPKDL_04	POPKDL_04	DNA misma	F	0,341412	0,265703	0,21819	0,796027
POPKDL_04	POPKDL_04	C69 family	M	0,269709	0,394431	0,21515	0,754073
POPKDL_04	POPKDL_04	SPBc2 prop	S	-0,92682	0,004337	-1,35587	0,020082
POPKDL_04	POPKDL_04	Pseudourid	J	0,750743	0,021765	0,884998	0,097737
POPKDL_04	POPKDL_04	Cytoplasmic protein		-0,60898	1	-0,84014	1
POPKDL_04	POPKDL_04	RidA family	J	-5,21928	2,63E-42	-4,48155	5,97E-12
POPKDL_04	pbp2a	penicillin-b	M	-0,7149	0,127249	-1,96782	0,000497
POPKDL_04	secE	preprotein t	U	-0,03998	0,799056	-2,69135	0,672526
POPKDL_04	nusG	transcriptio	K	2,076016	0,021414	-1,0624	0,777537
POPKDL_04	POPKDL_04	hypothetica	D	0,560398	0,235492	1,222143	0,056788
POPKDL_04	leuS	Leucine--tR	J	1,65922	0,005448	2,366857	0,061443
POPKDL_04	ulaA	PTS sugar tr	S	0,489526	0,190455	0,694559	0,195518
POPKDL_04	sgaB	PTS ascorb	G	-3,05178	5,58E-21	-3,33232	6,52E-08
POPKDL_04	ptsN	PTS ascorb	G	-0,10179	0,753938	-0,26453	0,672526
POPKDL_04	ulaD	3-keto-L-gu	G	-0,17064	0,617548	0,067329	0,929317
POPKDL_04	sgaU	xylulose 5-p	G	-1,02116	0,000393	-0,91787	0,100473
POPKDL_04	araD	L-ribulose-5	G	-0,64624	0,023328	-0,81619	0,116764
POPKDL_04	POPKDL_04	LacI family	K	0,05533	0,884387	0,249787	0,693709
POPKDL_04	POPKDL_04	hypothetica	S	0,085762	0,797225	0,056723	0,955157
POPKDL_04	POPKDL_04	dihydrofola	C H	1,726116	4,09E-06	2,225264	3,79E-05
POPKDL_04	POPKDL_04	PTS galactit	G	0,566577	0,049104	0,478184	0,451627
POPKDL_04	POPKDL_04	amidohydro	S	0,502455	0,097925	-0,06738	0,964987
POPKDL_04	POPKDL_04	carbohydra	G	-0,5067	0,108966	-0,82096	0,111617
POPKDL_04	POPKDL_04	AP endonuc	G	-0,29054	0,509032	-0,81615	0,329139

POPKDL_0‹	bglG	ascorbate 6	S	-2,93209	1,32E-15	-3,24248	2,74E-10
POPKDL_0‹	ulaG	L-ascorbate	S	0,729603	0,050134	0,530029	0,441735
POPKDL_0‹	POPKDL_0‹	diacylglyce	I	-0,58702	0,046395	-0,89638	0,135384
POPKDL_0‹	proV	glycine beta	E	-3,82675	4,72E-36	-3,96909	1,47E-10
POPKDL_0‹	proW	glycine/bet	E	2,506273	6,51E-09	3,026523	2,65E-08
POPKDL_0‹	polA	DNA polym	L	0,608058	0,274563	0,002511	1
POPKDL_0‹	POPKDL_0‹	hypothetica	S	0,751984	0,377302	0,741252	0,658976
POPKDL_0‹	yccU	CoA-bindin	S	-1,95777	8,26E-13	-2,18745	5,01E-05
POPKDL_0‹	perR	peroxide-re	P	0,08581	1	-3,72739	0,047292
POPKDL_0‹	POPKDL_0‹	Integrase ca	L	-4,73092	0,033818	-4,11916	0,200876
POPKDL_0‹	POPKDL_0‹	HTH-38 dor	S	0,626102	0,45957	0,797556	0,571435
POPKDL_0‹	6S		L	-0,73535	0,135562	-2,28771	0,08248
POPKDL_0‹	rarA	replication-	E H	-2,88483	8,77E-18	-3,38813	7,86E-07
POPKDL_0‹	pabA	type 1 gluta	E H	-0,46735	0,27864	-0,49585	0,608425
POPKDL_0‹	pabB	aminodeox	J	0,331603	0,31327	0,135573	0,875682
POPKDL_0‹	POPKDL_0‹	amidase	S	0,542251	0,058575	0,220339	0,777537
POPKDL_0‹	POPKDL_0‹	DUF3013 d	S	-4,75223	5,96E-28	-6,81396	6,92E-12
POPKDL_05	POPKDL_05	ASCH doma	J	-0,56543	0,700902	0,699779	0,633839
POPKDL_05	prmA	50S ribosor	J	0,487851	0,222513	0,31263	0,679833
POPKDL_05	POPKDL_05	16S rRNA (u	K	0,684439	0,04128	0,795282	0,177142
POPKDL_05	POPKDL_05	LacI family	G	-3,24789	1,44E-24	-3,7686	9,19E-11
POPKDL_05	nagE	PTS glucose	L	0,928927	0,001456	1,434373	0,002693
POPKDL_05	POPKDL_05	exodeoxyrib	G	0,284306	0,377302	-0,43068	0,626886
POPKDL_05	POPKDL_05	peptidase M	F	1,125835	0,000247	0,689172	0,382551
POPKDL_05	nrdI	class Ib ribo	K	0,520802	0,567936	-4,11916	0,200876
POPKDL_05	POPKDL_05	M protein tr	D	0,082044	0,870019	0,610416	0,315604
POPKDL_05	scm2	M-like prote	K T	1,973994	2,29E-05	2,352531	1,55E-06
POPKDL_05	relA	Bifunctiona	J	-0,20132	0,622144	-0,67609	0,400804
POPKDL_05	dtd	D-aminoaci	Q T	0,382617	0,35095	0,716253	0,300058
POPKDL_05	POPKDL_05	PucR family	P	1,482747	0,000694	1,676964	0,026269
POPKDL_05	malK	sugar ABC t	G	-2,08446	2,91E-09	-1,42528	0,009484

POPKDL_0!	dexB	Glucan 1,6-	G	0,466003	0,127249	0,810917	0,175961
POPKDL_0!	POPKDL_0!	amylopullu	C O	-1,81146	2,70E-10	-2,03405	0,000192
POPKDL_0!	POPKDL_0!	thiol reduct	U	2,485976	0,044693	3,009878	0,120408
POPKDL_0!	yajC	preprotein t	H	-1,94389	0,477068	-4,82253	0,051886
POPKDL_0!	uppS	isoprenyl tr	S	0,582147	0,640891	-1,67798	0,38692
POPKDL_0!	POPKDL_0!	phosphatid	M	2,077453	0,055494	2,054844	0,250277
POPKDL_0!	rseP	RIP metallo	J	-2,57602	1,86E-09	-2,25565	0,00565
POPKDL_0!	proS	proline--tRI	J	1,681323	0,048937	0,937764	0,492815
POPKDL_0!	POPKDL_0!	Prolyl-tRNA	L	-0,05962	0,899433	1,326825	0,162416
POPKDL_0!	polC	PolC-type D	K	1,170042	0,137762	2,009858	0,081655
POPKDL_0!	POPKDL_0!	MarR family	S	0,343887	0,649759	1,296949	0,365788
POPKDL_0!	POPKDL_0!	Flavin redu	J	-0,38113	0,220932	-0,36662	0,628695
POPKDL_0!	def	peptide def	K	-2,48072	0,290501	1,597949	0,221626
POPKDL_0!	POPKDL_0!	Crp/Fnr fan	P	-1,92256	8,49E-05	-5,16608	1,49E-05
POPKDL_0!	POPKDL_0!	MFS transp	J	0,811171	0,10782	1,0878	0,244629
POPKDL_0!	rpsO	30S riboson	L	1,099165	0,618424	-0,83824	1
POPKDL_0!	POPKDL_0!	N-acetylne	E	-2,69062	6,14E-18	-2,47222	2,42E-06
POPKDL_0!	POPKDL_0!	sodium:sol	G	-0,43431	0,140937	-0,32999	0,624944
POPKDL_0!	POPKDL_0!	YhcH/YjgK/	G	0,860242	0,010235	0,19986	0,838884
POPKDL_0!	POPKDL_0!	sialidase	S	1,191808	3,58E-05	1,469849	0,009207
POPKDL_0!	mviM	oxidoreduc	G K	-2,55466	7,24E-19	-2,94952	4,80E-08
POPKDL_0!	POPKDL_0!	ROK family protein		0,758582	0,01508	0,477304	0,463148
POPKDL_0!	POPKDL_0!	hypothetica	S	-1,33082	0,000111	-1,74111	0,020059
POPKDL_0!	POPKDL_0!	hypothetical protein		-4,53806	1,41E-36	-4,61799	3,23E-16
POPKDL_0!	POPKDL_0!	DNA ligase		-2,27905	2,92E-15	-2,85646	0,000776
POPKDL_0!	POPKDL_0!	DUF5592 domain-cont		-0,5129	0,149158	-1,46595	0,01648
POPKDL_0!	POPKDL_0!	hypothetical protein		0,11002	0,813989	0,675018	0,315604
POPKDL_0!	POPKDL_0!	virulence factor		0,928085	0,002374	1,021567	0,086525
POPKDL_0!	POPKDL_0!	hypothetica	N U	0,832316	0,094825	0,25468	0,810868
POPKDL_0!	POPKDL_0!	amidase		0,341338	0,25934	1,163746	0,061443
POPKDL_0!	POPKDL_0!	hypothetical protein		0,615643	0,039028	1,95104	0,00705

POPKDL_05	POPKDL_05	hypothetical protein		0,026135	0,955217	-0,30808	0,723267
POPKDL_05	POPKDL_05	hypothetica	S	-0,14722	0,676092	0,217188	0,754073
POPKDL_05	POPKDL_05	ISLre2 fami	J	0,33757	0,357543	1,756501	0,01191
POPKDL_05	rpmH	50S riboson	G	2,207469	0,090812	-2,69135	0,672526
POPKDL_05	nanE	Putative N-	G	-2,64821	7,34E-19	-2,65736	8,80E-07
POPKDL_05	POPKDL_05	PTS glucose	K	0,365402	0,229959	1,038344	0,078824
POPKDL_05	POPKDL_05	RpiR family	Q T	-2,73868	1,16E-19	-3,27239	0,000105
POPKDL_05	POPKDL_05	isochorism	E G P	-0,27408	0,463672	-0,62216	0,302652
POPKDL_05	POPKDL_05	Drug resista	L	2,474527	9,34E-10	3,237304	4,80E-08
POPKDL_05	tatD	hydrolase T	J	-5,19877	4,54E-53	-5,71249	1,95E-09
POPKDL_05	rnmV	ribonuclease M5		0,049674	0,834231	1,400383	0,38124
POPKDL_05	POPKDL_05	hypothetica	J	0,144424	0,921379	-2,84557	0,023421
POPKDL_05	rsmA	16S rRNA (a	T	0,887013	0,271148	2,343586	0,041676
POPKDL_05	POPKDL_05	Response r	T	-2,94117	3,41E-06	-0,94821	0,408527
POPKDL_05	POPKDL_05	ATP-binding	E	-1,12409	0,000142	-0,89025	0,150852
POPKDL_05	argF	ornithine ca	E	-2,11119	1,19E-13	-2,51517	9,05E-08
POPKDL_05	arcC	carbamate	S	-0,00664	1	0,086194	0,919246
POPKDL_05	POPKDL_05	C4-dicarbo	S	0,200777	0,567756	0,557134	0,317722
POPKDL_05	POPKDL_05	DUF4811 d	E G P	-0,41409	0,25488	0,563864	0,45133
POPKDL_05	POPKDL_05	MFS transp	G	1,104208	0,013086	0,517137	0,646884
POPKDL_05	rsgA	ribosome s	G	1,305718	0,101491	1,794229	0,139778
POPKDL_05	rpe	ribulose-ph	H	2,342527	0,0928	0,052618	1
POPKDL_05	thiN	thiamine di	S	0,728374	0,390204	3,09826	0,055606
POPKDL_05	rmuC	DNA recom	S	0,787992	0,012301	1,343274	0,011796
POPKDL_05	yhaM	3'-5' exonu	F	-2,77715	4,55E-12	-3,2254	1,85E-05
POPKDL_05	purR	pur operon	D	3,010459	1,38E-22	7,627408	4,80E-08
POPKDL_05	POPKDL_05	Surface exc	J	-0,11577	0,74423	0,844028	0,205079
POPKDL_05	rpsL	30S riboson	J	-3,16617	0,231529	0,172886	1
POPKDL_05	rpsG	30S riboson	J	1,237102	0,296081	-1,45627	0,675805
POPKDL_05	fusA	elongation	G	1,811748	0,018233	1,191624	0,442029
POPKDL_05	gap	type I glyce	S	-0,12681	1	-2,62642	0,257144

POPKDL_0!	POPKDL_0!	5'-nucleotic	S	-3,19427	5,25E-19	-3,97781	7,06E-15
POPKDL_0!	POPKDL_0!	UDP-N-ace	S	1,078712	0,01285	1,60063	0,136275
POPKDL_0!	obgE	GTPase Ob;	E	1,140682	0,33617	0,902588	0,578845
POPKDL_0!	POPKDL_0!	aminopepti	P	-0,34093	0,254209	-0,55216	0,393116
POPKDL_0!	POPKDL_0!	CorA-like3	P	0,795958	0,019507	0,599624	0,384721
POPKDL_0!	POPKDL_0!	Zinc transp	J	1,074507	0,000393	0,569566	0,459387
POPKDL_0!	POPKDL_0!	Pseudourid	S	-4,69825	8,59E-40	-5,04283	4,56E-16
POPKDL_0!	POPKDL_0!	diaminopin	E G P	1,059874	0,003106	0,561203	0,711349
POPKDL_0!	POPKDL_0!	MFS transp	S	-0,11143	0,754239	-0,011	1
POPKDL_0!	POPKDL_0!	UPF0371 pı	Q T	-1,87345	4,44E-10	-0,8465	0,207374
POPKDL_0!	paaI	3-hydroxya	F	0,04388	0,913791	0,201127	0,773311
POPKDL_0!	POPKDL_0!	Phosphoryl	F	2,421414	9,94E-10	2,434826	8,66E-05
POPKDL_0!	POPKDL_0!	hypothetica	P	-0,25268	0,615236	-0,38715	0,690239
POPKDL_0!	POPKDL_0!	NlpA lipopr	L	-3,23543	3,21E-30	-3,86264	4,21E-11
POPKDL_0!	POPKDL_0!	Transposas	L	0,637139	0,059044	0,165925	0,86435
POPKDL_0!	POPKDL_0!	helix-turn-h	L	0,472476	0,418398	-0,80824	0,674692
POPKDL_0!	POPKDL_0!	DUF262 do	J	-1,42767	9,23E-07	-1,1513	0,033153
POPKDL_0!	rlmD	23S rRNA (ı	K	0,769012	0,011519	0,437234	0,553403
POPKDL_0!	POPKDL_0!	LytR family	F	0,859318	0,003385	0,710898	0,207374
POPKDL_0!	POPKDL_0!	shikimate k	E	-3,84012	3,35E-24	-3,89025	1,24E-10
POPKDL_0!	aroA	3-phosphos	E	0,267471	0,419874	0,230093	0,752732
POPKDL_0!	brkB	ribonucleas	E	-1,82142	5,80E-07	-2,23407	0,001423
POPKDL_0!	map	methionyl a	K	5,584616	0,014081	5,852077	0,061443
POPKDL_0!	spxR	CBS-HotDc	J	-0,78186	0,02897	-0,91419	0,086525
POPKDL_0!	POPKDL_0!	RimJ/RimL	K	0,456806	0,166516	0,25152	0,710279
POPKDL_0!	POPKDL_0!	FadR family	G	0,975584	0,009623	0,540597	0,494264
POPKDL_0!	POPKDL_0!	Beta-glucuı	M	-1,1211	0,005731	-1,28254	0,18229
POPKDL_0!	murA2	UDP-N-ace	H	-0,54573	0,073788	0,154872	0,809408
POPKDL_0!	metK	methionine	M	-0,04443	1	1,878908	0,162416
POPKDL_0!	POPKDL_0!	internalin	H K	-1,95129	5,92E-11	-1,64707	0,000785
POPKDL_0!	birA	bifunctiona	L	1,322742	0,017467	0,660471	0,335462

POPKDL_05	dnaX	DNA polym	T	1,402634	0,081132	-0,79258	0,78672
POPKDL_05	POPKDL_05	GAF domai	S	6,218604	0,000406	3,885183	0,35371
POPKDL_05	POPKDL_05	DUF1294 d	F	1,099461	0,006532	0,507843	0,762188
POPKDL_05	udk	uridine kina	L	-2,81779	1,07E-19	-2,57834	6,58E-07
POPKDL_05	srmB	ATP-depend	G	-0,97741	0,626611	-1,98517	0,365212
POPKDL_05	POPKDL_05	deacetylase	C	-3,52905	5,27E-28	-3,38054	8,59E-11
POPKDL_05	POPKDL_05	NADP-depe	G	1,242888	0,034369	2,726027	0,091643
POPKDL_05	ptsI	Phosphoen	G	1,089714	0,317201	2,227476	0,049726
POPKDL_05	ptsH	phosphocarrier protein		2,22131	0,066324	-2,69135	0,672526
POPKDL_05	POPKDL_05	IS3 family t	S	-0,32481	0,314469	0,123761	0,862941
POPKDL_05	POPKDL_05	DUF960 do	J	-4,74286	5,89E-47	-4,58535	9,43E-17
POPKDL_05	ygaC	UPF0374 p	S	-1,69944	3,18E-06	-2,57074	3,38E-05
POPKDL_05	recX	recombinat	J	-0,19712	0,58382	0,260847	0,691033
POPKDL_05	rlmD	23S rRNA (u	G	-1,94468	1,92E-11	-1,59036	0,012232
POPKDL_05	POPKDL_05	alpha-mani	S	1,081963	0,000505	1,276003	0,021496
POPKDL_05	POPKDL_05	metal-indep	K	-2,06182	2,96E-14	-1,57819	0,011715
POPKDL_05	POPKDL_05	AraC family	G	-1,15543	3,33E-05	-1,01321	0,06771
POPKDL_05	POPKDL_05	Hyaluronida	G	-3,47535	1,13E-27	-3,23073	2,16E-09
POPKDL_05	bglB	6-phospho-	T	-1,82738	1,09E-10	-2,14991	5,46E-06
POPKDL_05	POPKDL_05	Histidine ki	G	-3,73723	2,73E-22	-4,48154	2,39E-14
POPKDL_05	POPKDL_05	hypothetica	G K	0,927876	0,020033	1,384509	0,015331
POPKDL_05	POPKDL_05	alpha-mannosidase		-3,08376	1,86E-26	-3,36954	9,08E-10
POPKDL_05	nagC	ROK family	P	0,355567	0,259808	0,673392	0,235934
POPKDL_05	lplB	sugar ABC t	P	0,266707	0,409042	0,331037	0,597059
POPKDL_05	ugpE	carbohydra	G	-1,11045	0,000351	-0,84568	0,093657
POPKDL_05	ugpB	sugar ABC t	S	-0,45161	0,135753	-0,57613	0,371712
POPKDL_05	POPKDL_05	DUF624 do	T	1,283688	5,46E-05	2,026109	0,000132
POPKDL_05	POPKDL_05	ATPase	T	-0,86805	0,001523	-0,96962	0,078915
POPKDL_05	POPKDL_05	Two-compo	G	-0,16735	0,606627	1,102699	0,220158
POPKDL_05	POPKDL_05	Beta-galact	E	-0,52378	0,066891	-0,71464	0,240907
POPKDL_05	aroE	shikimate c	E	-1,45354	1,07E-06	-1,42805	0,022689

POPKDL_05	aroB	3-dehydroq	E	-2,55735	5,94E-17	-0,04157	0,994906
POPKDL_05	aroF	bifunctional	3-deoxy-7-	0,303852	0,476671	0,329067	0,710017
POPKDL_05	POPKDL_05	hypothetical protein		-6,68148	3,75E-77	-6,9041	3,75E-15
POPKDL_05	POPKDL_05	hypothetical protein		0,842923	0,022605	0,50462	0,573979
POPKDL_05	POPKDL_05	IgG-degrad	K	-0,53433	0,07897	-0,305	0,65648
POPKDL_05	araC	YSIRK-targeted surface		-1,20074	0,006385	-2,00663	0,002596
POPKDL_05	POPKDL_05	alpha-like surface prote		-2,29772	1,07E-11	-2,93785	9,05E-08
POPKDL_05	POPKDL_05	hypothetica	J	-2,11194	2,63E-13	-1,97054	0,000201
POPKDL_05	tyrS	tyrosine--tR	M	0,988548	0,106365	1,407247	0,33832
POPKDL_05	pbp1b	penicillin-b	K	-0,05677	0,998339	-0,12174	0,951419
POPKDL_05	rpoB	DNA-direct	K	1,204324	0,025421	1,384599	0,225253
POPKDL_05	rpoC	DNA-direct	S	1,60028	0,006155	2,313854	0,100025
POPKDL_05	POPKDL_05	DUF1033 d	N U	-0,71704	0,147198	-1,60718	0,152002
POPKDL_05	POPKDL_05	competenc	N U	-0,7717	0,00891	-0,95928	0,195665
POPKDL_05	POPKDL_05	competenc	U	1,0624	0,000281	2,21636	0,002951
POPKDL_05	comGC	competenc	N U	-0,43663	0,362399	0,405437	0,671583
POPKDL_05	POPKDL_05	Competenc	N U	0,502941	0,101491	1,070158	0,034696
POPKDL_05	POPKDL_05	Type II secr	U	0,265251	0,567449	0,042524	0,99132
POPKDL_05	POPKDL_05	competenc	U	0,516951	0,089038	0,525589	0,381869
POPKDL_05	POPKDL_05	competenc	L	2,045651	0,013284	-4,11916	0,200876
POPKDL_05	ytxK	SAM-depen	F	0,902273	0,030942	0,038172	0,991483
POPKDL_05	ackA	Acetate kin	K	1,976745	0,01508	1,115596	0,485529
POPKDL_05	POPKDL_05	transcriptional regulato		1,095091	0,003301	2,038653	0,015477
POPKDL_05	POPKDL_05	Membrane	E	0,941485	0,046065	1,382106	0,092045
POPKDL_05	proC	pyrroline-5-	G	0,208877	0,661271	-0,32357	0,578991
POPKDL_05	pepA	glutamyl aminopeptida		1,480101	6,83E-06	1,345586	0,020569
POPKDL_05	POPKDL_05	hypothetica	S	-7,16528	5,01E-54	-7,70275	3,35E-12
POPKDL_05	POPKDL_05	DUF4651 d	C O	-0,04645	0,946803	-1,26886	0,515637
POPKDL_05	POPKDL_05	thioredoxin	J	-0,11684	0,776297	-0,38321	0,672526
POPKDL_05	POPKDL_05	DUF4479 d	L	0,993433	0,022614	1,690212	0,060241
POPKDL_05	ssb1	single-stran	F	0,686537	0,02216	0,774271	0,158775

POPKDL_0!	dck	deoxynucle	J	-0,88039	0,07824	-0,93823	0,315279
POPKDL_0!	dusB	tRNA dihydı	O	1,789738	1,61E-05	3,303707	0,000104
POPKDL_0!	hslO	Hsp33 fami	K	1,752893	0,009938	1,577319	0,043634
POPKDL_0!	POPKDL_0!	YSIRK-targe	M	-0,78789	0,005392	-0,55256	0,357273
POPKDL_0!	POPKDL_0!	Fibronectin	M	-0,23849	0,457803	0,030669	0,984973
POPKDL_0!	POPKDL_0!	SpaH/EbpB	M	0,424611	0,170148	-0,06593	0,964987
POPKDL_0!	POPKDL_0!	T-antigen-li	M	0,596537	0,115245	0,22466	0,843254
POPKDL_0!	POPKDL_0!	Class C sor	P	0,594901	0,080963	0,316856	0,607439
POPKDL_0!	POPKDL_0!	ABC transp	P	-3,59003	9,56E-28	-4,05176	2,07E-11
POPKDL_0!	POPKDL_0!	ABC transp	F	1,137285	0,00354	0,495227	0,602004
POPKDL_0!	srmB	ATP-depend	P	-0,32463	0,463672	0,864899	0,375852
POPKDL_0!	kup	putative po	L	0,312417	0,567449	1,317483	0,08248
POPKDL_0!	yhbQ	UPF0213 pı	L	1,138625	0,059056	1,263844	0,157818
POPKDL_0!	POPKDL_0!	tRNA1(Val)	I	0,649962	0,098664	0,845461	0,21827
POPKDL_0!	plsC	1-acyl-sn-g	L	0,717469	0,473532	0,096722	1
POPKDL_0!	POPKDL_0!	ComE oper	S	-4,1663	4,29E-40	-4,32338	1,14E-14
POPKDL_0!	POPKDL_0!	DNA interna	L	-0,17343	0,590982	-0,39042	0,563263
POPKDL_0!	holA	DNA polym	C O	-5,46614	2,20E-30	-5,88626	1,03E-12
POPKDL_0!	sodA	Superoxide	V	1,124489	0,302733	-0,91106	1
POPKDL_0!	POPKDL_0!	Beta-lactar	S	1,112546	0,000132	1,13651	0,036463
POPKDL_0!	POPKDL_0!	DUF3114 d	J	-1,02975	0,046642	-1,44262	0,012952
POPKDL_0!	queA	tRNA preQ1	G	-1,84069	2,65E-11	-0,32537	0,736914
POPKDL_0!	nagB	glucosamir	J	-0,0699	0,902654	0,825958	0,292562
POPKDL_0!	POPKDL_0!	Pseudourid	P	0,251458	0,606661	1,766646	0,021496
POPKDL_0!	POPKDL_0!	MFS transp	S	-0,22518	0,577078	-0,7239	0,222403
POPKDL_0!	coiA	competenc	E	-1,23709	0,000282	-1,4761	0,006958
POPKDL_0!	pepF	oligoendop	S	-1,31678	1,13E-05	-1,49126	0,00706
POPKDL_0!	POPKDL_0!	NADPH-deı	S	0,326574	0,365794	1,217834	0,065601
POPKDL_0!	POPKDL_0!	N-acetyltra	S	1,006768	0,001425	1,281251	0,0602
POPKDL_0!	POPKDL_0!	HAD family	P	0,420037	0,221667	-0,05547	0,984656
POPKDL_0(	POPKDL_0(	MFS transp	S	-0,06029	0,898512	-1,08866	0,084169

POPKDL_06	POPKDL_06	methyltrans	M	-4,09882	1,12E-32	-4,26683	5,15E-13
POPKDL_06	prsA	peptidylpro	J	-3,34902	2,50E-29	-4,11738	1,26E-08
POPKDL_06	alaS	alanine--tR	K	2,549051	1,77E-05	2,07644	0,078877
POPKDL_06	xRE	transcriptic	S	-0,99797	0,820115	3,261618	0,08042
POPKDL_06	POPKDL_06	DUF3169 d	S	-2,07394	2,15E-12	-2,25624	1,31E-06
POPKDL_06	POPKDL_06	CPBP famil	P	0,32844	0,302569	0,368873	0,53313
POPKDL_06	POPKDL_06	Voltage-gat	F	-2,14323	8,16E-08	-2,17744	0,000268
POPKDL_06	nrdF	class 1b rib	F	0,506604	0,533791	2,67349	0,08265
POPKDL_06	nrdE	class 1b rib	O	1,067489	0,177249	-1,11477	0,602004
POPKDL_06	nrdH	NrdH-redoxin		2,603962	0,015007	0,051489	1
POPKDL_06	POPKDL_06	hypothetica	S	-1,04264	0,267676	0,604234	0,741007
POPKDL_06	POPKDL_06	Hypothetica	L	-3,66999	3,03E-22	-4,22956	1,78E-09
POPKDL_06	POPKDL_06	DNA integra	L	-0,29128	0,435672	-0,50964	0,613553
POPKDL_06	mutL	DNA misma	E G P	-0,28129	0,453524	-0,53713	0,441294
POPKDL_06	POPKDL_06	MFS transp	L	-0,78081	0,117505	-1,62134	0,076105
POPKDL_06	ruvA	Holliday jur	L	1,424672	0,249666	1,044717	0,754073
POPKDL_06	POPKDL_06	3-methyladenine DNA g		2,186526	0,000505	0,473085	0,814929
POPKDL_06	POPKDL_06	hypothetica	E	-3,25072	0,377302	0,932996	0,78672
POPKDL_06	POPKDL_06	VOC family	S	-0,75821	0,011104	-1,06074	0,184353
POPKDL_06	POPKDL_06	competenc	L	0,890708	0,00347	0,963179	0,077162
POPKDL_06	recA	recombinas	K	1,064372	0,099641	0,383862	0,78672
POPKDL_06	spx	transcriptional regulatc		0,386625	0,657029	0,312033	0,822926
POPKDL_06	SSRC41		S	1,701217	0,081132	-3,57512	0,437463
POPKDL_06	POPKDL_06	IreB family	L	-3,51918	0,044831	-4,82253	0,052428
POPKDL_06	ruvX	Holliday jur	S	0,050106	0,884387	0,895983	0,693709
POPKDL_06	yrzB	UPF0473 p	S	5,401365	0,028471	6,441041	0,022826
POPKDL_06	POPKDL_06	DUF2079 d	F	0,664782	0,228285	1,33179	0,121626
POPKDL_06	nrdD	anaerobic ribonucleosi		-6,0322	3,86E-37	-4,61594	2,90E-06
POPKDL_06	POPKDL_06	30S ribosor	S	-0,25265	0,626644	-4,51329	0,097049
POPKDL_06	POPKDL_06	hypothetica	S	1,866232	0,049104	-0,91123	1
POPKDL_06	POPKDL_06	GNAT famil	O	0,354833	0,34687	-0,27807	0,810868

POPKDL_0(	nrdG	anaerobic r	S	-5,54304	6,41E-38	-4,43081	3,92E-05
POPKDL_0(	yaaA	peroxide st	K	0,04244	0,907226	0,291708	0,671583
POPKDL_0(	treR	trehalose o	G	0,466769	0,462195	-1,17874	0,291863
POPKDL_0(	POPKDL_0(	Phosphotra	G	-2,67142	3,01E-19	-3,32025	1,91E-06
POPKDL_0(	treC	alpha,alpha	O	1,364987	0,108198	1,529621	0,220074
POPKDL_0(	pepO	endopeptidase		-1,34621	1,97E-06	-2,00207	5,04E-05
POPKDL_0(	POPKDL_0(	Emm-like c	E	0,779306	0,032468	0,810529	0,193728
POPKDL_0(	POPKDL_0(	histidine triad protein		0,200892	0,525584	0,051655	0,959177
POPKDL_0(	POPKDL_0(	Collagen-like surface p		0,356479	0,406561	0,183682	0,85892
POPKDL_0(	POPKDL_0(	Transposas	S	0,06699	0,856291	-0,38631	0,632741
POPKDL_0(	mnmE	tRNA uridin	G	0,249201	0,685776	0,949995	0,478744
POPKDL_0(	rpiA	ribose-5-ph	G	-0,30383	1	1,420124	0,329139
POPKDL_0(	deoB	phosphope	F	-0,31125	0,43815	2,848453	0,009484
POPKDL_0(	xapA	purine-nucl	F	-0,76191	0,015503	0,898875	0,240907
POPKDL_0(	deoD	purine-nucl	T	-4,35212	1,40E-42	0,307665	0,85676
POPKDL_0(	POPKDL_0(	LytR family	K	-2,18959	4,41E-15	-2,38436	3,06E-06
POPKDL_0(	lysR	LysR family transcriptic		0,377461	0,516696	-0,7745	0,271693
POPKDL_0(	POPKDL_0(	hypothetica	F	0,720857	0,143726	0,692486	0,528763
POPKDL_0(	pyrF	orotidine-5'	F	-0,46162	0,349564	-3,49838	0,003098
POPKDL_0(	pyrE	orotate pho	K	-4,76866	9,73E-30	-8,28556	4,21E-25
POPKDL_0(	POPKDL_0(	GNAT famil	J	-2,321	5,12E-09	-2,5419	3,12E-06
POPKDL_0(	POPKDL_0(	amidase	E T	-2,15242	1,71E-13	-2,42301	5,72E-07
POPKDL_0(	hisJ	ABC transp	P	0,285438	0,370584	0,659577	0,192929
POPKDL_0(	hisM	amino acid	L	-0,33131	0,33773	0,635075	0,390136
POPKDL_0(	POPKDL_0(	Uracil-DNA	F	-1,35609	0,001232	-2,19745	3,63E-06
POPKDL_0(	pyrC	dihydroorot	I	-1,94897	0,000247	-1,89283	0,031156
POPKDL_0(	plsY	glycerol-3-p	L	-0,99757	0,820115	1,517657	0,504495
POPKDL_0(	parE	DNA topoisomerase IV		0,773402	0,247233	1,526555	0,267579
POPKDL_0(	POPKDL_0(	Phosphate	L	-1,15615	0,001208	-1,35791	0,047292
POPKDL_0(	parC	DNA topois	E	0,996213	0,166516	-0,95585	0,667057
POPKDL_0(	ilvE	branched-chain amino		-0,47844	0,172502	-0,33917	0,572112

POPKDL_0(	POPKDL_0(	DUF2969 domain-cont.		4,570597	0,055379		
POPKDL_0(	rpsA	30S ribosor	M	-1,40852	0,225501	0,512792	0,780936
POPKDL_0(	pbp3	D-alanyl-D-	G	-3,65173	1,03E-33	-3,39892	9,19E-10
POPKDL_0(	POPKDL_0(	deacetylas	E	-0,23866	0,4629	-0,22193	0,771421
POPKDL_0(	POPKDL_0(	Homoserin	L	-3,92752	1,11E-08	-5,75172	1,89E-06
POPKDL_0(	POPKDL_0(	NAD-binding-3 domain-		-0,90658	0,008966	-0,6424	0,306599
POPKDL_0(	POPKDL_0(	hypothetica	G	2,943866	4,29E-16	3,265031	3,84E-07
POPKDL_0(	glgP	maltose ph	G	0,241324	0,581122	0,765917	0,193724
POPKDL_0(	malQ	4-alpha-glu	K	-5,51529	4,13E-58	-7,03361	1,07E-17
POPKDL_0(	POPKDL_0(	LacI family	G	-0,01246	1	-0,39773	0,73298
POPKDL_0(	malE	sugar ABC t	P	0,562318	0,060076	0,838371	0,103109
POPKDL_0(	ugpA	sugar ABC t	P	1,447472	2,74E-07	1,466287	0,008199
POPKDL_0(	malG	sugar ABC t	K	1,220757	1,61E-05	1,182138	0,021114
POPKDL_0(	POPKDL_0(	HTH-type tr	M	-2,83127	7,10E-17	-2,85449	1,48E-05
POPKDL_0(	dltD	D-alanyl-lip	I Q	1,778303	0,00897	2,591127	0,014615
POPKDL_0(	dltC	D-alanine--	M	0,146299	0,946803	-4,82233	0,003055
POPKDL_0(	dltB	D-alanyl-lip	Q	-0,58826	0,465316	-2,36831	0,001304
POPKDL_0(	dltA	D-alanine--	S	-5,55241	2,56E-29	-6,30523	1,26E-20
POPKDL_0(	POPKDL_0(	teichoic aci	L	-4,73092	0,034138	-4,11916	0,200876
POPKDL_0(	uvrB	excinuclea:	P	-2,64919	5,85E-10	-2,4329	0,002031
POPKDL_0(	hisJ	amino acid	E	-0,89862	0,080963	-0,48564	0,697215
POPKDL_0(	glnQ	peptide AB(	S	-2,08163	0,001194	0,126743	1
POPKDL_0(	POPKDL_0(	hypothetica	G	-1,88713	4,24E-07	-2,07831	0,013147
POPKDL_0(	POPKDL_0(	PTS cellobi	S	-3,10979	1,57E-23	-2,70584	6,52E-08
POPKDL_0(	POPKDL_0(	DUF3284 d	G	1,583515	0,00486	2,620158	0,003879
POPKDL_0(	celC	PTS lactose	G	-0,83699	0,013748	-0,64896	0,383605
POPKDL_0(	celA	PTS sugar t	G	0,765297	0,063154	-0,03955	0,993684
POPKDL_0(	bglG	transcriptic	S	-2,11472	2,65E-14	-1,8054	0,000934
POPKDL_0(	POPKDL_0(	DUF871 do	G	-1,05009	0,003408	-1,42547	0,046523
POPKDL_0(	bglB	6-phospho-	H	1,369164	3,14E-05	1,334507	0,013173
POPKDL_0(	pnuC	nicotinamide riboside t		1,080191	0,000109	1,496092	0,013389

POPKDL_06	POPKDL_06	hypothetica	J	0,854982	0,003325	0,598405	0,3608
POPKDL_06	tsf	translation	J	3,107157	0,000254	2,580591	0,043504
POPKDL_06	rpsB	30S ribosor	T	1,369762	0,229799	2,215227	0,18739
POPKDL_06	POPKDL_06	LuxR family	E	1,108933	0,004506	2,810136	5,27E-05
POPKDL_06	hutG	formimidoy	E	0,923365	0,004081	0,569108	0,408144
POPKDL_06	hutH	histidine ar	E	-2,29899	1,36E-06	-1,08745	0,207374
POPKDL_06	potE	amino acid	S	0,596171	0,067052	0,177732	0,816554
POPKDL_06	POPKDL_06	HutD	F	-0,27982	0,622144	-0,46498	0,608719
POPKDL_06	fhs2	formate--te	E	-0,88351	0,016664	0,144748	0,860621
POPKDL_06	ftcD	sugar ABC t	E	-0,5604	0,202208	0,795728	0,434445
POPKDL_06	ftcD	glutamate f	E	-5,30065	1,03E-62	-4,41101	2,36E-13
POPKDL_06	hutU	urocanate h	Q	0,712623	0,012699	0,530742	0,377933
POPKDL_06	hutI	imidazolon	O	0,14239	0,721391	0,23612	0,754073
POPKDL_06	ahpF	alkyl hydrop	O	0,043059	0,91292	0,108465	0,8833
POPKDL_06	ahpC	alkyl hydrop	S	0,199799	0,566981	-0,35214	0,686548
POPKDL_06	POPKDL_06	carboxylest	S	0,84659	0,005429	1,08408	0,044518
POPKDL_06	POPKDL_06	Putative fla	E	-1,17039	0,000187	-0,90557	0,084768
POPKDL_06	POPKDL_06	ring-cleavir	E	2,127484	1,32E-09	1,768814	0,011841
POPKDL_06	POPKDL_06	ring-cleavir	S	-3,00311	6,66E-20	-3,10711	2,37E-07
POPKDL_06	POPKDL_06	folate famil	O	0,326247	0,34566	0,226763	0,73742
POPKDL_06	POPKDL_06	peptidase C5		-1,07473	0,000128	-1,24498	0,033798
POPKDL_06	POPKDL_06	Class C sortase		0,278189	0,406556	-0,01647	1
POPKDL_06	POPKDL_06	Ferrous iror	I	-1,11932	0,000741	-1,44893	0,02372
POPKDL_06	atoE	short-chain	K	0,325765	0,326883	-0,02282	0,999938
POPKDL_06	POPKDL_06	LysR family	I	-0,59134	0,041047	-0,6839	0,236655
POPKDL_06	POPKDL_06	acetyl-CoA	I	-1,28738	5,33E-06	-0,96209	0,08265
POPKDL_06	POPKDL_06	branched-c	I	0,620795	0,112359	0,660706	0,381869
POPKDL_06	atoA	Acyl-CoA:a	S	1,704253	7,12E-08	2,375356	5,54E-06
POPKDL_06	POPKDL_06	DNA-bindir	J	2,46003	3,05E-17	4,579658	1,83E-06
POPKDL_06	POPKDL_06	reactive int	G	-6,73376	1,29E-70	-6,37629	2,28E-25
POPKDL_06	frwC	transcriptional regulato		-2,14011	1,98E-13	0,225822	0,860098

POPKDL_0(	POPKDL_0(	hypothetica	C	0,677855	0,166889	0,999537	0,337469
POPKDL_0(	POPKDL_0(	V-type ATP	C	-1,45213	1,79E-07	-1,47587	0,008182
POPKDL_0(	POPKDL_0(	V-type ATP	C	2,10381	5,33E-10	2,136162	0,004138
POPKDL_0(	POPKDL_0(	ATPase V	C	1,326422	0,00324	1,122784	0,181468
POPKDL_0(	POPKDL_0(	V-type ATP	C	0,417113	0,163236	0,592285	0,334865
POPKDL_0(	POPKDL_0(	V-type ATP	C	1,752631	0,068369	0,25525	0,862941
POPKDL_0(	atpA	V-type ATP	C	0,543233	0,063154	0,384333	0,584632
POPKDL_0(	atpB	V-type ATP	C	0,624855	0,180139	0,546246	0,43715
POPKDL_0(	atpD	V-type ATP synthase su		1,584378	0,039455	2,646171	0,013437
POPKDL_0(	POPKDL_0(	hypothetical protein		3,200953	0,022978	4,440917	0,020038
POPKDL_0(	POPKDL_0(	hypothetica	F	0,100876	0,847278	-0,67337	0,634783
POPKDL_0(	purA	adenylosuc	S	-2,13948	2,14E-07	-2,16152	0,023956
POPKDL_0(	POPKDL_0(	BMP family	C	-4,23039	5,08E-40	-3,90992	1,84E-12
POPKDL_0(	pflA	glycyl-radic	K	1,179451	8,86E-05	0,623637	0,437463
POPKDL_0(	glpR	DeoR famil	K	0,412662	0,333828	0,597195	0,335649
POPKDL_0(	POPKDL_0(	DNA-bindir	L	-1,31337	1,23E-06	-1,41106	0,008935
POPKDL_0(	dnaI	primosoma	L	0,039301	1	0,943659	0,534594
POPKDL_0(	POPKDL_0(	helicase lo	T	0,287563	0,655408	1,27748	0,390272
POPKDL_0(	nrdR	transcriptional regulato		5,961524	0,003014	4,382734	0,178051
POPKDL_0(	kdpD	two-compo	T	-3,14874	2,70E-09	-3,54291	0,000701
POPKDL_0(	covR	two-compo	S	0,673642	0,377302	-2,90981	0,211545
POPKDL_0(	POPKDL_0(	Putative AC	O	-0,52719	0,50501	-0,9885	0,476743
POPKDL_0(	htpX	zinc metall	S	0,411131	0,272633	0,145418	0,887136
POPKDL_0(	lemA	LemA famil	J	0,24707	0,683547	0,390934	0,810868
POPKDL_0(	rsmG	16S rRNA (	P	0,181252	0,712091	2,11859	0,088715
POPKDL_0(	trkG	ATPase V	P	0,329888	0,546956	-0,89582	0,36761
POPKDL_0(	trkA	potassium	E	1,485522	0,00475	-0,61852	0,602004
POPKDL_0(	sstT	serine/thre	E	-2,21625	4,45E-05	-1,97526	0,08248
POPKDL_0(	brnQ	branched-c	P	-0,13936	0,736449	2,467173	0,006657
POPKDL_0(	metP	methionine	P	-1,00736	0,150128	-1,62101	0,129128
POPKDL_0(	metN	Methionine import ATP-		-3,3568	1,68E-14	-4,99896	6,66E-10

POPKDL_06	POPKDL_06	hypothetica	E	0,006948	0,941911	-0,65595	0,781946
POPKDL_06	POPKDL_06	M20-dimer	E	-3,22546	1,21E-12	-3,18026	9,16E-05
POPKDL_06	POPKDL_06	Acetylornitl	M	0,123868	0,794098	-0,32038	0,751739
POPKDL_06	nlpA	O-sialoglyc	E T	-1,90485	0,013223	-1,83334	0,054075
POPKDL_06	hisJ	amino acid	K	-0,22877	0,612939	-1,09962	0,249199
POPKDL_06	tACO1	YebC/PmpI	K	3,303416	4,83E-06	2,860149	0,060184
POPKDL_06	POPKDL_06	HTH-type tr	S	0,635499	0,032111	0,236521	0,756621
POPKDL_06	POPKDL_06	nucleotidyl	Q	-1,66879	2,12E-08	-1,74014	0,000539
POPKDL_06	ubiE	SAM-depen	J	0,449047	0,249875	-0,25255	0,843254
POPKDL_06	rsfS	ribosome s	Q	-2,25494	0,002719	-2,72838	0,009283
POPKDL_06	POPKDL_06	Streptothric	H	-0,11989	0,902654	-1,48762	0,214864
POPKDL_06	yqeK	bis(5'-nucle	H	0,7065	0,458059	1,802152	0,1784
POPKDL_06	nadD	nicotinate-i	J	0,205384	0,821146	-1,85026	0,437463
POPKDL_06	yhbY	ribosome a	S	-0,6901	0,365794	-1,85047	0,437463
POPKDL_06	yqeH	ribosome b	S	2,25707	0,011956	2,852898	0,01131
POPKDL_06	yqeG	YqeG family HAD IIIA-ty		2,308245	0,042962	3,106066	0,092045
POPKDL_06	POPKDL_06	helix-turn-helix domain		1,005077	0,003022	1,619147	0,00706
POPKDL_06	POPKDL_06	Transposase-like protei		2,12223	0,622144		
POPKDL_06	POPKDL_06	IS30 family transposas		2,892622	0,001112	-0,01832	1
POPKDL_06	POPKDL_06	Competence protein C		-1,75032	6,40E-08	-2,24625	0,001396
POPKDL_06	POPKDL_06	TIGR00266	P	0,251264	0,617424	1,774617	0,09115
POPKDL_06	POPKDL_06	carbonate c	O	1,173654	0,199399	1,27003	0,520141
POPKDL_06	radA	DNA repair	F	-2,54056	7,43E-19	-3,03049	8,82E-09
POPKDL_06	dut	dUTP diphosphatase		0,720034	0,600044	1,329578	0,315604
POPKDL_06	POPKDL_06	Abi-like pro	C	1,222458	3,55E-05	1,624841	0,001902
POPKDL_06	queH	Epoxyqueu	V	0,688775	0,034186	0,937083	0,091643
POPKDL_06	mdlB	multidrug A	V	0,763818	0,011936	2,226394	0,000622
POPKDL_06	POPKDL_06	multidrug A	K	-2,99966	2,41E-23	-3,38204	5,80E-10
POPKDL_06	POPKDL_06	MarR family	I	1,353795	0,000643	0,771862	0,352949
POPKDL_06	gpsA	NAD(P)H-d	M	1,068761	0,103771	0,188316	0,956438
POPKDL_06	hasC	UTP--gluco	O	1,864536	0,084386	3,594808	0,015254

POPKDL_06	POPKDL_06	rhomboid fa	H	2,161311	1,03E-08	2,632191	2,80E-06
POPKDL_07	POPKDL_07	5-formyltet	K	1,479158	1,23E-05	2,277158	0,002212
POPKDL_07	POPKDL_07	Transcriptio	G	0,023736	0,955217	0,172405	0,806426
POPKDL_07	pgi	glucose-6-p	S	-0,48821	0,870019	-0,99162	0,483143
POPKDL_07	POPKDL_07	hypothetical protein		0,75303	0,248008	0,702212	0,466476
POPKDL_07	Bacteria_small_SRP		D	-3,25072	0,377302	-2,69135	0,672526
POPKDL_07	POPKDL_07	S-layer prot	F J	0,774486	0,040292	0,648019	0,292947
POPKDL_07	tadA	tRNA adeno	S	1,529886	0,067004	1,257532	0,398369
POPKDL_07	elaC	MBL fold m	S	-5,36502	9,78E-25	-5,10179	2,82E-13
POPKDL_07	bioY	biotin trans	S	0,243679	0,42636	-0,14323	0,866443
POPKDL_07	POPKDL_07	CHY-type d	F	1,159964	0,00155	1,957826	0,00075
POPKDL_07	tgt	tRNA guano	S	0,723976	0,056829	0,449716	0,605818
POPKDL_07	POPKDL_07	DUF975 do	L	-1,21241	0,00015	-0,91867	0,144131
POPKDL_07	POPKDL_07	DNA-dama	D	0,278966	0,533778	-0,43608	0,804526
POPKDL_07	parE	type II toxin	H	0,988975	0,005129	0,82573	0,312037
POPKDL_07	folC	dihydrofola	F	0,590025	0,573912	0,059128	1
POPKDL_07	folE	GTP cycloh	H	-0,99361	0,673786	1,969989	0,222345
POPKDL_07	folP	dihydropter	H	1,520349	0,26214	2,940509	0,083779
POPKDL_07	folB	dihydroneo	H	1,181448	0,303009	0,051601	1
POPKDL_07	folK	2-amino-4-	M	2,122031	0,622144	2,771819	0,675805
POPKDL_07	murB	UDP-N-ace	P	-0,47511	0,721391	-1,45649	0,675805
POPKDL_07	potA	Spermidine	P	-3,2811	1,39E-10	-3,13806	1,99E-07
POPKDL_07	potB	spermidine	P	-1,40016	7,20E-05	0,454892	0,672526
POPKDL_07	potC	spermidine	P	-4,19078	2,19E-34	-3,5539	5,10E-06
POPKDL_07	potD	spermidine	P	-2,49566	3,94E-12	-1,45557	0,145142
POPKDL_07	POPKDL_07	Ribonuclea	K	0,054465	0,883945	-0,23632	0,74691
POPKDL_07	POPKDL_07	Regulatory	E	-2,62955	2,47E-20	-2,64311	3,67E-07
POPKDL_07	POPKDL_07	lipase	S	-1,05016	0,001579	-0,24382	0,754745
POPKDL_07	POPKDL_07	AI-2E family	E	-1,42231	1,56E-06	-0,87734	0,139778
POPKDL_07	radC	DNA repair	T	2,410915	1,52E-14	2,99396	1,36E-07
POPKDL_07	POPKDL_07	gamma-glu	K	-0,79161	0,112253	-2,83955	0,020291

POPKDL_07	rex	redox-sens	S	-1,24534	0,799056	2,481218	0,097049
POPKDL_07	POPKDL_07	DUF4649 d	S	-8,38417	8,24E-81	-7,30259	8,05E-21
POPKDL_07	POPKDL_07	cysteine de	E	1,263619	0,016092	0,354051	0,774358
POPKDL_07	POPKDL_07	cysteine de	F	0,374505	0,922085	0,771892	0,664711
POPKDL_07	prs2	ribose-phos	S	2,312627	0,00264	0,530438	0,920386
POPKDL_07	POPKDL_07	adenylate c	S	-3,51446	1,80E-32	-4,48456	1,41E-12
POPKDL_07	yjbM	GTP pyroph	H	0,67315	0,081471	1,017919	0,165187
POPKDL_07	nadK	NAD kinase	J	1,720003	0,067201	0,568558	0,809606
POPKDL_07	rluA	RluA family	C	0,501174	0,186005	1,1197	0,071693
POPKDL_07	pta	phosphate	S	0,280252	0,870019	-6,05551	0,012817
POPKDL_07	POPKDL_07	NAD(P)-dep	S	-1,23143	6,24E-06	-1,46945	0,007382
POPKDL_07	POPKDL_07	multidrug transporter		-3,88586	4,05E-20	-2,6884	0,001189
POPKDL_07	trhO	rhodanese-	O	-2,79538	2,56E-17	-3,80924	1,11E-09
POPKDL_07	POPKDL_07	glutathione	P	-3,68079	3,42E-30	-4,55374	2,37E-05
POPKDL_07	POPKDL_07	C4-dicarbo	S	0,453654	0,190969	0,58044	0,43822
POPKDL_07	POPKDL_07	DUF3042 d	J	0,341825	0,386621	-0,15634	0,940809
POPKDL_07	miaA	tRNA (aden	S	0,542876	0,268136	-0,76161	0,640468
POPKDL_07	hflX	GTPase HflX	G	1,211629	1,25E-05	2,616735	0,000127
POPKDL_07	POPKDL_07	cystathioni	S	1,022997	0,002783	1,156915	0,0602
POPKDL_07	rnz	ribonucleas	S	0,930523	0,326777	-0,90036	0,779191
POPKDL_07	yqjQ	short-chain	L	0,657409	0,030468	2,936893	0,000274
POPKDL_07	recJ	single-stran	F	-0,09749	0,765122	1,269297	0,105653
POPKDL_07	apt	adenine ph	L	-0,49102	0,377302	0,579896	0,674692
POPKDL_07	POPKDL_07	DNA replica	L	1,006841	0,2357	0,827993	0,799968
POPKDL_07	nth	endonuclea	S	-5,33081	4,44E-61	-4,06378	2,97E-07
POPKDL_07	POPKDL_07	tRNA (aden	S	0,237366	1	-1,45678	0,675805
POPKDL_07	POPKDL_07	Nif3-like di	P	-2,82222	1,68E-14	-2,628	0,000268
POPKDL_07	POPKDL_07	ZIP family n	E	-0,4631	0,219542	-0,83188	0,227867
POPKDL_07	POPKDL_07	FAD-depen	M	0,807032	0,024963	2,465009	0,003598
POPKDL_07	rfbA	glucose-1-p	M	6,081559	0,000382	6,787043	0,00234
POPKDL_07	rfbC	Protein Rml	M	1,009757	0,266916	-0,90052	0,779191

POPKDL_0	rfbB	dTDP-glucose 4,6-dehy	0,862358	0,324091	-4,11916	0,200876
POPKDL_0	POPKDL_0	Isoleucyl-tRNA syntheta	0,394096	0,258337	0,950119	0,108781
POPKDL_0	POPKDL_0	6-phosphogluconolacto	1,133028	0,001236	1,452724	0,025811
POPKDL_0	yjhB	DNA mismatch repair p	0,277198	0,787655	-1,34055	0,383531
POPKDL_0	perM	AI-2E family transporte	-0,81885	0,007273	-0,53871	0,375012
POPKDL_0	lapB	PARP alpha-helical dom	1,850973	4,98E-07	1,853049	0,000118
POPKDL_0	fbp54	Rqc2 family fibronectin	0,211813	0,566981	-0,28133	0,658785
POPKDL_0	POPKDL_0	N-acetylmannosamine	-2,157	1,03E-12	-1,93193	0,000942
POPKDL_0	POPKDL_0	RpiR family transcriptio	-0,67676	0,020649	0,441288	0,627018
POPKDL_0	POPKDL_0	conjugal transfer protei	-1,20789	1,12E-05	-0,96169	0,08675
POPKDL_0	POPKDL_0	hypothetical protein	2,565477	3,92E-09	2,839224	0,000545
POPKDL_0	POPKDL_0	hypothetical protein	-8,16763	4,15E-42	-7,7133	1,63E-16
POPKDL_0	POPKDL_0	Methyltransferase	-2,39305	1,11E-17	-2,57493	1,29E-07
POPKDL_0	POPKDL_0	Single-stranded DNA-b	0,800276	0,006577	1,239568	0,014132
POPKDL_0	POPKDL_0	Agglutinin receptor	0,880443	0,003771	1,007676	0,08116
POPKDL_0	POPKDL_0	cell wall anchor protein	-4,49436	1,46E-07	-7,41998	8,42E-23
POPKDL_0	POPKDL_0	peptidase C39	-7,55761	1,38E-80	-6,31772	4,11E-14
POPKDL_0	POPKDL_0	hypothetical protein	1,678503	0,028154	2,276211	0,136355
POPKDL_0	POPKDL_0	conjugal transfer protei	0,482885	0,704831	-0,89951	0,779191
POPKDL_0	POPKDL_0	ATP-depend L	-4,95716	3,45E-19	-4,57215	9,35E-07
POPKDL_0	POPKDL_0	DNA topois L	-7,32606	3,96E-53	-7,83629	2,47E-29
POPKDL_0	POPKDL_0	DUF6782 d M	-2,68885	1,68E-14	-3,16654	4,86E-07
POPKDL_0	cas8c	type I-C CR L	-1,82136	3,18E-07	-2,17719	0,000155
POPKDL_0	cas7c	type I-C CR L	1,28401	0,127713	1,586178	0,312037
POPKDL_0	cas4	CRISPR-ass L	0,061776	0,883322	0,392878	0,600698
POPKDL_0	cas1c	type I-C CR L	0,282595	0,454894	1,164251	0,11125
POPKDL_0	cas2	CRISPR-ass S	0,067592	0,885464	-0,55625	0,462021
POPKDL_0	POPKDL_0	gfo/Idh/Mo O	-2,19819	1,03E-10	-2,26313	0,000186
POPKDL_0	ccdA2	thiol-disulfi C O	-0,26248	0,498283	-0,46463	0,429055
POPKDL_0	bcp	Peroxiredo O	-1,46129	5,82E-07	-1,90266	0,000942
POPKDL_0	msrAB	Peptide me T	-3,49678	3,08E-33	-3,92606	1,94E-13

POPKDL_07	yesN	DNA-bindir	T		-0,84029	0,025301	-0,04271	0,973514
POPKDL_07	yesM	sensor histi	S		-0,41851	0,201386	-0,33971	0,571619
POPKDL_07	POPKDL_07	MmcQ fami	K		-0,33381	0,319671	-0,05924	0,951419
POPKDL_07	POPKDL_07	B3-4 domai	K		0,846701	0,015687	0,284241	0,756621
POPKDL_07	argR	Arginine rep	K		-0,1903	1	0,272748	0,899648
POPKDL_07	crp	Crp/Fnr fan	E		1,322194	1,04E-06	1,354533	0,013243
POPKDL_07	arcA	arginine de	K		-6,94379	1,86E-43	-8,12129	1,70E-29
POPKDL_07	POPKDL_07	Histone ace	E		-1,17566	0,122998	-1,55153	0,184353
POPKDL_07	argF	ornithine ca	S		-1,04419	0,002277	-1,29076	0,043907
POPKDL_07	yfcC	arginine:orn	E		0,867169	0,005769	0,444113	0,523536
POPKDL_07	argE	Peptidase family M20/M			-1,8099	3,32E-09	-0,96997	0,141282
POPKDL_07	POPKDL_07	hypothetica	F		-1,09787	0,000289	-1,61083	0,086525
POPKDL_07	ndk	nucleoside	M		0,728612	0,633676	-4,51329	0,097049
POPKDL_07	lepA	translation	O		-3,83824	6,77E-29	-4,05713	2,49E-12
POPKDL_07	msrB	Peptide me	S		1,382392	2,29E-05	1,433263	0,158775
POPKDL_07	yeiH	UPF0324 m	G		0,176868	0,634209	0,063765	0,945047
POPKDL_07	manX	PTS manno	G		0,879943	0,015687	0,490276	0,527025
POPKDL_07	agaB	PTS manno	G		1,112051	0,007302	2,133454	0,021567
POPKDL_07	manY	PTS manno	G		-2,04649	5,03E-12	-1,92259	0,001997
POPKDL_07	manZ	PTS manno	T		1,922988	3,70E-09	1,937497	2,39E-05
POPKDL_07	POPKDL_07	sensor histi	K		-1,06737	0,001015	-0,7609	0,162856
POPKDL_07	POPKDL_07	DNA-bindir	P		0,130091	0,743712	0,125831	0,887136
POPKDL_07	afuA	iron ABC tra	S		1,036227	0,000812	1,428843	0,005652
POPKDL_07	POPKDL_07	zinc/iron-ch	G K		-1,27556	0,003732	-0,53896	0,453583
POPKDL_07	POPKDL_07	maltose ac	E		-0,19334	0,578723	-0,462	0,613847
POPKDL_07	POPKDL_07	YjjG family	C		0,034494	0,959482	-0,37408	0,675805
POPKDL_07	adhE	succinate-s	L		0,544998	0,109942	0,610551	0,317722
POPKDL_07	uvrC	excinucleas	E H		-0,93111	0,085597	-0,55435	0,46609
POPKDL_07	spxB	pyruvate ox	C		-0,17976	0,58415	-0,12601	0,862941
POPKDL_07	nfnB	Putative NA	E		1,54177	0,000589	1,823539	0,000314
POPKDL_07	pepV	dipeptidase	L		1,034093	0,015007	0,556701	0,609834

POPKDL_07	POPKDL_07	Transposas	L	-3,25072	0,377302	4,282469	0,023488
POPKDL_07	POPKDL_07	Integrase core domain		1,105203	0,377902	-1,38835	0,862941
POPKDL_07	POPKDL_07	copper ABC	S	-1,4622	1,36E-07	-1,0848	0,048847
POPKDL_07	uup	ABC-F fami	J	-2,42646	9,61E-07	-2,39397	0,000529
POPKDL_07	yitT	YitT family p	F	0,028531	0,988947	0,165716	0,983117
POPKDL_07	trpS	tryptophan-	U	5,328271	0,00855	7,334342	0,000139
POPKDL_07	guaB	IMP dehydr	L	0,218381	0,693713	1,109061	0,36298
POPKDL_07	glcU	Putative su	S	1,280265	0,057923	3,256263	0,003906
POPKDL_07	recF	DNA replica	S	-1,76255	0,000102	-2,38684	0,063783
POPKDL_07	yaaA	S4 domain-	S	-0,34293	0,565673	-0,30873	0,78672
POPKDL_07	POPKDL_07	insulinase f	S	-2,04845	2,60E-12	-2,19402	9,33E-06
POPKDL_07	POPKDL_07	insulinase family protei		0,751456	0,094627	0,129137	0,905455
POPKDL_07	POPKDL_07	Putative me	I	0,005185	1	-0,87507	0,3489
POPKDL_07	pgsA	CDP-diacyl	P	-0,32222	0,720781	3,247207	0,030432
POPKDL_07	POPKDL_07	energy-cou	P	0,039997	1	0,998114	0,497386
POPKDL_07	POPKDL_07	energy-cou	P	0,8247	0,461431	0,200348	0,944503
POPKDL_07	ecfT	cobalt ABC	M	-0,76992	0,267607	0,567547	0,764582
POPKDL_07	POPKDL_07	transglycos	F	1,486663	0,001886	1,658122	0,04738
POPKDL_07	adk	DNA topolo	D	-3,01189	2,58E-15	-2,28363	4,24E-07
POPKDL_07	POPKDL_07	Immunoglo	K	0,473291	0,1759	8,427491	3,55E-05
POPKDL_07	POPKDL_07	AraC family	G	0,365461	0,431452	0,023345	1
POPKDL_07	POPKDL_07	ABC transp	P	0,11825	0,745146	0,810844	0,162633
POPKDL_07	POPKDL_07	sugar ABC t	G	1,028496	0,000308	0,891205	0,11068
POPKDL_07	POPKDL_07	sugar ABC t	G	1,008462	0,001119	1,009761	0,040631
POPKDL_07	POPKDL_07	alpha-galac	G	-1,98341	1,77E-12	-1,98879	9,59E-05
POPKDL_07	POPKDL_07	beta-galactosidase		-1,16545	0,003041	-1,13027	0,060911
POPKDL_07	POPKDL_07	hypothetica	G	1,386533	0,013135	0,954596	0,337469
POPKDL_07	POPKDL_07	galactose n	K	-3,07787	5,80E-21	-3,26856	1,08E-06
POPKDL_07	POPKDL_07	LacI family	G	-1,82616	6,04E-09	-2,18755	0,000128
POPKDL_07	galK	galactokina	G	-2,20912	9,19E-16	-2,31167	2,03E-05
POPKDL_07	galT	UDP-glucos	M	0,785619	0,007297	0,743079	0,281947

POPKDL_0	galE	UDP-glucos	L	1,339774	6,28E-05	1,142966	0,07114
POPKDL_0	POPKDL_0	Integrase	P	-2,72732	7,31E-12	-2,78394	6,01E-05
POPKDL_0	oppF	Oligopeptic	P	1,813746	0,000109	1,131066	0,236655
POPKDL_0	dppD	ABC transp	E P	-7,37732	2,24E-72	-7,39622	6,85E-20
POPKDL_0	dppC	ABC transp	P	-4,09472	1,61E-32	-4,20429	8,99E-13
POPKDL_0	dppB	peptide AB	E	-3,03391	8,08E-25	-2,95189	1,05E-06
POPKDL_0	POPKDL_0	peptide AB	M	-6,9313	1,51E-71	-7,92055	3,15E-21
POPKDL_0	pbp3	D-alanyl-D-	M	-2,25139	2,44E-16	-2,46606	1,54E-05
POPKDL_0	POPKDL_0	D-alanyl-D-	O	-0,75424	0,015007	-0,2591	0,693709
POPKDL_0	sufB	Fe-S cluste	C	0,786435	0,258474	1,982367	0,146809
POPKDL_0	iscU	SUF system	E	-8,29105	1,38E-51	-5,36775	2,22E-09
POPKDL_0	sufS	cysteine de	O	-2,7456	5,13E-10	-3,98795	1,27E-05
POPKDL_0	sufD	Fe-S cluste	O	-4,83464	3,29E-32	-5,99464	1,94E-11
POPKDL_0	sufC	Fe-S cluste	M	-7,2876	5,34E-60	-6,88929	9,74E-23
POPKDL_0	rfe	undecapre	N O T	2,076711	0,001354	1,982604	0,130745
POPKDL_0	mecA	adaptor prc	V	0,681357	0,459009	0,665004	0,799583
POPKDL_0	uppP	undecaprenyl-diphospl		0,469852	0,423849	3,408363	0,017359
POPKDL_0	POPKDL_0	hypothetica	M	0,879621	0,024074	0,847385	0,602004
POPKDL_0	POPKDL_0	Thiol-activa	H	-0,05982	0,87374	-0,03752	0,981295
POPKDL_0	lplA	lipoate--prc	S	-0,96035	0,022733	-1,03364	0,183941
POPKDL_0	POPKDL_0	glutamine a	M	-1,46509	0,60045	0,143263	1
POPKDL_0	POPKDL_0	Lipid II isog	S	-0,39495	1	2,356599	0,097049
POPKDL_0	cdaA	diadenylate	S	1,240233	0,097959	0,956018	0,485529
POPKDL_0	POPKDL_0	YbbR-like p	G	-0,58419	0,163762	-0,52386	0,501125
POPKDL_0	glmM	phosphoglu	J	1,272653	0,148778	-1,61949	0,353629
POPKDL_0	POPKDL_0	Acetyltrans	H	-0,55313	0,056203	-0,74823	0,256159
POPKDL_0	hemW	radical SAM	I	-0,03161	0,958726	0,556776	0,343913
POPKDL_0	POPKDL_0	acyl-[acyl-c	G	0,258636	0,661499	0,228437	0,823482
POPKDL_0	nagD	TIGR01457	S	-0,15743	0,685605	-0,69358	0,370835
POPKDL_0	POPKDL_0	TIGR01906 family mem		0,519915	0,070929	0,187275	0,79381
POPKDL_0	tracrRNA		L	-1,20044	1	-2,69135	0,672526

POPKDL_07	POPKDL_07	CRISPR-ass	L	-2,70424	1,19E-18	-0,93048	0,329139
POPKDL_07	cas1	type II CRIS	L	-0,66665	0,034811	-0,05342	0,951419
POPKDL_07	cas2	CRISPR-ass	S	1,238142	0,000682	1,654057	0,017399
POPKDL_07	csn2	type II-A CR	S	-2,33676	8,05E-14	-2,29257	2,16E-05
POPKDL_07	thiT	energy-cou	M	-1,31767	0,000931	-1,93736	0,04119
POPKDL_07	POPKDL_07	N-acetylmu	P	1,15519	0,074253	0,984372	0,374401
POPKDL_07	POPKDL_07	ECF transp	P	-1,35111	0,014493	-0,41644	0,818183
POPKDL_07	POPKDL_07	cobalamin	O	1,162733	0,002348	0,866158	0,317722
POPKDL_07	btuE	Thioredoxir	E	0,798251	0,005166	0,800721	0,141851
POPKDL_07	pepF	oligoendop	H	2,435229	8,23E-08	2,93425	6,52E-08
POPKDL_07	ppc	phosphoen	D	-1,20666	0,001838	-0,10231	0,962457
POPKDL_07	ftsW	cell divisior	K	0,543331	0,403244	0,935248	0,658561
POPKDL_07	POPKDL_07	DNA-direct	S	0,804556	0,00834	0,545829	0,390784
POPKDL_08	POPKDL_08	Sigma facto	J	-1,38095	0,000218	-2,0924	0,008882
POPKDL_08	tuf	elongation	P	2,211546	0,005397	2,721536	0,035343
POPKDL_08	POPKDL_08	VTC domair	S	-0,49026	0,121088	-0,00284	1
POPKDL_08	POPKDL_08	DUF4956 d	M	-0,6751	0,023411	-0,61256	0,272209
POPKDL_08	POPKDL_08	CotH kinas	M	-0,42182	0,188672	-0,25204	0,672526
POPKDL_08	POPKDL_08	hypothetical protein		1,688065	1,09E-07	2,130557	0,000104
POPKDL_08	POPKDL_08	amino acid	E	-1,62244	0,033088	-3,0382	1,19E-07
POPKDL_08	glnQ	amino acid	P	-2,80219	3,99E-05	-2,63067	0,000498
POPKDL_08	hisM	amino acid	P	-4,82678	2,17E-11	-4,28607	1,99E-07
POPKDL_08	phnA	protein Phn	L	0,527097	0,189614	0,417514	0,709066
POPKDL_08	tnpA	IS200/IS60	M	1,497195	0,01139	0,365096	0,810868
POPKDL_08	glmS	glutamine--	U	-0,15199	1	1,030179	0,386954
POPKDL_08	lepB	signal pepti	G	-1,2163	0,005117	-0,62575	0,376269
POPKDL_08	pyk	pyruvate kir	F	0,184064	0,673167	1,575146	0,2983
POPKDL_08	pfkA	6-phosphof	L	2,450596	0,005565	0,471976	0,903333
POPKDL_08	dnaE	DNA polym	K	1,231329	0,06288	1,580737	0,130407
POPKDL_08	yhcF	GntR family	V	-0,25365	1	-2,69135	0,672526
POPKDL_08	POPKDL_08	ABC transp	V	0,464517	0,622144	-0,85562	0,315604

POPKDL_0{	POPKDL_0{	ABC transp	S	0,971687	0,117031	0,580689	0,478786
POPKDL_0{	POPKDL_0{	SNARE ass	S	0,58726	0,193704	0,439331	0,66726
POPKDL_0{	ssrA			-0,01152	1		
POPKDL_0{	POPKDL_0{	DUF3862 domain-cont		-3,25072	0,377302	-1,18355	0,412437
POPKDL_0{	abiEi	abortive phage infectio		-0,28747	0,787655	3,090569	0,107623
POPKDL_0{	POPKDL_0{	abortive ph	M	-1,4154	2,95E-07	-1,08946	0,048261
POPKDL_0{	POPKDL_0{	CHAP doma	U	0,872377	0,01092	1,672976	0,002484
POPKDL_0{	virB4	AAA family	S	-2,97426	1,64E-27	-3,09636	2,06E-06
POPKDL_0{	POPKDL_0{	Tn5252 Orf	S	0,731749	0,033304	0,849086	0,210932
POPKDL_0{	POPKDL_0{	conjugal tr	S	-0,06948	0,849254	-0,14873	0,85892
POPKDL_0{	POPKDL_0{	hypothetica	U	-6,61646	3,30E-43	-5,4029	1,13E-14
POPKDL_0{	virD4	conjugal tr	S	-0,89969	0,001952	-0,98229	0,09513
POPKDL_0{	POPKDL_0{	hypothetica	P	1,133056	8,03E-05	1,114754	0,018992
POPKDL_0{	POPKDL_0{	CAAX amin	S	-0,32119	0,374046	-0,58674	0,282776
POPKDL_0{	POPKDL_0{	transcriptic	P	0,524844	0,24814	0,023968	1
POPKDL_0{	arsC	Regulatory protein Spx		0,530293	0,200363	0,96107	0,211747
POPKDL_0{	POPKDL_0{	hypothetical protein		-1,62074	4,45E-05	-2,14301	0,004903
POPKDL_0{	POPKDL_0{	hypothetica	H	-0,3829	0,218201	-0,72425	0,250277
POPKDL_0{	POPKDL_0{	DNA (cytosine-5-)-metl		2,357753	3,63E-07	3,2944	1,79E-09
POPKDL_0{	POPKDL_0{	hypothetica	S	0,143038	0,711276	-0,13563	0,876004
POPKDL_0{	POPKDL_0{	replication initiator pro		-0,89906	0,002727	-1,13317	0,057068
POPKDL_0{	POPKDL_0{	hypothetical protein		-9,87348	1,92E-22	-6,7157	1,95E-07
POPKDL_0{	rli38		J	0,801961	0,058501	0,660815	0,501766
POPKDL_0{	rplL	50S ribosor	J	0,742663	0,553521	-4,51329	0,097102
POPKDL_0{	rplJ	50S ribosor	L	-1,57186	0,641713	-4,51329	0,097049
POPKDL_0{	POPKDL_0{	HTH-38 dor	L	0,392212	0,423849	-0,32611	0,822744
POPKDL_0{	POPKDL_0{	IS30 family transposas		-1,37052	5,02E-06	-0,66419	0,287654
POPKDL_0{	POPKDL_0{	Integrase core domain		0,890264	0,06866	0,225578	0,898317
POPKDL_0{	POPKDL_0{	VOC family	M	-1,35183	0,001564	-1,41662	0,160911
POPKDL_0{	POPKDL_0{	class A sort	L	0,405369	0,756973	1,38224	0,364867
POPKDL_0{	gyrA	DNA gyrase	C	2,010917	0,032638	2,372053	0,060037

POPKDL_08	ldh	L-lactate de	P	1,261457	0,093909	0,160261	1
POPKDL_08	fadH2	putative NA	V	0,411932	0,195817	0,195416	0,788546
POPKDL_08	POPKDL_08	hypothetica	M	3,1105	0,001077	1,847995	0,271693
POPKDL_08	pvaA	Pneumococ	S	0,198787	0,609	0,030604	0,996857
POPKDL_08	POPKDL_08	nucleoid-as	E	-6,06478	2,85E-58	-5,98562	3,15E-21
POPKDL_08	glyA	Serine hydr	K	-0,17905	0,58415	-0,58466	0,476743
POPKDL_08	POPKDL_08	GNAT famil	J	-1,63929	9,23E-08	-1,87157	0,001569
POPKDL_08	tsaC	L-threonylcarbamoylad		6,617014	0,000229		
POPKDL_08	prmC	peptide cha	J	-1,98631	1,16E-05	-3,81048	5,12E-05
POPKDL_08	prfA	peptide cha	F	3,047594	0,003609	1,849556	0,344475
POPKDL_08	tdk	thymidine k	G	-3,31187	4,14E-09	-2,53227	0,021496
POPKDL_08	pptA	4-oxalocrot	H	2,527606	0,000386	3,009584	0,047314
POPKDL_08	POPKDL_08	thiamine bi	J	-0,8905	0,003712	-1,51072	0,003106
POPKDL_08	truA	tRNA pseuc	H	-3,72936	7,99E-23	-3,75816	6,66E-10
POPKDL_08	POPKDL_08	bifunctiona	S	-3,42709	3,69E-31	-2,64167	2,84E-06
POPKDL_08	POPKDL_08	ECF transp	S	2,794451	7,31E-16	3,269852	2,74E-10
POPKDL_08	POPKDL_08	TIGR01440	M	-2,42665	9,93E-10	-3,79381	1,93E-05
POPKDL_08	POPKDL_08	mechanose	D	-1,25572	0,000185	-1,79419	0,006299
POPKDL_08	tig	trigger fact	K	-0,11351	0,751742	-0,53482	0,504056
POPKDL_08	rpoE	DNA-direct	F	1,566007	0,224763	2,272767	0,27487
POPKDL_08	pyrG	CTP syntha	T	-0,28637	0,394566	-0,51648	0,428283
POPKDL_08	POPKDL_08	sensor hist	K T	0,041065	0,946803	-0,55627	0,277881
POPKDL_08	POPKDL_08	DNA-bindin	S	-0,24825	0,52385	-0,54569	0,644886
POPKDL_08	lrgA	antiholin-lil	M	1,055805	0,019001	0,506588	0,634516
POPKDL_08	lrgB	antiholin-lil	D	0,983812	0,008497	0,37401	0,675805
POPKDL_08	frsA	alpha/beta hydrolase		-2,47167	8,49E-14	-1,72326	0,004138
POPKDL_08	fba	fructose-bi	L	1,091787	0,169715	-2,15713	0,289313
POPKDL_08	POPKDL_08	IS30 family	V	0,927604	0,009926	1,378701	0,05603
POPKDL_08	POPKDL_08	carboxypep	P	-0,46347	0,121407	-0,34641	0,60627
POPKDL_08	POPKDL_08	cation transporter		0,210009	0,679975	0,664299	0,400804
POPKDL_08	POPKDL_08	HTH tetR-ty	K	1,06918	0,000212	0,970625	0,065915

POPKDL_08	POPKDL_08	Streptococ	T	0,219072	0,51424	0,40242	0,563188
POPKDL_08	POPKDL_08	histidine ki	K T	-0,50514	0,081132	-0,51457	0,39816
POPKDL_08	POPKDL_08	DNA-binding response		0,75391	0,020466	0,293834	0,743831
POPKDL_08	POPKDL_08	K+-transpo	P	6,401635	0,001255		
POPKDL_08	kdpA	potassium-	P	-1,38578	9,74E-06	-0,86243	0,135571
POPKDL_08	kdpB	potassium-	P	0,699531	0,022011	0,783864	0,123423
POPKDL_08	kdpC	potassium-transporting		1,739639	0,000122	1,398255	0,125315
POPKDL_08	POPKDL_08	hypothetical protein		0,116775	0,915583	-1,49777	0,146404
POPKDL_08	POPKDL_08	hypothetica	G	-8,98037	1,73E-64	-7,41339	5,56E-22
POPKDL_08	POPKDL_08	Phosphogly	D	0,671997	0,027825	1,276647	0,023488
POPKDL_08	POPKDL_08	Putative extracellular protein					
POPKDL_08	POPKDL_08	septum for	J	2,099822	0,045109	2,857714	0,074195
POPKDL_08	hslR	Uncharacte	S	0,859352	0,578816	-0,5042	1
POPKDL_08	POPKDL_08	polysaccha	L	-2,97704	3,56E-27	-1,77853	0,031724
POPKDL_08	mfd	Transcriptio	J	-1,65353	7,52E-06	-1,71917	0,000942
POPKDL_08	pth	aminoacyl-	J	-0,6615	1	-1,38856	0,862941
POPKDL_08	ychF	redox-regul	K	0,057524	1	1,536825	0,245245
POPKDL_08	POPKDL_08	XRE family	S	-3,17455	2,80E-16	-1,29219	0,204103
POPKDL_08	POPKDL_08	DUF951 do	L	-0,43254	0,419635	-2,46232	0,113309
POPKDL_08	dnaN	DNA polym	L	1,412715	0,070165	0,663406	0,693569
POPKDL_08	dnaA	chromoson	K	1,699336	0,00268	1,236406	0,437463
POPKDL_08	POPKDL_08	chromoson	O	-3,91611	1,91E-15	-2,27419	0,012817
POPKDL_08	POPKDL_08	serine prot	J	0,291695	0,648987	-0,16414	0,870001
POPKDL_08	rlmH	23S rRNA (p	G	-5,09411	4,36E-30	-5,7466	2,46E-23
POPKDL_08	celC	PTS lactose	G	1,553147	2,07E-05	1,081083	0,149933
POPKDL_08	celA	PTS sugar t	G	0,567997	0,07109	0,715167	0,295578
POPKDL_08	celB	PTS cellobi	C	0,391766	0,327537	0,523871	0,554428
POPKDL_08	pflD	formate C-a	K	0,330698	0,302046	0,352759	0,603115
POPKDL_08	POPKDL_08	Transcriptio	G	-1,00868	0,000225	-1,29174	0,025487
POPKDL_08	POPKDL_08	Phosphotra	G	0,521232	0,07824	0,532198	0,374401
POPKDL_08	POPKDL_08	PTS fructos	G	-1,86777	9,84E-11	-1,98677	0,00055

POPKDL_08	POPKDL_08	PTS system	G	1,097018	0,003199	1,57343	0,000713
POPKDL_08	POPKDL_08	formate C-a	C	-2,98447	2,04E-23	-1,1632	0,214864
POPKDL_08	POPKDL_08	Glycyl-radic	H	-2,25417	5,11E-16	-1,88862	7,57E-05
POPKDL_08	talA	fructose-6-	C	-3,19577	9,09E-28	-2,77101	6,30E-07
POPKDL_08	POPKDL_08	glycerol dehydrogenase		0,778384	0,188976	-0,74209	0,672526
POPKDL_08	POPKDL_08	hypothetica	I	0,712501	0,07867	0,367202	0,694946
POPKDL_08	pgpA	phosphatid	S	-2,88661	1,67E-23	-3,08061	9,25E-08
POPKDL_08	POPKDL_08	ABC transp	S	-1,97453	6,65E-05	-2,29701	0,003058
POPKDL_08	POPKDL_08	branched-c	S	-5,17906	2,44E-16	-6,25073	3,22E-24
POPKDL_08	phnK	phosphona	S	-0,96332	0,125392	-2,56502	0,001649
POPKDL_08	rnj2	Ribonuclea	S	0,916045	0,190191	1,130966	0,383531
POPKDL_08	POPKDL_08	esterase fa	S	-0,33006	0,492865	-0,2147	0,846324
POPKDL_08	POPKDL_08	Protease	S	-5,0692	9,43E-22	-2,50209	0,024223
POPKDL_08	abcf	ABC-F type	C	-0,96756	0,000705	-1,15829	0,045372
POPKDL_08	pdhA	pyruvate de	C	0,717779	0,29084	-0,5603	0,85892
POPKDL_08	acoB	alpha-ketoa	C	-1,20363	0,020525	-1,51826	0,110281
POPKDL_08	aceF	dihydrolipo	C	-0,03241	0,904131	1,433366	0,345704
POPKDL_08	lpdA	dihydrolipoyl dehydroge		-0,25194	0,630473	-1,49439	0,211747
POPKDL_08	POPKDL_08	hypothetical protein		-1,89308	4,94E-08	-2,19337	0,07703
POPKDL_08	POPKDL_08	hypothetica	P V S	-0,32246	0,311914	0,003229	1
POPKDL_08	POPKDL_08	bacteriocin ABC transp		-0,50824	0,115357	-1,09942	0,046598
POPKDL_08	POPKDL_08	PTS fructos	G	-0,20747	0,641661	-0,36762	0,65109
POPKDL_08	sgaB	PTS maltos	S	2,513965	0,002406	2,333394	0,12602
POPKDL_08	ulaA	PTS ascorb	H	0,279722	0,673786	0,394233	0,752732
POPKDL_08	talA	transaldola	J	1,281272	0,00032	0,993277	0,200876
POPKDL_08	pnp	polyribonuc	S	-1,67982	0,000192	-1,68077	0,069225
POPKDL_08	POPKDL_08	SseB doma	E	2,628241	0,000744	1,727881	0,320269
POPKDL_08	cysE	serine O-acetyltransfer		-1,41458	0,566981	3,000201	0,076105
POPKDL_08	POPKDL_08	hypothetica	L	0,639285	0,396531	-0,67448	0,69968
POPKDL_08	POPKDL_08	DUF3991 d	L	0,669632	0,019167	0,473883	0,459814
POPKDL_08	POPKDL_08	hypothetica	U	-0,65102	0,040764	-1,04716	0,073882

POPKDL_08	POPKDL_08	relaxase Mc	L	-1,12031	6,71E-05	-1,37689	0,012816
POPKDL_08	POPKDL_08	hypothetica	F	-4,26776	6,18E-42	-4,95853	5,46E-21
POPKDL_08	POPKDL_08	DNA-entry r	S	0,854995	0,00891	1,400899	0,010357
POPKDL_08	POPKDL_08	hypothetica	S	-3,78378	4,57E-36	-3,9192	6,92E-12
POPKDL_08	POPKDL_08	hypothetica	S	0,469324	0,396434	0,521425	0,531138
POPKDL_08	POPKDL_08	hypothetica	S	-0,18489	0,651786	-0,07244	0,966799
POPKDL_08	POPKDL_08	hypothetica	S	-6,97246	5,08E-22	-5,90128	1,32E-08
POPKDL_08	POPKDL_08	hypothetica	S	0,125434	0,749714	0,26429	0,685776
POPKDL_08	POPKDL_08	hypothetica	S	0,273822	0,492865	0,26917	0,670652
POPKDL_08	POPKDL_08	hypothetica	S	-1,79156	5,44E-10	-2,08979	0,002651
POPKDL_08	POPKDL_08	hypothetica	K	0,099553	0,820115	0,319109	0,697881
POPKDL_08	POPKDL_08	RepE protei	L	-0,67455	0,030942	-0,79401	0,199606
POPKDL_08	POPKDL_08	Methyltrans	S	-0,43176	0,155659	-0,68625	0,206116
POPKDL_08	POPKDL_08	Modificatio	S	3,274577	4,46E-05	0,414144	0,862941
POPKDL_08	POPKDL_08	Modificatio	L	1,179195	0,302733	-2,69135	0,672526
POPKDL_08	POPKDL_08	DNA (cytosine-5-)-meth		-3,25038	4,43E-12	-5,18429	1,65E-06
POPKDL_08	POPKDL_08	C protein al	V	3,653539	0,002892	0,052896	1
POPKDL_08	POPKDL_08	MATE famil	K	-3,65372	9,14E-35	-3,75961	4,11E-14
POPKDL_08	POPKDL_08	MarR family	S	-0,9637	0,017309	-0,93211	0,171873
POPKDL_08	POPKDL_08	DUF1912 d	L	-4,73092	0,034186	3,048491	0,097049
POPKDL_08	POPKDL_08	HhH1 doma	E	-0,30665	0,53438	-0,88135	0,384526
POPKDL_08	POPKDL_08	VOC domai	J	-0,06472	0,931023	-1,0033	0,437463
POPKDL_08	POPKDL_08	GNAT famil	J	-0,33916	0,320667	0,437556	0,693709
POPKDL_08	POPKDL_08	Ribosomal-	F	3,200462	0,022901	-2,69135	0,672526
POPKDL_08	POPKDL_08	shikimate k	J	2,01155	7,60E-07	2,840248	8,66E-05
POPKDL_08	valS	valine--tRN	L	1,5009	0,016224	2,69176	0,015331
POPKDL_08	cas3	CRISPR-ass	S	-1,84269	2,15E-10	-2,18702	0,00031
POPKDL_08	cas5c	type I-C CR	F	2,423456	4,04E-07	2,796434	1,18E-08
POPKDL_08	hprK	HPr(Ser) kir	M	0,94431	0,204222	1,272048	0,379825
POPKDL_08	lgt	prolipoprot	S	-0,98101	0,002443	-1,88682	0,0021
POPKDL_08	POPKDL_08	DUF948 do	S	-6,75155	7,27E-38	-7,02121	2,99E-16

POPKDL_08	POPKDL_08	YtxH domai	S	-0,55555	0,128408	-0,30754	0,689188
POPKDL_08	POPKDL_08	DUF3270 d	O	0,946471	0,49423	-4,11916	0,200876
POPKDL_08	rlhA	peptidase U	O	-1,3239	1,25E-05	-1,11477	0,028059
POPKDL_08	rlhA	collagenase	S	-3,73556	9,17E-36	-4,09431	1,65E-12
POPKDL_08	POPKDL_08	PC4 domai	S	-0,10455	0,751742	-0,43104	0,541971
POPKDL_08	yigB	FMN and 5-	J	-0,53165	0,134419	-1,27244	0,016851
POPKDL_08	lysS	lysine--tRNA ligase		1,127541	0,09622	0,222672	0,899426
POPKDL_08	POPKDL_08	Integrase catalytic dom		1,768331	0,000199	2,060576	0,006657
POPKDL_08	POPKDL_08	aminoacyl-tRNA deacyl		0,189642	0,560944	-0,19019	0,796693
POPKDL_08	POPKDL_08	serine hydr	D	-0,3224	0,536665	-1,75953	0,181176
POPKDL_08	tilS	tRNA(Ile)-ly	F	0,503512	0,62205	0,334948	0,79863
POPKDL_08	hpt	hypoxanthi	O	-4,49088	9,80E-17	-5,13661	4,19E-06
POPKDL_08	ftsH	ATP-depend	E	0,501807	0,416377	-0,21798	0,982682
POPKDL_08	POPKDL_08	amino acid permease		-1,39155	0,03243	-1,75069	0,067163
POPKDL_08	POPKDL_08	Putative transposase re		1,968902	0,241674	3,421839	0,0203
POPKDL_08	POPKDL_08	Putative transposase re		-0,33482	1	-4,82253	0,052248
POPKDL_08	POPKDL_08	IS66 family transposase		-0,72914	0,011987	-0,90353	0,092045
POPKDL_09	POPKDL_09	Tyrosine rec	J	0,859731	0,005554	0,312283	0,695827
POPKDL_09	rpsI	30S ribosor	J	6,398254	0,000417		
POPKDL_09	rplM	50S ribosor	K	1,771501	0,147804	3,489544	0,033804
POPKDL_09	xRE	transcriptic	S	-5,45552	4,34E-10	-8,12629	5,18E-06
POPKDL_09	POPKDL_09	Putative membrane spa		-2,70905	5,54E-21	-3,43135	3,71E-07
POPKDL_09	POPKDL_09	Fatty acid-binding prote		1,209275	1,55E-05	1,102174	0,035696
POPKDL_09	rae1	DNA-binding protein		0,65461	0,027024	0,573148	0,330765
POPKDL_09	rlmB	23S rRNA (guanosine(2		-0,58761	0,135562	-0,80088	0,203254
POPKDL_09	POPKDL_09	hypothetical protein		0,050019	0,895066	0,168179	0,810868
POPKDL_09	POPKDL_09	hypothetica	J	0,89406	0,137935	1,004643	0,354281
POPKDL_09	POPKDL_09	Mini-ribonu	J	-1,89595	1,37E-08	-2,74228	9,43E-05
POPKDL_09	cysS	cysteine--tl	S	1,299152	0,061824	2,169053	0,109512
POPKDL_09	POPKDL_09	hypothetical protein		5,123989	0,032024		
POPKDL_09	yloU	Uncharacte	S	5,044838	0,046718		

POPKDL_09	yloV	DAK2 doma	O	1,215139	0,058624	1,923816	0,198316
POPKDL_09	hflC	SPFH domain-containi		-0,29343	0,335407	-0,36583	0,623134
POPKDL_09	POPKDL_09	hypothetica	E	-3,25072	0,377302	-2,69135	0,672526
POPKDL_09	glnQ	ABC transp	P	-4,49476	1,08E-22	-5,2391	1,51E-10
POPKDL_09	hisJ	glutamine A	L	1,265755	0,035266	1,012986	0,262143
POPKDL_09	POPKDL_09	Transposas	F	0,734254	0,043251	0,289529	0,771542
POPKDL_09	pbuX	xanthine pe	F	0,633944	0,314469	1,442457	0,198316
POPKDL_09	xpt	xanthine ph	F	0,868137	0,467625	1,909225	0,129356
POPKDL_09	guaC	GMP reduct	P	0,338658	0,382338	0,042173	0,979632
POPKDL_09	POPKDL_09	Glycine bet	E	1,145874	8,86E-05	1,768478	0,000782
POPKDL_09	opuBA	glycine/betaine ABC tra		-3,11417	1,87E-18	-2,5678	9,35E-07
POPKDL_09	POPKDL_09	ABC transporter substr		-1,02602	0,002212	-1,2436	0,02312
POPKDL_09	glnA	type I glutamate--amm		-1,48226	0,094825	-2,36217	0,087971
POPKDL_09	soxR	MerR family transcripti		3,101959	0,032638	-2,69135	0,672526
POPKDL_09	POPKDL_09	Aromatic acid exporter		-1,2101	0,00092	-2,53112	5,63E-06
POPKDL_09	pgk	Phosphoglycerate kina		2,043902	0,008568	0,530677	0,920386
POPKDL_09	POPKDL_09	protein jag		1,470036	0,085227	1,686484	0,326732
POPKDL_09	yidC1	Membrane protein inse		2,023239	0,003147	2,459349	0,062307
POPKDL_09	rnpA	ribonuclease P protein		5,794616	0,00177	7,855446	0,001612
POPKDL_09	FasX		K T	0,596359	0,467137	1,227432	0,386247
POPKDL_09	lytT	DNA-bindir	T	1,063582	0,00513	2,13406	0,024413
POPKDL_09	POPKDL_09	histidine kinase		0,355704	0,378075	1,441055	0,098249
POPKDL_09	POPKDL_09	histidine kinase		-0,07216	0,884387	1,00625	0,229022
POPKDL_09	POPKDL_09	hypothetical protein		-1,07893	0,060216	-3,02191	0,059333
POPKDL_09	POPKDL_09	helix-turn-h	S	-5,13232	0,013278	-1,77981	0,631588
POPKDL_09	POPKDL_09	replication protein Rep		-0,93069	0,00324	-1,22959	0,038036
POPKDL_09	POPKDL_09	hypothetical protein		-1,02445	0,002351	-0,57888	0,353629
POPKDL_09	POPKDL_09	cell division protein Fts		0,114594	0,743906	-0,28463	0,693709
POPKDL_09	POPKDL_09	hypothetical protein		2,83724	1,82E-14	3,538223	4,21E-11
POPKDL_09	POPKDL_09	hypothetical protein		0,65675	0,159786	-0,17088	0,940809
POPKDL_09	POPKDL_09	Helix-turn-helix domair		0,754286	0,04258	0,528325	0,447988

POPKDL_09	POPKDL_09	hypothetical protein	2,50753	4,72E-15	2,78659	1,64E-07
POPKDL_09	POPKDL_09	hypothetical protein	-1,83284	1,06E-11	-1,86903	0,000342
POPKDL_09	POPKDL_09	Restriction endonuclea	0,090245	0,790691	-0,34487	0,719078
POPKDL_09	POPKDL_09	Site-specific DNA-meth	-1,09688	9,61E-05	-0,82605	0,205857
POPKDL_09	POPKDL_09	DUF4300 domain-cont	-0,58341	0,141179	-1,33235	0,017399
POPKDL_09	POPKDL_09	hypothetical protein	1,602434	0,321284	5,215802	0,007336
POPKDL_09	MOSES4		1,602623	0,321284	5,215767	0,007369
POPKDL_09	dfsB	cytoplasmic protein	-3,73783	1,77E-36	-3,20649	3,79E-07
POPKDL_09	POPKDL_09	YSIRK-targeted surface	0,956024	0,002379	1,325711	0,016211
POPKDL_09	POPKDL_09	hypothetical protein	-0,0392	0,925719	0,030565	0,984973
POPKDL_09	POPKDL_09	hypothetical protein			6,230352	0,030935
POPKDL_09	POPKDL_09	LytTR family transcripti	0,933729	0,019834	0,021194	1
POPKDL_09	POPKDL_09	hypotheticaS	0,546773	0,155623	-0,3597	0,779531
POPKDL_09	POPKDL_09	Lantibiotic J	1,587007	9,72E-06	2,313592	0,003593
POPKDL_09	gltX	glutamate--tRNA ligase	0,294872	0,649759	0,325508	0,85676
POPKDL_09	POPKDL_09	hypothetical protein	0,289044	0,651786	-0,73111	0,504495
POPKDL_09	POPKDL_09	hypothetical protein	0,220141	0,503857	0,532483	0,374401
POPKDL_09	POPKDL_09	hypothetical protein	-1,37165	1,99E-06	-1,93727	0,000138
POPKDL_09	POPKDL_09	DUF6287 domain-cont	-1,77184	2,28E-07	-0,93364	0,168385
POPKDL_09	POPKDL_09	IS1182 family transpos	-0,7323	0,013743	-0,81617	0,165122
POPKDL_09	POPKDL_09	HTH LytTR-type domain	-4,0931	2,71E-18	-3,98256	4,09E-05
POPKDL_09	POPKDL_09	DUF3021 d L S X	-1,03228	0,004979	-1,35064	0,049107
POPKDL_09	POPKDL_09	Putative transposase re	-2,94366	8,68E-23	-2,91329	6,18E-09
POPKDL_09	POPKDL_09	AbrB/MazE/SpoVT fami	5,121018	0,04613	6,488734	0,021496
POPKDL_09	POPKDL_09	type II toxin-antitoxin sy	0,727596	0,024767	0,374576	0,658976
POPKDL_09	POPKDL_09	Transposase-IS1562	-0,76208	0,00879	-0,27263	0,693835
POPKDL_09	POPKDL_09	IS3 family transposase	-0,76211	0,007916	-0,78717	0,183365
POPKDL_09	POPKDL_09	Transposase	0,662339	0,023064	0,356152	0,634516
POPKDL_09	POPKDL_09	DEDD-Tnp-IS110 doma	-0,29205	0,377302	0,27944	0,719903
POPKDL_09	POPKDL_09	IS110 family transposa	0,735726	0,011519	1,592478	0,007217
POPKDL_09	rpmB	50S ribosomal protein l	0,982674	0,365794	0,933471	0,78672

POPKDL_09	POPKDL_09	hypothetical protein	-1,75569	9,84E-10	-1,83807	0,00119
POPKDL_09	POPKDL_09	IS982 family transposa	-3,21286	5,09E-10	-1,44776	0,107801
POPKDL_09	POPKDL_09	transposase	-0,74034	0,010619	-0,91842	0,1044
POPKDL_09	POPKDL_09	Transposase	2,437721	9,22E-10	2,653689	7,25E-07
POPKDL_09	POPKDL_09	Transposase	-1,70678	3,11E-07	-1,84092	0,021496
POPKDL_09	POPKDL_09	Integrase catalytic dom	1,120848	6,40E-05	0,918979	0,089974
POPKDL_09	MOSES4		1,152449	0,000458	2,11449	0,000757
POPKDL_09	POPKDL_09	Transposase	1,384783	6,38E-06	1,942252	0,00011
POPKDL_09	MOSES4		0,757437	0,015869	0,758904	0,240907
POPKDL_09	POPKDL_09	Integrase	-0,16064	0,745441	-1,06476	0,470918
POPKDL_09	POPKDL_09	hypothetical protein	-4,17287	0,13955	-0,83871	1
POPKDL_09	POPKDL_09	hypothetical protein	5,997636	0,007393	4,32445	0,214864
POPKDL_09	POPKDL_09	transposase	-4,1374	0,002242	-1,08046	0,634516
