# Supplementary material for: Discovery of a new fibronectin-binding surface protein of Streptococcus canis with serum opacification activity through transposon directed insertion-site sequencing
Source: Front Cell Infect Microbiol. 2026 Jun 29;16:1867913. doi: 10.3389/fcimb.2026.1867913 (PMC13357522; doi:10.3389/fcimb.2026.1867913)
Supplement: Supplementary Table 2 — List of transposon mutants of 94 genes that fit the logFC>2 and q<0.01 conditions after the second infection selection step. [file DataSheet2.pdf]

				First Infection		Second Infection	
**locus_tag**	**gene_name**	**function**	**COG**	**logFC**	**q.value**	**logFC**	**q.value**
POPKDL_0(	purN	phosphorib	F	1,85932	0,053437	3,751014	0,0052
POPKDL_0(	adhE	bifunctiona	C	1,737296	0,000162	4,009893	7,45E-07
POPKDL_0(	fsa	fructose-6-	H	1,392901	0,003744	3,176035	0,001293
POPKDL_0(	POPKDL_0(	Uncharacte	T?	2,003865	1,11E-06	3,724564	3,32E-07
POPKDL_0(	nusA	transcriptic	K	0,313288	0,67784	3,359131	0,008816
POPKDL_0:	POPKDL_0:	rhodanese-	P	5,910762	0,001754	7,519719	0,00526
POPKDL_0:	POPKDL_0:	haloacid de	S	1,949797	0,00217	2,226198	0,000128
POPKDL_0:	fepC	iron ABC tra	H P	2,359467	7,72E-08	3,111707	7,22E-08
POPKDL_0:	fepD	ABC transp	P	1,891074	1,44E-12	4,032055	0,000105
POPKDL_0:	ycgQ	TIGR03943	S	2,716352	4,56E-09	2,766584	5,95E-06
POPKDL_0:	thrS	threonine--	J	2,274331	0,024767	3,461753	0,00295
POPKDL_0:	POPKDL_0:	alpha-amyl	G	2,438313	2,80E-12	3,251431	5,32E-11
POPKDL_0:	POPKDL_0:	ABC transp	V	2,201262	9,88E-11	2,332983	6,10E-07
POPKDL_0:	POPKDL_0:	transglutan	D	2,52072	1,33E-10	3,441697	1,64E-08
POPKDL_0:	mtnN	5'-methylth	E	0,827611	0,622888	4,111069	0,006606
POPKDL_0:	POPKDL_0:	cysteine m(	L	2,601619	2,29E-10	2,6157	0,000909
POPKDL_0:	POPKDL_0:	signal pepti	S	1,703058	3,19E-05	2,318033	9,49E-05
POPKDL_0:	rsuA	pseudourid	J	1,760537	5,82E-07	2,545902	2,16E-05
POPKDL_0:	racE	glutamate r	M?	2,165511	0,063892	4,461849	0,00868
POPKDL_02	POPKDL_02	streptolysir	S	1,645368	2,75E-05	2,271716	2,09E-05
POPKDL_02	coaD	pantethein(	H	0,978079	0,365642	5,053028	0,001422
POPKDL_02	POPKDL_02	ribose-5-ph	S	1,427367	1,67E-06	3,716773	7,29E-05
POPKDL_02	ykuJ	DUF1797 d	S	3,531175	0,002334	4,582309	0,004699
POPKDL_02	POPKDL_02	TIGR02206	M	2,033617	3,69E-07	2,571694	1,49E-07
POPKDL_02	pepT	peptidase T	E	2,121455	2,33E-07	3,891013	3,09E-06
POPKDL_02	POPKDL_02	Pore formir	S	2,115592	2,27E-11	2,315021	5,63E-06
POPKDL_02	POPKDL_02	LysM peptic	M	1,880364	5,13E-10	2,477408	2,16E-05
POPKDL_03	POPKDL_03	hypothetical protein		0,949343	0,001342	3,527009	0,000193
POPKDL_03	POPKDL_03	Virulence fa	D	2,098902	4,19E-11	4,560741	3,11E-06

Conditions in second infection

log FC>2

q<0.01

POPKDL_0:	POPKDL_0:	Disulfide-b	H	2,987621	1,40E-12	3,751138	3,88E-12
POPKDL_0:	POPKDL_0:	PTS fructos	G	0,691107	0,064403	2,085284	0,005892
POPKDL_0:	POPKDL_0:	PTS fructos	G	2,356055	2,98E-07	2,756166	3,05E-07
POPKDL_0:	POPKDL_0:	Putative me	S	1,849202	2,43E-05	2,232243	3,38E-05
POPKDL_0:	queG	tRNA epoxy	C	1,754701	1,23E-07	2,100037	7,31E-05
POPKDL_0:	POPKDL_0:	fructose-bi:	G	0,316931	0,377826	2,266322	0,009462
POPKDL_0:	POPKDL_0:	hypothetical protein		3,252876	1,30E-11	3,538167	8,95E-10
POPKDL_0:	dinB	DNA polym	L	2,348735	4,74E-08	2,650617	7,92E-05
POPKDL_0:	mutY	A/G-specifi	L	0,641805	0,041612	2,024095	0,00295
POPKDL_0:	POPKDL_0:	DUF2130 d	S	1,9344	6,33E-09	2,322297	9,98E-06
POPKDL_0:	pdpdeoA	pyrimidine-	F	1,611455	1,53E-07	2,108645	2,09E-05
POPKDL_0:	med	BMP family	S	0,995058	0,001112	2,396998	0,000318
POPKDL_0:	ccmA	heme ABC (	S	1,054086	0,000435	3,2273	0,000158
POPKDL_0‹	POPKDL_0‹	PLP-dependent aminot		1,588316	2,35E-05	2,104049	0,000274
POPKDL_0‹	POPKDL_0‹	Protein ess	C	0,270324	0,465525	2,524924	0,008481
POPKDL_0‹	POPKDL_0‹	hypothetical protein		2,767653	8,56E-09	3,388958	6,85E-13
POPKDL_0‹	POPKDL_0‹	farnesyl pyı	H	2,457962	4,22E-07	3,941158	1,05E-08
POPKDL_0‹	POPKDL_0‹	Fibronectin	D?	0,311017	0,335407	2,472691	0,007272
POPKDL_0‹	POPKDL_0‹	hypothetical protein		5,531298	0,005448	6,61068	0,006657
POPKDL_0‹	POPKDL_0‹	DNA-bindir	K	1,463641	2,54E-05	2,497315	0,000252
POPKDL_0‹	POPKDL_0‹	polyprenyl :	H	0,989016	0,010908	2,187155	0,000245
POPKDL_0‹	POPKDL_0‹	NADH dehy	C	0,715499	0,015687	4,065477	0,00031
POPKDL_0‹	POPKDL_0‹	Glutaconyl-	I	2,793278	1,18E-07	3,494702	3,29E-11
POPKDL_0‹	POPKDL_0‹	dihydrofola	C H	1,726116	4,09E-06	2,225264	3,79E-05
POPKDL_0‹	proW	glycine/bet	E	2,506273	6,51E-09	3,026523	2,65E-08
POPKDL_0!	scm2	M-like prote	K T	1,973994	2,29E-05	2,352531	1,55E-06
POPKDL_0!	POPKDL_0!	Drug resista	L	2,474527	9,34E-10	3,237304	4,80E-08
POPKDL_0!	purR	pur operon	D	3,010459	1,38E-22	7,627408	4,80E-08
POPKDL_0!	POPKDL_0!	Phosphoryl	F	2,421414	9,94E-10	2,434826	8,66E-05
POPKDL_0!	POPKDL_0!	DUF624 do	T	1,283688	5,46E-05	2,026109	0,000132
POPKDL_0!	POPKDL_0!	competenc	U	1,0624	0,000281	2,21636	0,002951

POPKDL_0!	dusB	tRNA dihyd	O	1,789738	1,61E-05	3,303707	0,000104
POPKDL_0(	deoB	phosphope	F	-0,31125	0,43815	2,848453	0,009484
POPKDL_0(	POPKDL_0(	hypothetica	G	2,943866	4,29E-16	3,265031	3,84E-07
POPKDL_0(	POPKDL_0(	DUF3284 d	G	1,583515	0,00486	2,620158	0,003879
POPKDL_0(	POPKDL_0(	LuxR family	E	1,108933	0,004506	2,810136	5,27E-05
POPKDL_0(	atoA	Acyl-CoA:a	S	1,704253	7,12E-08	2,375356	5,54E-06
POPKDL_0(	POPKDL_0(	DNA-bindir	J	2,46003	3,05E-17	4,579658	1,83E-06
POPKDL_0(	POPKDL_0(	V-type ATP	C	2,10381	5,33E-10	2,136162	0,004138
POPKDL_0(	brnQ	branched-c	P	-0,13936	0,736449	2,467173	0,006657
POPKDL_0(	mdlB	multidrug A	V	0,763818	0,011936	2,226394	0,000622
POPKDL_0(	POPKDL_0(	rhomboid fa	H	2,161311	1,03E-08	2,632191	2,80E-06
POPKDL_0	POPKDL_0	5-formyltet	K	1,479158	1,23E-05	2,277158	0,002212
POPKDL_0	radC	DNA repair	T	2,410915	1,52E-14	2,99396	1,36E-07
POPKDL_0	hflX	GTPase HflX	G	1,211629	1,25E-05	2,616735	0,000127
POPKDL_0	yqjQ	short-chain	L	0,657409	0,030468	2,936893	0,000274
POPKDL_0	POPKDL_0	FAD-depen	M	0,807032	0,024963	2,465009	0,003598
POPKDL_0	rfbA	glucose-1-p	M	6,081559	0,000382	6,787043	0,00234
POPKDL_0	POPKDL_0	hypothetical protein		2,565477	3,92E-09	2,839224	0,000545
POPKDL_0	trpS	tryptophan-	U	5,328271	0,00855	7,334342	0,000139
POPKDL_0	glcU	Putative su	S	1,280265	0,057923	3,256263	0,003906
POPKDL_0	POPKDL_0	Immunoglo	K	0,473291	0,1759	8,427491	3,55E-05
POPKDL_0	pepF	oligoendop	H	2,435229	8,23E-08	2,93425	6,52E-08
POPKDL_0{	POPKDL_0{	hypothetical protein		1,688065	1,09E-07	2,130557	0,000104
POPKDL_0{	POPKDL_0{	DNA (cytosine-5-)-meth		2,357753	3,63E-07	3,2944	1,79E-09
POPKDL_0{	POPKDL_0{	ECF transp	S	2,794451	7,31E-16	3,269852	2,74E-10
POPKDL_0{	POPKDL_0{	shikimate k	J	2,01155	7,60E-07	2,840248	8,66E-05
POPKDL_0{	cas5c	type I-C CR	F	2,423456	4,04E-07	2,796434	1,18E-08
POPKDL_0{	POPKDL_0{	Integrase catalytic dom		1,768331	0,000199	2,060576	0,006657
POPKDL_0!	rnpA	ribonuclease P protein		5,794616	0,00177	7,855446	0,001612
POPKDL_0!	POPKDL_0!	hypothetical protein		2,83724	1,82E-14	3,538223	4,21E-11
POPKDL_0!	POPKDL_0!	hypothetical protein		2,50753	4,72E-15	2,78659	1,64E-07

POPKDL_09	POPKDL_09	hypothetical protein	1,602434	0,321284	5,215802	0,007336
POPKDL_09	POPKDL_09	Lantibiotic J	1,587007	9,72E-06	2,313592	0,003593
POPKDL_09	POPKDL_09	Transposase	2,437721	9,22E-10	2,653689	7,25E-07
