# Supplementary material for: Discovery of a new fibronectin-binding surface protein of Streptococcus canis with serum opacification activity through transposon directed insertion-site sequencing
Source: Front Cell Infect Microbiol. 2026 Jun 29;16:1867913. doi: 10.3389/fcimb.2026.1867913 (PMC13357522; doi:10.3389/fcimb.2026.1867913)
Supplement: Supplementary Table 3 — List of LPxTG anchoring-domain containing genes with their corresponding logFC and q values. [file DataSheet3.pdf]

**locus_tag**	**gene_name**	**function**	**COG**	**logFC**	**q.value**
POPKDL_01	POPKDL_01	serine prote	O	1,564724	0,024415
POPKDL_01	POPKDL_01	peptidase S	O	-1,3966	0,002091
POPKDL_02	POPKDL_02	Peptidoglyc	D	1,050779	0,110805
POPKDL_02	POPKDL_02	GRAM-POS-ANCHORIN		0,406232	0,558042
POPKDL_03	purR	transcriptic	K	1,717303	0,020291
POPKDL_03	POPKDL_03	aminopepti	E	0,484305	0,397433
**POPKDL_04**	**POPKDL_04**	**Fibronectin binding pr**		**2,472691**	**0,007272**
POPKDL_04	POPKDL_04	amidase	S	0,220339	0,777537
**POPKDL_05**	**scm2**	**M-like prot**	**K T**	**2,352531**	**1,55E-06**
POPKDL_05	POPKDL_05	amylopullu	C O	-2,03405	0,000192
POPKDL_05	POPKDL_05	alpha-mannosidase		-3,36954	9,08E-10
POPKDL_05	POPKDL_05	Fibronectin	M	0,030669	0,984973
POPKDL_06	POPKDL_06	Emm-like c	E	0,810529	0,193728
POPKDL_06	POPKDL_06	Collagen-like surface p		0,183682	0,85892
POPKDL_06	POPKDL_06	Ferrous iron	I	-1,44893	0,02372
POPKDL_07	POPKDL_07	Agglutinin receptor		1,007676	0,08116
POPKDL_07	POPKDL_07	cell wall anchor protein		-7,41998	8,42E-23
POPKDL_07	POPKDL_07	peptidase C39		-6,31772	4,11E-14
**POPKDL_07**	**POPKDL_07**	**Immunoglo**	**K**	**8,427491**	**3,55E-05**
POPKDL_07	POPKDL_07	beta-galactosidase		-1,13027	0,060911
POPKDL_08	POPKDL_08	C protein al	V	0,052896	1
POPKDL_09	hisJ	glutamine A	L	1,012986	0,262143
POPKDL_09	POPKDL_09	hypothetical protein		4,32445	0,214864
